# Comparison of count-based and clustering definitions of multimorbidity and their association with prevalence of multimorbidity, health profiles, and mortality: A cohort study of UK Biobank participants

**DOI:** 10.1371/journal.pmed.1004914

**Published:** 2026-06-12

**Authors:** Gabriella C. Silva, Aurore Fayosse, Louis Jacob, Séverine Sabia, Archana Singh-Manoux, Benjamin Landré

**Affiliations:** 1 Université Paris Cité, Inserm U1153, CRESS, Epidemiology of Ageing and Neurodegenerative Diseases, Paris, France; 2 Department of Physical Medicine and Rehabilitation, Université Paris Cité, Assistance Publique – Hôpitaux de Paris (AP-HP), Lariboisière-Fernand Widal Hospital, Paris, France; 3 Research and Development Unit, Parc Sanitari Sant Joan de Déu, CIBERSAM, ISCIII, Barcelona, Spain; 4 Faculty of Brain Sciences, University College London, London, United Kingdom; South African Medical Research Council, SOUTH AFRICA

## Abstract

**Background:**

Multimorbidity, the presence of several chronic conditions, is linked to higher mortality and healthcare use and thus poses a major challenge for aging populations. While most studies rely on simple counts of conditions, clustering approaches have been proposed to describe patterns of co-occurring diseases. We aimed to evaluate the extent to which these methodological choices influence prevalence and association with health profiles and mortality.

**Methods and findings:**

Using UK Biobank baseline data (*n* = 474,397), collected between 2006 and 2010, we compared six count-based definitions of multimorbidity based on different condition lists (extended, most prevalent, or body systems) and thresholds (≥2 versus ≥3 conditions). We also applied a clustering analysis to characterize subtypes of multimorbidity among participants with at least two chronic conditions. We compared prevalence and associations with concurrent health outcomes (polypharmacy, self-rated health, frailty, falls, surgery, chronic pain), blood-based measures (C-reactive protein, Cystatin-C, HDL, LDL Cholesterol, IGF-1), and 3- and 10-year mortality risks. Analyses were undertaken separately in men and women using multivariable regression models adjusted for sociodemographic characteristics and body mass index. Multimorbidity prevalence ranged from 1.0% (cluster-based) to 35.3% (count-based). Count-based definitions using lists with more conditions yielded higher prevalence. Higher thresholds identified more severe health profiles on all measured health outcomes, blood-based measures, but not higher mortality risks. Associations with blood-based measures were more pronounced using clustering, with the highest differences from the standard definition distributed across clusters. Odds ratios for 3-year mortality ranged from 1.44 [1.26; 1.64] to 4.60 [3.73; 5.62] for men and 1.35 [1.07; 1.69] to 3.83 [2.78; 5.14] for women. For 10-year mortality, they ranged from 1.42 [1.34; 1.50] to 3.86 [3.46; 4.30] in men and 1.29 [1.21; 1.39] to 3.33 [2.93; 3.77] for women, with clustering identifying groups with low prevalence and high mortality risks. Findings should be interpreted in light of the selected nature of the UK Biobank cohort and the cross-sectional assessment of several health indicators.

**Conclusion:**

Operational definitions of multimorbidity substantially influence prevalence estimates, while associations with mortality appear more robust across count-based approaches. Clustering analyses provide complementary insights into heterogeneity within multimorbid populations. Future translational studies are warranted to determine how multimorbidity definitions can be optimized to ultimately improve clinical management and health outcomes in practice.

## Author summary

**Why was the study done?:**

- Multimorbidity is associated with poorer health outcomes, greater healthcare use, and higher mortality, but there is no consensus on how it should be defined and measured across studies.
- Although both count-based and cluster-based approaches are widely used, few studies have systematically compared these definitions within the same population to assess how methodological choices influence prevalence estimates, but also associations with health outcomes and mortality.

**What did the researchers do and find?:**

- Using data from 474,397 UK Biobank participants, we compared six count-based definitions of multimorbidity and a cluster-based approach, and examined the prevalence of multimorbidity, their associations with health outcomes, blood-based measures, and 3- and 10-year mortality.
- Multimorbidity prevalence varied substantially depending on the definition used, whereas associations with mortality were relatively consistent across count-based definitions; clustering approaches additionally identified smaller subgroups with distinct disease profiles, poorer health status, and elevated mortality risks.

**What do these findings mean?:**

- These findings highlight that the way multimorbidity is defined strongly shapes epidemiological estimates and population characterization, underscoring the need for future translational studies to determine which definitions are most useful for improving patient management and care in practice.
- The study should also be interpreted in light of certain limitations, including the selected nature of the UK Biobank cohort, the use of some self-reported measures, and the cross-sectional assessment of several health indicators.

## Abstract 3

In this cohort study, Gabriella Silva and colleagues evaluate different definitions of multimorbidity (count-based versus clustering) and their associations with prevalence, health profiles and mortality in UK Biobank participants.

## Introduction

Multimorbidity, defined as the presence of multiple chronic conditions in an individual, is increasingly used to describe and understand transitions in health, notably in aging populations. Individuals with multimorbidity consistently experience poorer health outcomes, including higher risks of mortality [1–3], dementia [4,5], frailty [6], and lower quality of life [7]. They also engage more frequently with healthcare systems, leading to higher service utilization and costs [1,2,8,9].

An umbrella review found that the estimated prevalence of multimorbidity ranges widely, from 3.5% to 100% [10]. Besides differences in study populations, the lack of standardization in the operational definition of multimorbidity contributes to substantial variability in prevalence estimates and associations with health outcomes. This inconsistency limits the generalizability of findings, hindering a better understanding of the impact of multimorbidity and the identification of effective interventions or strategies.

The most common approach to measuring multimorbidity involves totaling the number of conditions from a predefined list [11]. Lack of consensus on the list of conditions that should be used has led to extensive variability in the number and type of conditions included in count-based approaches [12–14], ranging from 2 to 285 according to a recent systematic review [15]. Previous studies relied on self-reported conditions and access to electronic health records has expanded these lists, encoded using different classification schemes with varying levels of granularity [16,17]. This lack of harmonization has contributed to discrepancies in multimorbidity prevalence estimates across studies [18]. Counts of individual conditions or alternatively, counts of affected body systems, generally use a threshold of two or more conditions to define multimorbidity; larger cut-offs such as three or four conditions are considered to identify multimorbid populations with greater needs [11,16,17].

An alternative to count-based for characterizing multimorbidity patterns is clustering. This approach has been used to identify patterns of co-occurring conditions and to describe groups of individuals with similar disease profiles, which may help inform the understanding of shared risk factors and potential implications for patient management [7,19–22]. However, a standardized approach to identify the clusters is lacking [2].

While previous studies have conceptually compared the strengths and limitations of count-based versus clustering approaches to defining multimorbidity [8], few have simultaneously evaluated these methods in large-scale studies [20,23]. Despite the widespread use of multimorbidity measures, to our knowledge, no study has systematically compared commonly used definitions, including both count-based and cluster-based approaches, within the same population to assess how these methodological choices influence prevalence estimates and associations with health outcomes. Such differences may have important clinical and public health implications, particularly for the identification of patient profiles and for informing interventions related to resource allocation and the implementation of healthcare strategies at different levels.

The primary aim of this study is to assess how alternative, commonly used definitions of multimorbidity, including both count-based and clustering methods, affect prevalence estimates and associations with objective blood-based measures, concurrent health outcomes, and subsequent mortality.

## Methods

### Study population and design

The UK Biobank (UKB) is a population-based cohort study that recruited over 500,000 participants, aged 40–69 years, between 2006 and 2010 [24]. At baseline, participants completed an interview, clinical examination, and provided biological samples. Written informed consent was obtained from all participants and ethical approval for the UKB study was granted by the NHS North West Centre for Research Ethics Committee (reference 11/NW/0382). This research used UKB Resource under data sharing application 96856.

### Chronic conditions

We identified 38 conditions, spanning 13 ICD-10 (International Classification of Diseases) chapters, using the list defined by Ronaldson and colleagues [25] (Table A in S1 File). Similar versions of this list have been used in other UKB studies; we made minor modifications to it to account for the availability of data [3,6]. Any chronic conditions occurring prior to initial assessment were ascertained using electronic health records (primary care, hospital admissions, cancer registry, death register) and self-reported data, while excluding conditions reported solely through self-report.

### Concurrent health outcomes, blood-based measures, and mortality

Health profiles were determined using a set of concurrent health outcomes and blood-based measures.

*Health outcomes* were assessed at baseline and included polypharmacy (use of five or more medications), poor self-rated health (where participants reported their health as “poor” compared to other responses), Fried’s frailty phenotype [26] (three or more impaired criteria as defined by Jiang and colleagues [27]), reported falls in the past year, prior surgery (any recorded operations or procedures in hospital records), and chronic pain (pain lasting more than three months in any area—back, facial, general, headache, hip, knee, neck/shoulder, or stomach/abdominal).

*Blood-based measures* were measured from blood samples collected at baseline [28]. We included C-reactive protein (CRP) (mg/L), Cystatin C (mg/L), HDL cholesterol (mmol/L), IGF-1 (nmol/L), and LDL cholesterol (mmol/L), known to be associated with ageing processes [29–32]. Blood-based measures were standardized (mean = 0, standard deviation, SD = 1) separately for men and women prior to analysis.

Mortality over the follow-up was ascertained through November 30, 2022 using the national mortality register. We examined mortality risk at 3 and 10 years.

### Covariates

Sociodemographic variables included age, sex (male/female), Townsend deprivation index [33], and reported ethnicity (White/Non-White). We additionally included body mass index (BMI), calculated using measured height and weight.

### Statistical analyses

We implemented six count-based definitions of multimorbidity and a clustering analysis used to characterize subtypes of multimorbidity among participants with multiple chronic conditions.

For count-based measures, we considered three variations of the condition list (Table B in S1 File). Count All included all 38 identified conditions, Count MostPrev focused on the 12 most prevalent conditions, as recommended by Fortin and colleagues [16], and Count Syst counted the number of body organ systems involved, based on the ICD-10 chapter classification [17]. For each list, we used two thresholds to define multimorbidity: having two or more conditions (vs. 0 or 1) and three or more conditions (vs. 0, 1, or 2). These are the thresholds most frequently reported in the literature [15]. For the clustering approach, we replicated the method proposed by Violan and colleagues [34] separately in men and women with at least two conditions according to the Count All measure. Details on the clustering method are provided in S1 Methods: Cluster Analyses. Briefly, we performed a multiple correspondence analysis followed by k-means clustering on the resulting dimensions.

Subsequent analyses using the six multimorbidity definition and the clustering approach were composed of (1) comparison of multimorbidity prevalence, (2) associations with health profiles, and (3) mortality risk at the 3- and 10-year horizon. All analyses were undertaken separately in men and women.

*Comparison of prevalence:* For each multimorbidity definition, we calculated the prevalence of multimorbidity, as well as the mean (and SD) condition count and age for multimorbid and non-multimorbid participants. We also reported the prevalence of multimorbidity by age groups defined using terciles (38–53, 53–61, and 61–73 years old).

*Cross-sectional associations with concurrent health outcomes and blood-based measures:* For the six health outcomes, we used separate multivariable logistic regression models and reported the odds ratio (OR), along with 95% confidence intervals, comparing those identified as multimorbid (or identified as belonging to a particular multimorbidity cluster) to those not identified as multimorbid. Adjustment variables included sociodemographic variables and BMI. We compared biological profiles using objective blood-based measures in participants with or without multimorbidity. We used multivariable linear regression models adjusted for the same set of covariates.

*Associations with mortality:* The association of multimorbidity with mortality was examined at two different clinically relevant fixed horizon, 3 and 10 years of follow-up, using multivariable logistic regression, adjusting for sociodemographic factors and BMI. In secondary analysis, we used a Cox model based on time-to-event data to verify whether the conclusions also applied.

Analyses were conducted using R (version 4.5.1) [35]. The multiple correspondence analysis used for clustering was completed using the mjca function in the ca package (version 0.71.1) [36]. This study is reported as per the Strengthening the Reporting of Observational Studies in Epidemiology (STROBE) guideline (S1 Checklist).

## Results

### Sample

**Fig 1:**
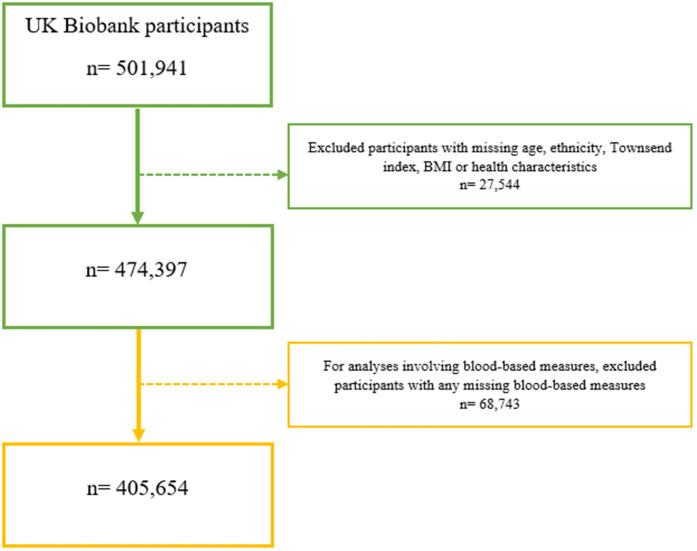
Flow chart for study population.

From the 501,941 participants included in the UKB study at baseline, we excluded 27,544 participants with missing covariate or health outcome data, yielding a sample of 474,397 participants (Fig 1). Overall, excluded participants (N = 5,815, 1.2%) had a poorer health profile; they were more likely to show concurrent health outcomes or to die during the follow-up (Table C in S1 File). Data on blood-based measures were missing for some participants, and so these analyses were based on 405,654 participants with fully available blood-based samples.

The characteristics of the 474,397 participants are shown in Table D in S1 File; the mean age (SD) was 56.5 years (±8.1), mean Townsend Deprivation Index was −1.4 (±3.1), and mean BMI was 27.4 (±4.8) kg/m^2^. Most participants were women (54.5%, *n* = 258,575).

### Comparison of prevalence using seven multimorbidity definitions

The prevalence of multimorbidity varied substantially depending on the operational definition used and sex (Table 1). For count approaches, the prevalence of multimorbidity was highest using the full list of 38 conditions and lowest using the reduced list of 12 conditions; the prevalence of multimorbidity in men (women) was 34.0% (35.3%) for *Count All: 2+*, 26.4% (29.1%) for *Count MostPrev: 2+*, and 31.8% (33.9%) for *Count Syst: 2+*. The prevalence for approaches using a threshold of 3+ conditions to define multimorbidity was lower than that using 2+. The mean count of conditions and mean age of participants were greater using the 3 + definition. For instance, while the mean age and condition count for multimorbid men in *Count All: 2+* were 59.5 years and 3.1 conditions, men who were multimorbid using the *Count All: 3 +* definition were, on average, 60.3 years and had 4.0 conditions.

**Table 1:** Multimorbidity prevalence, count of chronic conditions, and age at baseline in men and women.

Multimorbidity measure^1^			Prevalence % (*n*)	Condition Count^2^ Mean (SD)	Age at Baseline Mean (SD)
**MEN (*N* = 215,822)**					
**Count** **All**	**2+**	No Multimorbidity	66.0 (142,460)	0.4 (0.5)	55.3 (8.2)
		Multimorbidity	34.0 (73,362)	3.1 (1.4)	59.5 (7.4)
	**3+**	No Multimorbidity	82.0 (176,898)	0.7 (0.8)	55.9 (8.2)
		Multimorbidity	18.0 (38,924)	4.0 (1.3)	60.3 (7.1)
**Count MostPrev**	**2+**	No Multimorbidity	73.6 (158,740)	0.4 (0.5)	55.7 (8.2)
		Multimorbidity	26.4 (57,082)	2.6 (0.9)	59.4 (7.4)
	**3+**	No Multimorbidity	89.0 (192,155)	0.7 (0.8)	56.3 (8.2)
		Multimorbidity	11.0 (23,667)	3.5 (0.8)	60.0 (7.2)
**Count** **Syst**	**2+**	No Multimorbidity	68.2 (147,241)	0.4 (0.5)	55.4 (8.2)
		Multimorbidity	31.8 (68,581)	2.7 (1.0)	59.5 (7.4)
	**3+**	No Multimorbidity	85.3 (184,139)	0.7 (0.8)	56.1 (8.2)
		Multimorbidity	14.7 (31,683)	3.6 (0.9)	60.3 (7.1)
**Clustering**		No Multimorbidity	66.0 (142,460)	0.4 (0.5)	55.3 (8.2)
		Mental Disorders	3.7 (7,994)	3.1 (1.3)	56.9 (7.9)
		Respiratory	4.9 (10,490)	3.2 (1.4)	58.6 (7.8)
		Digestive + Osteoporosis	4.1 (8,929)	3.5 (1.6)	59.7 (7.5)
		Painful Conditions + Cardiovascular and Metabolic disorders	10.5 (22,605)	2.8 (1.1)	59.1 (7.4)
		Hypertension + Other	8.2 (17,769)	2.5 (0.8)	61.6 (6.3)
		Liver + Alcohol + Dementia	1.2 (2,518)	4.2 (2.0)	57.6 (7.6)
		Circulatory	1.4 (3,057)	4.9 (2.0)	60.9 (6.7)
**WOMEN (*N* = 258,575)**					
**Count** **All**	**2+**	No Multimorbidity	64.7 (167,314)	0.4 (0.5)	55.3 (8.0)
		Multimorbidity	35.3 (91,261)	3.1 (1.4)	58.2 (7.6)
	**3+**	No Multimorbidity	80.9 (209,058)	0.7 (0.8)	55.8 (8.0)
		Multimorbidity	19.1 (49,517)	4.0 (1.4)	58.7 (7.5)
**Count MostPrev**	**2+**	No Multimorbidity	70.9 (183,456)	0.4 (0.5)	55.5 (8.0)
		Multimorbidity	29.1 (75,119)	2.8 (1.0)	58.3 (7.6)
	**3+**	No Multimorbidity	86.6 (224,009)	0.7 (0.8)	56.0 (8.0)
		Multimorbidity	13.4 (34,566)	3.7 (1.0)	58.7 (7.5)
**Count** **Syst**	**2+**	No Multimorbidity	66.1 (170,924)	0.4 (0.5)	55.4 (8.0)
		Multimorbidity	33.9 (87,651)	2.8 (1.0)	58.2 (7.6)
	**3+**	No Multimorbidity	83.4 (215,715)	0.8 (0.8)	55.9 (8.0)
		Multimorbidity	16.6 (42,860)	3.7 (0.9)	58.7 (7.5)
**Clustering**		No Multimorbidity	64.7 (167,314)	0.4 (0.5)	55.3 (8.0)
		Mental Disorders	1.1 (2,768)	3.7 (1.9)	54.6 (7.9)
		Respiratory	1.0 (2,680)	4.2 (2.0)	60.0 (6.9)
		Digestive	9.2 (23,868)	3.2 (1.5)	58.7 (7.4)
		Painful Conditions + Other	13.7 (35,516)	2.7 (1.0)	58.1 (7.6)
		Asthma + Other	5.3 (13,721)	3.1 (1.4)	57.0 (8.0)
		Connective + Osteoporosis + Neurological	4.9 (12,708)	3.5 (1.7)	59.4 (7.3)

^1^Multimorbidity definitions are based on counting conditions or clustering. Count All, Count MostPrev, and Count Syst, count the number of all conditions, 12 most prevalent conditions, or number of body systems involved by conditions, respectively. For count-based approaches, 2+ or 3+ thresholds were used to categorize participants as multimorbid or not. Cluster groups were identified among those multimorbid based on the Count All: 2+ Measure.

^2^The condition count is reported using the respective list of conditions for each method.

**Abbreviations**: Count MostPrev, 12 most prevalent conditions in the list; Count Syst, count of body systems involved; 2+, multimorbidity defined using 2 or more conditions; 3+, multimorbidity defined using 3 or more conditions.

The clustering approach (S1 Methods: Cluster Analyses), which stratified participants identified as multimorbid using the *Count All: 2+* definition (*n* = 73,362 men and 91,261 women) based on the combination of their conditions, identified seven multimorbidity clusters in men and six in women. Based on the prevalence of conditions, observed/expected ratio, and exclusivity of disease in each identified cluster, in men the clusters and their respective prevalence were: liver + alcohol + dementia (1.2%), circulatory (1.4%), mental disorders (3.7%), digestive + osteoporosis (4.1%), respiratory (4.9%), hypertension + other (8.2%), and painful conditions + cardiovascular and metabolic disorders (10.5%). In women, the clusters were: respiratory (1.0%), mental disorders (1.1%), connective + osteoporosis + neurological (4.9%), asthma + other (5.3%), digestive (9.2%), and painful conditions + other (13.7%). The most frequent combinations of disease for each cluster are shown in S1 Methods: Cluster Analyses. There was greater variation in mean ages and condition counts using the clustering approach, compared to multimorbidity definitions based on counts. For those considered multimorbid with 2 or more diseases from the full list, the mean number of conditions varied between 2.5 (hypertension + other) and 4.9 (circulatory) in men and between 2.7 (painful conditions + other) and 4.2 (respiratory) in women. Men and women in the mental disorder clusters were younger compared to other multimorbidity clusters (mean age in men = 56.9, women = 54.6 years).

The most common concurrent health outcome was surgery (62.0%), and least common was frailty (4.0%) (Table D in S1 File). 4.7% of the study population had died at the 10-year time horizon (Table D in S1 File). Further details of concurrent health outcomes and mortality as a function of multimorbidity measures are shown in Table E for men and Table F for women in S1 File.

For all definitions, prevalence increases with age (Table G in S1 File). Between the ages of 38–53 and 61–73, it rises from 20.2% to 46.2% for men and from 25.9% to 44.4% for women, according to the *Count All: 2+* definition. The largest relative increase between the groups is observed in the circulatory cluster for men and in the connective + osteoporosis + neurological cluster for women. The only exception concerns the ‘Mental disorders’ clusters, in both men and women, where prevalence remains relatively stable.

### Cross-sectional association of multimorbidity measures with health outcomes

All multimorbidity definitions were associated with health outcomes measured concurrently in men and women, as displayed in Table 2 (Unadjusted results Table H in S1 File). The count-based approaches generally had the largest ORs for definitions using the full list of 38 conditions, while the smallest ORs were associated with definitions based on the 12 most prevalent conditions. Definitions using a threshold of three conditions to define multimorbidity yielded stronger associations compared to those using a threshold of two.

**Table 2:** Cross-sectional associations of multimorbidity measures with health outcomes in men and women.

Multimorbidity measures^1^		Prevalence %	Health outcomes (Odds Ratio (95% Confidence Interval)^2^					
			Polypharmacy	Poor self-rated health	Frailty	Falls	Surgery	Chronic Pain
**MEN (*N* = 215,822), mean (SD) age: 56.7 (8.2) years**								
**Count** **All**	**2+**	34.0	6.02 (5.86, 6.18)	5.43 (5.18, 5.69)	3.74 (3.54, 3.95)	1.45 (1.41, 1.49)	4.21 (4.12, 4.30)	1.68 (1.65, 1.71)
	**3+**	18.0	6.24 (6.07, 6.40)	5.72 (5.47, 5.97)	4.17 (3.96, 4.39)	1.57 (1.53, 1.62)	5.21 (5.04, 5.37)	1.82 (1.78, 1.86)
**Count MostPrev**	**2+**	26.4	4.99 (4.86, 5.11)	4.27 (4.09, 4.46)	3.06 (2.91, 3.23)	1.41 (1.38, 1.45)	3.61 (3.53, 3.70)	1.72 (1.69, 1.76)
	**3+**	11.0	5.57 (5.40, 5.74)	4.64 (4.44, 4.86)	3.51 (3.33, 3.71)	1.56 (1.51, 1.62)	4.66 (4.49, 4.85)	1.96 (1.91, 2.02)
**Count** **Syst**	**2+**	31.8	5.01 (4.88, 5.14)	4.92 (4.70, 5.15)	3.53 (3.35, 3.73)	1.46 (1.42, 1.50)	3.81 (3.73, 3.90)	1.70 (1.67, 1.73)
	**3+**	14.7	5.06 (4.92, 5.20)	4.98 (4.76, 5.20)	3.82 (3.62, 4.02)	1.61 (1.56, 1.65)	4.43 (4.28, 4.58)	1.85 (1.80, 1.90)
**Clustering**	Mental Disorders	3.7	5.35 (5.07, 5.65)	6.41 (5.93, 6.92)	4.48 (4.07, 4.92)	1.81 (1.71, 1.91)	3.24 (3.07, 3.41)	1.81 (1.73, 1.90)
	Respiratory	4.9	7.98 (7.62, 8.36)	6.96 (6.48, 7.46)	4.54 (4.17, 4.95)	1.50 (1.42, 1.57)	3.38 (3.22, 3.54)	1.76 (1.69, 1.83)
	Digestive + Osteoporosis	4.1	4.97 (4.72, 5.23)	6.41 (5.92, 6.93)	4.04 (3.66, 4.45)	1.40 (1.32, 1.47)	10.13 (9.42, 10.90)	2.06 (1.97, 2.15)
	Painful Conditions + Cardiovascular and Metabolic disorders	10.5	5.49 (5.30, 5.69)	3.89 (3.65, 4.14)	3.25 (3.02, 3.49)	1.37 (1.32, 1.42)	2.81 (2.72, 2.90)	1.63 (1.59, 1.68)
	Hypertension + Other	8.2	5.29 (5.09, 5.50)	3.76 (3.50, 4.05)	2.34 (2.14, 2.55)	1.27 (1.22, 1.33)	6.04 (5.78, 6.31)	1.42 (1.37, 1.47)
	Liver + Alcohol + Dementia	1.2	7.74 (7.09, 8.43)	10.10 (9.06, 11.25)	6.48 (5.68, 7.37)	1.98 (1.80, 2.16)	7.22 (6.41, 8.16)	1.91 (1.77, 2.07)
	Circulatory	1.4	16.58 (15.31, 17.97)	11.83 (10.68, 13.08)	7.01 (6.20, 7.91)	1.72 (1.58, 1.87)	16.11 (13.86, 18.86)	1.75 (1.63, 1.89)
**WOMEN (*N* = 258,575), mean (SD) age: 56.3 (8.0) years**								
**Count** **All**	**2+**	35.3	4.33 (4.23, 4.43)	5.41 (5.15, 5.68)	3.20 (3.07, 3.33)	1.36 (1.33, 1.38)	3.44 (3.38, 3.51)	1.86 (1.82, 1.89)
	**3+**	19.1	4.69 (4.59, 4.80)	5.65 (5.41, 5.91)	3.41 (3.28, 3.55)	1.45 (1.42, 1.48)	4.10 (3.99, 4.22)	2.06 (2.02, 2.10)
**Count MostPrev**	**2+**	29.1	3.82 (3.74, 3.90)	4.28 (4.09, 4.48)	2.78 (2.67, 2.89)	1.34 (1.31, 1.37)	3.16 (3.09, 3.23)	1.83 (1.80, 1.86)
	**3+**	13.4	4.30 (4.19, 4.41)	4.69 (4.48, 4.90)	2.99 (2.87, 3.12)	1.44 (1.41, 1.48)	3.93 (3.81, 4.06)	2.09 (2.04, 2.14)
**Count** **Syst**	**2+**	33.9	4.16 (4.07, 4.25)	5.11 (4.87, 5.36)	3.11 (2.99, 3.24)	1.36 (1.33, 1.38)	3.32 (3.25, 3.39)	1.85 (1.82, 1.88)
	**3+**	16.6	4.40 (4.30, 4.51)	5.19 (4.97, 5.43)	3.25 (3.12, 3.38)	1.44 (1.41, 1.47)	3.84 (3.73, 3.95)	2.05 (2.01, 2.10)
**Clustering**	Mental Disorders	1.1	4.90 (4.51, 5.33)	8.70 (7.71, 9.79)	4.64 (4.11, 5.23)	1.75 (1.61, 1.90)	3.44 (3.12, 3.81)	1.73 (1.60, 1.87)
	Respiratory	1.0	8.16 (7.53, 8.85)	16.25 (14.53, 18.15)	6.78 (6.06, 7.58)	1.47 (1.36, 1.60)	4.44 (3.99, 4.95)	2.16 (2.00, 2.34)
	Digestive	9.2	4.10 (3.97, 4.24)	5.12 (4.79, 5.47)	2.93 (2.77, 3.11)	1.29 (1.25, 1.33)	6.76 (6.47, 7.06)	2.16 (2.10, 2.23)
	Painful Conditions + Other	13.7	3.03 (2.94, 3.12)	3.31 (3.10, 3.53)	2.27 (2.15, 2.40)	1.22 (1.19, 1.25)	2.31 (2.25, 2.37)	1.56 (1.53, 1.60)
	Asthma + Other	5.3	6.19 (5.95, 6.43)	5.06 (4.68, 5.45)	3.04 (2.84, 3.25)	1.44 (1.38, 1.49)	3.20 (3.07, 3.35)	1.95 (1.88, 2.02)
	Connective + Osteoporosis + Neurological	4.9	6.71 (6.45, 6.99)	10.91 (10.17, 11.70)	6.09 (5.72, 6.49)	1.71 (1.65, 1.78)	4.73 (4.50, 4.98)	2.12 (2.04, 2.20)

^1^Multimorbidity definitions are based on counting conditions or clustering. Count All, Count MostPrev, and Count Syst count the number of all conditions, 12 most prevalent conditions or number of body systems affected, respectively. Cluster groups were identified among those multimorbid based on the Count All: 2+ Measure.

^2^Logistic regression adjusted for age, Townsend deprivation index, ethnicity, and body mass index. Odds Ratios represent the odds of a health outcome among those with the corresponding multimorbid status, as compared to the reference group of those identified as “not multimorbid”.

**Abbreviations**: Count MostPrev, 12 most prevalent conditions in the list; Count Syst, count of body systems involved; 2+, multimorbidity defined using 2 or more conditions; 3+, multimorbidity defined using 3 or more conditions.

In men and women, the digestive cluster had comparatively large ORs of surgery and chronic pain. Among the other concurrent health outcomes considered, the largest OR estimates in men were generally linked to the circulatory and liver + alcohol + dementia clusters, corresponding to the smallest-prevalence clusters. In women, the respiratory and connective + osteoporosis + neurological clusters consistently had the largest ORs for associations with health outcomes.

### Cross-sectional association of multimorbidity measures with blood-based measures

**Fig 2:**
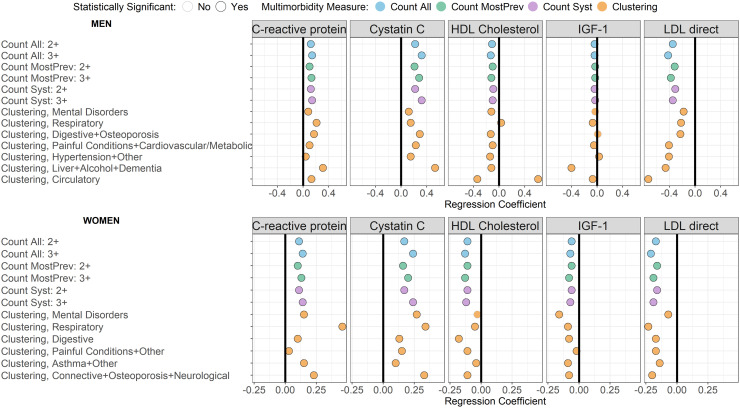
Cross-sectional association (regression coefficient) between multimorbidity measures and blood-based measures in men (*N* = 186,873) and women (*N* = 218,781). **Abbreviations**: Count MostPrev = 12 most prevalent conditions in the list; Count Syst = count of body-systems involved; 2+ = multimorbidity defined using 2 or more conditions; 3+ = multimorbidity defined using 3 or more conditions; HDL, High-Density Lipoprotein; IGF, Insulin-like Growth Factor; LDL, low-density lipoprotein. Multimorbidity definitions are based on counting conditions or clustering. Count All, Count MostPrev, and Count Syst, count the number of all conditions, 12 most prevalent conditions, or number of body systems involved by conditions, respectively. For count-based approaches, 2+ or 3+ thresholds were used to categorize participants as multimorbid or not. Cluster groups were identified among those multimorbid based on the Count All: 2+ Measure. Analyses were undertaken using linear regression adjusted for age, Townsend deprivation index, ethnicity, and body mass index. Estimates represent the expected change in each blood-based measure among those with the corresponding multimorbid status, as compared to the reference group of those identified as “not multimorbid”. Blood-based measures were standardized to have mean 0 and standard deviation equal to 1, separately in men and women. Analyses were completed among 405,654 participants with fully observed blood-based measures.

Blood-based measures show objective biological profiles that differ between people with multiple health conditions and those without. The mean values of standardized CRP, Cystatin C, HDL cholesterol, IGF-1, and LDL cholesterol are shown in Table I in S1 File and estimated coefficients for the association between multimorbidity measures and these measures in Fig 2, along with Table J in S1 File, in the sub-cohort of 405,654 participants with complete blood-based measures. In both men and women, regardless of the multimorbidity definition considered, multimorbidity was associated with higher levels of CRP and Cystatin C, and lower levels of HDL cholesterol, IGF-1, and LDL cholesterol, relative to those not identified as multimorbid. The magnitude of these associations was consistently larger when using a threshold of three conditions compared to two.

The estimated coefficients for some clusters were notably larger than those observed for count-based definitions. For example, in men, while the estimated impact of multimorbidity on Cystatin C levels was 0.22 (0.21, 0.23) SD using *Count All: 2+*, the corresponding estimate for the circulatory cluster was 0.61 (0.58, 0.65) SD. In women, the estimated coefficient for multimorbidity on CRP levels was 0.11 (0.10, 0.12) SD using *Count All: 2+*, though those in the respiratory cluster had 0.46 (0.42, 0.50) SD higher levels of this protein compared to non-multimorbid women.

### Association of multimorbidity with mortality

**Fig 3:**
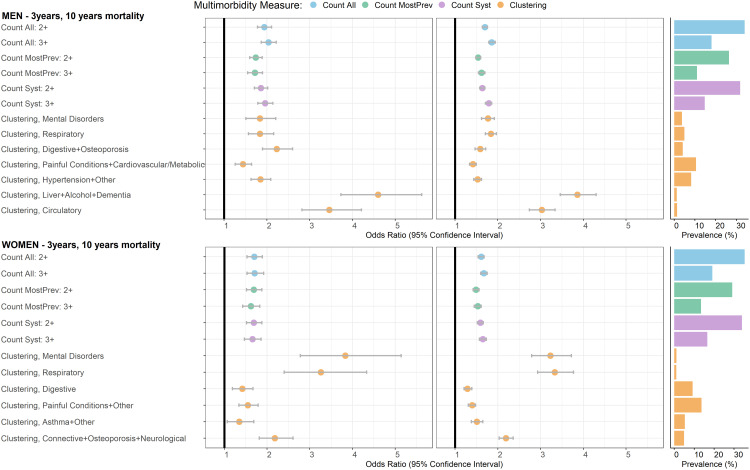
Associations (Odds Ratio (95% Confidence Interval)) between multimorbidity measures at baseline and mortality (at 3 or 10 years of follow-up) in men and women. **Abbreviations**: Count MostPrev, 12 most prevalent conditions in the list; Count Syst, count of body systems involved; 2+, multimorbidity defined using 2 or more conditions; 3+, multimorbidity defined using 3 or more conditions. Multimorbidity definitions are based on counting conditions or clustering. Count All, Count MostPrev, and Count Syst, count the number of all conditions, 12 most prevalent conditions, or number of body systems involved by conditions, respectively. For count-based approaches, 2+ or 3+ thresholds were used to categorize participants as multimorbid or not. Cluster groups were identified among those multimorbid based on the Count All: 2+ Measure. Analyses undertaken using logistic regression models adjusted for age, Townsend deprivation index, ethnicity, and body mass index. Odds Ratios represent the odds of dying among those with the corresponding multimorbid status, as compared to the reference group of those identified as “not multimorbid”.

The risk of mortality at 3- and 10-years after initial assessment is displayed in Fig 3 (corresponding results in Table K, unadjusted results Table L, in S1 File). All ORs were greater than 1 and statistically significant, indicating a higher risk of mortality in those with multimorbidity compared to non-multimorbid participants. The choice of threshold (two or three) and condition list both had minimal impact on the estimated association between multimorbidity and mortality. The magnitude of the ORs was highest using the full list of conditions and lowest using the MostPrev list, with corresponding prevalence’s following the same pattern. In men, the OR (95% CI) for 10-year mortality was 1.70 (1.64, 1.76) using *Count All: 2+*, 1.64 (1.58, 1.70) using *Count Syst: 2+*, and 1.54 (1.49, 1.60) using *Count MostPrev: 2+*. A similar pattern was observed in women, with ORs of 1.67 (1.60, 1.75) for *Count All: 3+*, 1.65 (1.57, 1.73) for *Count Syst: 3+*, and 1.53 (1.45, 1.61) for *Count MostPrev: 3+*.

The cluster-based approach showed greater variability in estimated ORs compared to count-based definitions. For men, ORs ranged from 1.44 [1.26; 1.64] to 4.60 [3.73; 5.62] for 3-year mortality and from 1.42 [1.34; 1.50] to 3.86 [3.46; 4.30] for 10-year mortality. For women, the ORs for 3-year mortality ranged from 1.35 [1.07; 1.69] to 3.83 [2.78; 5.14] and from 1.29 [1.21; 1.39] to 3.33 [2.93; 3.77] for 10-year mortality. Clusters with the lowest prevalence generally corresponded to combinations of conditions associated with higher mortality risks. In men, those in the liver + alcohol + dementia and circulatory clusters had ORs (95% CI) of 3.86 (3.46, 4.30) and 3.03 (2.74, 3.34), respectively, for 10-year mortality, compared to non-multimorbid men. These ORs were much higher than those for men in the painful conditions + cardiovascular and metabolic disorders cluster, that had an OR (95% CI) of 1.42 (1.34, 1.50) and was the most prevalent cluster. In women, the mental disorders and respiratory clusters, both of which had the lowest prevalence, were associated with the highest ORs for mortality.

Secondary analysis, using a Cox model on a time-to -event data framework, shows similar conclusions (Table M in S1 File).

## Discussion

This study examined how commonly used operational definitions of multimorbidity, including count-based definitions and a cluster-based approach, compare when applied within the same population, addressing the limited evidence on how these methodological choices influence prevalence estimates and associations with health outcomes and mortality. We identified substantial variations in multimorbidity prevalence, ranging from 1.0% for the smallest multimorbidity cluster to 35.3% using the full list of 38 chronic conditions and the threshold of two or more conditions. Lists with fewer conditions resulted in lower prevalence estimates. We found that although the cut-off used to define multimorbidity had a substantial impact on estimated prevalence, its influence on associations with concurrent health outcomes, blood-based measures, and mortality risk was more limited. In particular, mortality associations remained relatively stable across count-based definitions despite large differences in prevalence estimates. This suggests that while the number and type of conditions included in multimorbidity definitions strongly affect estimates of disease burden, their influence on the association between multimorbidity and mortality may be more modest within this population. Another important aspect of our study was the comparison of these count-based definitions with a clustering approach, which aims to characterize patterns of co-occurring conditions and identify subgroups of individuals with similar disease profiles [21,22]. Compared with count-based approaches, clustering produced greater variation in both prevalence and association estimates. Notably, some less prevalent clusters showed particularly strong associations with poor health status, including the liver + alcohol + dementia and circulatory clusters in men, and the respiratory and mental disorders clusters in women.

Previous studies have similarly identified that as the number of conditions listed in multimorbidity definitions increases, the prevalence of multimorbidity rises [11,17]. We found an approximately 5.5%–8.0% difference in prevalence using the full list of 38 conditions compared to using the reduced list of 12 proposed by Fortin [16]. Harrison and colleagues [17] found a slightly larger difference using the 2+ or 3 condition threshold; the prevalence of multimorbidity was 37.0% and 23.4%, respectively, using Fortin’s reduced list [16] and 47.4% and 33.8%, respectively, using their list of chronic conditions. A study using Canadian data [11] similarly found higher prevalence using 60 conditions compared to a reduced list of 20. We identified less than a 3.5% difference in prevalence estimated using the granular, full list (38 chronic conditions) and that estimated using ICD chapter counts. The difference was larger when a threshold of two or more conditions was used to define multimorbidity, comparable to what Harrison and colleagues [17] identified when comparing treating every chronic condition as a separate disease entity versus counting the number of chapters affected by at least one condition.

Despite emerging consensus on a cut-off of three or more conditions being of greater clinical use as it captures a more vulnerable population, two or more conditions remain widely used in research studies [8,11,17]. The decline in prevalence of multimorbidity when increasing the threshold from two to three was most pronounced in definitions using counts of affected ICD-10 chapters. Using a list of 40 conditions and UK Biobank study, Hanlon and colleagues [37] found increasing the cut-off for number of conditions from two to three reduced the prevalence of multimorbidity by 13.8%; in our study on 38 conditions, the corresponding numbers were 16.0% in men and 16.2% in women. Results from another study confirm this pattern [17].

The clustering analysis provided a complementary perspective by describing heterogeneity among participants with multiple chronic conditions. Clusters differed substantially in both prevalence and health profiles, reflecting the underlying combinations of diseases that characterize each subgroup. In particular, clusters with relatively low prevalence were often composed of more severe or complex combinations of conditions and therefore showed stronger associations with several health outcomes and mortality. These differences likely reflect the composition and severity of conditions within clusters rather than the predictive performance of the clustering method itself. In men, the liver + alcohol + dementia and circulatory clusters were not very prevalent (1.2% and 1.4%, respectively), but the health profiles, determined using the six outcomes and five blood-based measures, were poorer and risk of mortality higher than in other clusters. In women, the mental disorders and respiratory clusters, with prevalence of 1.1% and 1.0%, respectively, had higher mortality risk than the other clusters. The respiratory cluster in women also had a poorer health profile, including higher CRP levels. A recent study using UK Biobank data, conducted using a different condition list and clustering approach, identified similar clusters to ours and found the mental health and respiratory clusters for women and the cardiometabolic condition cluster for men to be associated with higher mortality risk [20]. Steell and colleagues noted the presence of pulmonary and cardiometabolic clusters in UK Biobank study and reported lower health-related quality of life in these clusters, compared to the not multimorbid group, to varying extents [7]. The insights from these previous studies, along with the results provided from this study, highlight the existence of non-random multimorbidity clusters and the higher risk of poorer health outcomes in certain clusters.

Our results have important implications for how multimorbidity ought to be defined and measured in future studies. This choice is likely to depend on the context and whether the goal is epidemiological description, identification of risk factor, management of patients at greater risk of adverse events, or evaluation of interventions. Key considerations include the need for comparability across studies, the ability to identify profiles at higher risk of adverse outcomes, and, particularly for interventions, the size of the groups that ought to be targeted for individual or large-scale interventions. Use of a standardized list, such as the one proposed by Ho and colleagues [14], is important for replication but faces challenges related to access to data and harmonization of relevant coding schemes for chronic conditions. Our findings suggest that the choice of conditions in a list, used by both the count-based and clustering approaches, has an impact on certain health correlates of multimorbidity. A Delphi consensus recommended defining multimorbidity using a simple condition count with a threshold of two or more conditions and no *a priori* limitation on the list of conditions, but it also suggested that severe multimorbidity ought to be measured using a threshold of three and above [14]. Our findings suggest that use of a higher threshold on the number of chronic conditions does not necessarily linked to greater mortality risks, and that cluster-based approaches provide a complementary perspective by describing patterns of co-occurring conditions within multimorbid populations. As compared to the count-based approach, clustering identifies smaller multimorbidity groups that are each less heterogenous in terms of conditions. However, labeling and defining the clusters remains challenging. It should be noted that there is no consensus on the methodology for defining multimorbidity clusters. We followed the approach of Violan and colleagues [34], based on k-means clustering, a widely used and statistically robust method. This approach was also favored because it allows evaluation of cluster composition through multiple indicators, including model-agnostic ones, which enhances comparability across studies.

A primary strength of our study is the use of count and clustering methods to define multimorbidity and its impact on health in the same dataset. To allow for replication and facilitate comparisons with existing research, we used a previously developed clustering procedure [34] and a list of conditions employed in previous UK Biobank multimorbidity studies [25]. While use of the UK Biobank data for these analyses provided access to a large sample size and a rich, comprehensive set of health data, previous studies have shown that the cohort study is not representative of the UK population [38]. We also note the exclusion of participants whose health appears to be poorer, although they represent a small proportion (1.2%). Findings may not be generalizable to more vulnerable populations, such as institutionalized adults. In addition, some of the variables used in this study are based on self-reported data, which may be subject to bias [39]. A further limitation is that we did not consider the impact of some of the other previously identified differences in multimorbidity measurement, such as varying the lookback period or considering the use of self-reported versus electronic health records to define chronic conditions. Modifying these factors was outside the scope of our study, as we wished to focus on the most commonly reported differences in multimorbidity measurement across previous studies. Finally, the health outcomes and blood-based measures could only be examined cross-sectionally and no conclusions on the causal direction of associations can be drawn from these analyses. Specific longitudinal studies could clarify this point and also indicate whether the commonly acknowledged risk factors for multimorbidity actually depend on the definition used to characterize it.

Given the expected increase in multimorbidity prevalence linked to global aging and its impact on health, our results emphasize the need for consensus on definition and measurement of multimorbidity. The choice of a definition impacts key epidemiological indicators and depends on both the research or clinical context, as well as the availability of data. We encourage researchers to carefully consider and justify their choice of definition when designing studies or interventions, as without such transparency, the potential for translational research on multimorbidity may remain limited.

## Supporting information

S1 File **Including: Table A. List of chronic conditions and corresponding ICD-10 codes used to define multimorbidity indicators.** Abbreviation: ICD: International Classification of Diseases; ICD-10 codes for chronic conditions were based on the list of codes used to define these conditions in Ronaldson and colleagues [25]. **Table B. Chronic conditions used in multimorbidity definitions.** Abbreviation: ICD: International Classification of Diseases. **Table C. Descriptive data (% (*N*) or Mean (SD)) on covariates and concurrent health outcomes in included(*N* = 474,397) and excluded (*N* = 5,815) participants.** Abbreviation: SD: standard deviation; ^1^Mean (Standard Deviation) reported for these measures. **Table D. Descriptive data (% (*N*) or Mean (SD)) on covariates and concurrent health outcomes.** ^1^Mean (Standard Deviation) reported for these measures. **Table E. Descriptive data (% (***N***) or Mean (SD)) on concurrent health outcomes and mortality as a function of multimorbidity measures in MEN. Abbreviations**: Count MostPrev, 12 most prevalent conditions in the list; Count Syst, count of body systems involved; 2+, multimorbidity defined using 2 or more conditions; 3+, multimorbidity defined using 3 or more conditions. ^1^Multimorbidity definitions are based on counting conditions or clustering. Count All, Count MostPrev, and Count Syst, count the number of all conditions, 12 most prevalent conditions, or number of body systems involved by conditions, respectively. For count-based approaches, 2+ or 3+ thresholds were used to categorize participants as multimorbid or not. Cluster groups were identified among those multimorbid based on the Count All 2+ Measure. ^2^Mean (SD) reported for these metrics. ^3^The condition count is reported using the respective list for each method. **Table F. Descriptive data (% (*N*) or Mean (SD)) on concurrent health outcomes and mortality as a function of multimorbidity measures in WOMEN. Abbreviations**: Count MostPrev, 12 most prevalent conditions in the list; Count Syst, count of body systems involved; 2+, multimorbidity defined using 2 or more conditions; 3+, multimorbidity defined using 3 or more conditions. ^1^Multimorbidity definitions are based on counting conditions or clustering. Count All, Count MostPrev, and Count Syst, count the number of all conditions, 12 most prevalent conditions, or number of body systems involved by conditions, respectively. For count-based approaches, 2+ or 3+ thresholds were used to categorize participants as multimorbid or not. Cluster groups were identified among those multimorbid based on the Count All 2+ Measure. ^2^Mean (SD) reported for these metrics. ^3^The condition count is reported using the respective list for each method. **Table G. Multimorbidity prevalence, overall and by age groups, defined using terciles, in men and women. Abbreviations**: Count MostPrev, 12 most prevalent conditions in the list; Count Syst, count of body systems involved; 2+ , multimorbidity defined using 2 or more conditions; 3+ , multimorbidity defined using 3 or more conditions. ^1^Multimorbidity definitions are based on counting conditions or clustering. Count All, Count MostPrev, and Count Syst, count the number of all conditions, 12 most prevalent conditions, or number of body systems involved by conditions, respectively. For count-based approaches, 2+ or 3+ thresholds were used to categorize participants as multimorbid or not. Cluster groups were identified among those multimorbid based on the Count All: 2+ Measure. **Table H: Cross-sectional associations of multimorbidity measures with health outcomes in men and women (unadjusted results). Abbreviations**: Count MostPrev, 12 most prevalent conditions in the list; Count Syst, count of body systems involved; 2+, multimorbidity defined using 2 or more conditions; 3+, multimorbidity defined using 3 or more conditions. ^**1**^Multimorbidity definitions are based on counting conditions or clustering. Count All, Count MostPrev, and Count Syst count the number of all conditions, 12 most prevalent conditions or number of body systems affected, respectively. Cluster groups were identified among those multimorbid based on the Count All: 2+ Measure. ^**2**^Logistic regression adjusted for age, Townsend deprivation index, ethnicity, and body mass index. Odds Ratios represent the odds of a health outcome among those with the corresponding multimorbid status, as compared to the reference group of those identified as “not multimorbid”. **Table I. Standardized mean value of blood-based measures as a function of multimorbidity measures. Abbreviations**: Count MostPrev, 12 most prevalent conditions in the list; Count Syst, count of body systems involved; 2+, multimorbidity defined using 2 or more conditions; 3+, multimorbidity defined using 3 or more conditions; HDL, High-Density Lipoprotein; IGF, Insulin-like Growth Factor; LDL, low-density lipoprotein. ^1^Multimorbidity definitions are based on counting conditions or clustering. Count All, Count MostPrev, and Count Syst, count the number of all conditions, 12 most prevalent conditions, or number of body systems involved by conditions, respectively. For count-based approaches, 2+ or 3+ thresholds were used to categorize participants as multimorbid or not. Cluster groups were identified among those multimorbid based on the Count All 2+ Measure. ^2^These analyses were completed among all 405,654 participants (# Men = 186,873, # Women = 218,781) with fully observed biomarker data. ^3^Blood-based measures were standardized to have mean 0 and standard deviation equal to 1, separately in men and women. **Table J. Cross-sectional association of multimorbidity measures with blood-based measures. Abbreviations**: Count MostPrev, 12 most prevalent conditions in the list; Count Syst, count of body systems involved; 2+, multimorbidity defined using 2 or more conditions; 3+, multimorbidity defined using 3 or more conditions; HDL, High-Density Lipoprotein; IGF, Insulin-like Growth Factor; LDL, low-density lipoprotein. ^1^Multimorbidity definitions are based on counting conditions or clustering. Count All, Count MostPrev, and Count Syst, count the number of all conditions, 12 most prevalent conditions, or number of body systems involved by conditions, respectively. For count-based approaches, 2+ or 3+ thresholds were used to categorize participants as multimorbid or not. Cluster groups were identified among those multimorbid based on the Count All 2+ Measure. ^2^Analyses based on 405,654 participants (# Men = 186,873, # Women = 218,781) with complete data. ^3^Blood-based measures were standardized to have mean 0 and standard deviation equal to 1, separately in men and women. ^4^Associations were computed using linear regression analysis adjusted for age, Townsend deprivation index, ethnicity, and body mass index. Estimates represent the expected change in each blood-based measure among those with the corresponding multimorbid status, as compared to the reference group of those identified as “not multimorbid”. **Table K. Association of multimorbidity measures and mortality at 3 and 10 years of follow-up. Abbreviations**: Count MostPrev, 12 most prevalent conditions in the list; Count Syst, count of body systems involved; 2+, multimorbidity defined using 2 or more conditions; 3+, multimorbidity defined using 3 or more conditions. ^1^Multimorbidity definitions are based on counting conditions or clustering. Count All, Count MostPrev, and Count Syst, count the number of all conditions, 12 most prevalent conditions, or number of body systems involved by conditions, respectively. For count-based approaches, 2+ or 3+ thresholds were used to categorize participants as multimorbid or not. Cluster groups were identified among those multimorbid based on the Count All 2+ Measure. ^2^Association between multimorbidity and death at 3 or 10 years were computed using logistic regression adjusted for age, Townsend deprivation index, ethnicity, and body mass index. Odds Ratios represent the odds of dying among those with the corresponding multimorbid status, as compared to the reference group of those identified as “not multimorbid”. **Table L: Association of multimorbidity measures and mortality at 3 and 10 years of follow-up (unadjusted results). Abbreviations**: Count MostPrev, 12 most prevalent conditions in the list; Count Syst, count of body systems involved; 2+, multimorbidity defined using 2 or more conditions; 3+, multimorbidity defined using 3 or more conditions. ^1^Multimorbidity definitions are based on counting conditions or clustering. Count All, Count MostPrev, and Count Syst, count the number of all conditions, 12 most prevalent conditions, or number of body systems involved by conditions, respectively. For count-based approaches, 2+ or 3+ thresholds were used to categorize participants as multimorbid or not. Cluster groups were identified among those multimorbid based on the Count All 2+ Measure. ^2^Association between multimorbidity and death at 3 or 10 years were computed using logistic regression adjusted for age, Townsend deprivation index, ethnicity, and body mass index. Odds Ratios represent the odds of dying among those with the corresponding multimorbid status, as compared to the reference group of those identified as “not multimorbid”. **Table M. Association of multimorbidity measures and mortality using a time-to-event framework. Abbreviations**: Count MostPrev, 12 most prevalent conditions in the list; Count Syst, count of body-systems involved; 2+, multimorbidity defined using 2 or more conditions; 3+, multimorbidity defined using 3 or more conditions; HR: Hazard Ratio; CI: Confidence Interval. ^1^Multimorbidity definitions are based on counting conditions or clustering. Count All, Count MostPrev, and Count Syst, count the number of all conditions, 12 most prevalent conditions, or number of body systems involved by conditions, respectively. For count-based approaches, 2+ or 3+ thresholds were used to categorize participants as multimorbid or not. Cluster groups were identified among those multimorbid based on the Count All 2+ Measure. ^2^Association between multimorbidity and death were computed using Cox models adjusted for age, Townsend deprivation index, ethnicity, and body mass index.
(DOCX)

S1 Checklist STROBE Checklist. Available at https://www.strobe-statement.org/checklists/.
(DOCX)

S1 Methods **Including: Method A:** methods used for cluster analyses. **Method B:** Men, step 1. Selecting the optimal number of clusters among 73,362 multimorbid men using various clustering metrics. **Method C:** Men, step 2. Labeling and summarizing the clusters. **Method D:** Table. Summary values at the optimal number of clusters in men. **Method E:** Figure. Prevalence, Observed/Expected Ratio, Exclusivity for the seven clusters in men. **Method F:** Figure. Ten most frequent combinations of conditions within each cluster in men. **Method G:** WOMEN, Step 1. Selecting the optimal number of clusters among 91,261 multimorbid women using various clustering metrics. **Method H:** WOMEN, Step 2. Labeling and summarizing the clusters. **Method I:** Table. Summary values at the optimal number of clusters in women. **Method J:** Figure. Prevalence, Observed/Expected Ratio, Exclusivity for the six clusters in women. **Method K:** Figure. Ten most frequent combinations of conditions within each cluster in women.
(DOCX)

## Abbreviations

BMI: body mass index
OR: odds ratio
STROBE: Strengthening the Reporting of Observational Studies in Epidemiology
UKB: UK Biobank
