# Supplementary material for: Comparison of count-based and clustering definitions of multimorbidity and their association with prevalence of multimorbidity, health profiles, and mortality: A cohort study of UK Biobank participants
Source: PLoS Med. 2026 Jun 12;23(6):e1004914. doi: 10.1371/journal.pmed.1004914 (PMC13309015; doi:10.1371/journal.pmed.1004914)
Supplement: S1 File — Including: Table A. List of chronic conditions and corresponding ICD-10 codes used to define multimorbidity indicators. Abbreviation: ICD: International Classification of Diseases; ICD-10 codes for chronic conditions were based on the list of codes used to define these conditions in Ronaldson and colleagues [25]. Table B. Chronic conditions used in multimorbidity definitions. Abbreviation: ICD: International Classification of Diseases. Table C. Descriptive data (% (N) or Mean (SD)) on covariates and concurrent health outcomes in included(N = 474,397) and excluded (N = 5,815) participants. Abbreviation: SD: standard deviation; 1Mean (Standard Deviation) reported for these measures. Table D. Descriptive data (% (N) or Mean (SD)) on covariates and concurrent health outcomes. 1Mean (Standard Deviation) reported for these measures. Table E. Descriptive data (% (N) or Mean (SD)) on concurrent health outcomes and mortality as a function of multimorbidity measures in MEN. Abbreviations: Count MostPrev, 12 most prevalent conditions in the list; Count Syst, count of body systems involved; 2+, multimorbidity defined using 2 or more conditions; 3+, multimorbidity defined using 3 or more conditions. 1Multimorbidity definitions are based on counting conditions or clustering. Count All, Count MostPrev, and Count Syst, count the number of all conditions, 12 most prevalent conditions, or number of body systems involved by conditions, respectively. For count-based approaches, 2+ or 3+ thresholds were used to categorize participants as multimorbid or not. Cluster groups were identified among those multimorbid based on the Count All 2+ Measure. 2Mean (SD) reported for these metrics. 3The condition count is reported using the respective list for each method. Table F. Descriptive data (% (N) or Mean (SD)) on concurrent health outcomes and mortality as a function of multimorbidity measures in WOMEN. Abbreviations: Count MostPrev, 12 most prevalent conditions in the list; Count Syst, count [file pmed.1004914.s001.docx]

**Comparison of count-based and clustering definitions of multimorbidity and their association with prevalence of multimorbidity, health profiles, and mortality: A cohort study of UK Biobank participants**

Gabriella C SILVA, PhD^1^; Aurore FAYOSSE, MSc^1^; Louis JACOB, MD, PhD^1,2,3^; Séverine SABIA, PhD^1, 4^; Archana SINGH-MANOUX, PhD^1, 4^; Benjamin LANDRÉ, PhD*^1^

1 Université Paris Cité, Inserm U1153, CRESS, Epidemiology of Ageing and Neurodegenerative diseases, Paris, France

2 Department of Physical Medicine and Rehabilitation, Université Paris Cité, AP-HP, Lariboisière-Fernand Widal Hospital, Paris 75010, France

3 Research and Development Unit, Parc Sanitari Sant Joan de Déu, CIBERSAM, ISCIII, Dr. Antoni Pujadas, 42, Sant Boi de Llobregat, Barcelona 08030, Spain

4 Faculty of Brain Sciences, University College London, London, UK

*Address for correspondence

Université Paris Cité

Inserm U1153, Epidemiology of Ageing and Neurodegenerative diseases

10 Avenue de Verdun, 75010 Paris, France

Email: [benjamin.landre@inserm.fr](mailto:gabriella.silva@inserm.fr)

**Supplementary material**

**Table A. List of chronic conditions and corresponding ICD-10 codes used to define multimorbidity indicators**

| **Condition** | **ICD-10 code** | **ICD-10 description (from UK Biobank)** | **UK Biobank Field ID [Source]** | **UK Biobank Field ID [Date/Age]** |
| --- | --- | --- | --- | --- |
| Asthma | J45 | Asthma | 131495 | 131494 |
| Atrial fibrillation | I48  I49 | Atrial fibrillation and flutter  Other cardiac arrhythmias | 131351  131353 | 131350  131352 |
| Bronchiectasis | J47 | Bronchiectasis | 131499 | 131498 |
| Cancer | *This was derived using the age at cancer diagnosis from central registry* | | | 40008 |
| Chronic kidney disease | N17  N18  N19  N00  N01  N03  N04  N05  N08  I12  I13 | Acute renal failure  Chronic renal failure  Unspecified renal failure  Acute nephritic syndrome  Rapidly progressive nephritic syndrome  Chronic nephritic syndrome  Nephrotic syndrome  Unspecified nephritic syndrome  Glomerular disorders in diseases classified elsewhere  Hypertensive renal disease  Hypertensive heart and renal disease | 132031  132033  132035  131999  132001  132005  132007  132009  132015  131291  131293 | 132030  132032  132034  131998  132000  132004  132006  132008  132014  131290  131292 |
| Chronic obstructive pulmonary disorder (COPD) | J44  J43  J42  J41  J40 | Other COPD  Emphysema  Unspecified Chronic Bronchitis  Simple and mucopurulent chronic bronchitis  Bronchitis, not specified as acute or chronic | 131493  131491  131489  131487  131485 | 131492  131490  131488  131486  131484 |
| Chronic sinusitis | J32 | Chronic sinusitis | 131469 | 131468 |
| Connective tissue disorders | K90  M05  M06  M07  M08  M30  M31  M32  M33  M34  M35  M36  M60 | Intestinal malabsorption  Seropositive rheumatoid arthritis  Other rheumatoid arthritis  Psoriatic and enteropathic arthropathies  Juvenile arthritis  Polyarteritis nodosa and related conditions  Other necrotising vasculopathies  Systemic lupus erythematosus  Dermatopolymyositis  Systemic sclerosis  Other systemic involvement of connective tissue  Systemic disorders of connective tissue in diseases classified elsewhere  Myositis | 131689  131849  131851  131853  131855  131891  131893  131895  131897  131899  131901  131903  131931 | 131688  131848  131850  131852  131854  131890  131892  131894  131896  131898  131900  131902  131930 |
| Coronary heart disease | I20  I21  I22  I23  I24  I25 | Angina pectoris  Acute myocardial infarction  Subsequent myocardial infarction  Certain current complications following acute myocardial infarction  Other acute ischaemic heart diseases  Chronic ischaemic heart disease | 131297  131299  131301  131303  131305  131307 | 131296  131298  131300  131302  131304  131306 |
| Dementia | F01  F02  F03  G30  G31 | Vascular dementia  Dementia in other diseases classified elsewhere  Unspecified dementia  Alzheimer’s disease  Other degenerative diseases of nervous system, not elsewhere classified | 130839  130841  130843  131037  131039 | 130838  130840  130842  131036  131038 |
| Diabetes | E11  E10  E13  O24 | Non-insulin-dependent diabetes mellitus  Insulin-dependent diabetes mellitus  Other specified diabetes mellitus  Diabetes mellitus in pregnancy | 130709  130707  130713  132203 | 130708  130706  130712  132202 |
| Diverticular disease | K57 | Diverticular disease of intestine | 131637 | 131636 |
| Dyspepsia/ulcer | K21  K22  K25  K26  K27  K28  K29  K30 | Gastro-oesophageal reflux disease  Other diseases of oesophagus  Gastric ulcer  Duodenal ulcer  Peptic ulcer, site unspecified  Gastrojejunal ulcer  Gastritis and duodenitis  Dyspepsia | 131585  131587  131591  131593  131595  131597  131599  131601 | 131584  131586  131590  131592  131594  131596  131598  131600 |
| Endometriosis | N80 | Endometriosis | 132123 | 132122 |
| Epilepsy | G40 | Epilepsy | 131049 | 131048 |
| Glaucoma | H40  H42 | Glaucoma  Glaucoma in diseases classified elsewhere | 131187  131189 | 131186  131188 |
| Heart failure | I46  I50 | Cardiac arrest  Heart failure | 131347  131355 | 131346  131354 |
| Hepatitis | B15  B16  B17  B18  B19 | Acute Hepatitis A  Acute Hepatitis B  Other acute viral hepatitis  Chronic viral hepatitis  Unspecified viral hepatitis | 130195  130197  130199  130201  130203 | 130194  130196  130198  130200  130202 |
| Hypertension | I10  I11  I12  I13  I15 | Essential (primary) hypertension  Hypertensive heart disease  Hypertensive renal disease  Hypertensive heart and renal disease  Secondary hypertension | 131287  131289  131291  131293  131295 | 131286  131288  131290  131292  131294 |
| Inflammatory bowel disease | K50  K51  K52 | Crohn’s disease [regional enteritis]  Ulcerative colitis  Other non-infective gastro-enteritis and colitis | 131627  131629  131631 | 131626  131628  131630 |
| Irritable bowel syndrome | K58 | Irritable bowel syndrome | 131639 | 131638 |
| Liver disease | K70  K71  K72  K73  K74  K75  K76  K77 | Alcoholic liver disease  Toxic liver disease  Hepatic failure, not elsewhere classified  Chronic hepatitis, not elsewhere classified  Fibrosis and cirrhosis of liver  Other inflammatory liver diseases  Other diseases of liver  Liver disorders in diseases classified elsewhere | 131659  131661  131663  131665  131667  131669  131671  131673 | 131658  131660  131662  131664  131666  131668  131670  131672 |
| Migraine | G43 | Migraine | 131053 | 131052 |
| Multiple sclerosis | G35 | Multiple sclerosis | 131043 | 131042 |
| Osteoporosis | M80  M81  M82 | Osteoporosis with pathological fracture  Osteoporosis without pathological fracture  Osteoporosis in diseases classified elsewhere | 131963  131965  131967 | 131962  131964  131966 |
| Painful conditions | M45  M46  M47  M48  M49  M50  M51  M53  M54  G44  M10  M11  M12  M13  M14  M79  B02 | Ankylosing spondylitis  Other inflammatory spondylopathies  Spondylosis  Other spondylopathies  Spondylopathies in diseases classified elsewhere  Cervical disk disorders  Other intervertebral disk disorders  Other dorsopathies, not elsewhere classified  Dorsalgia  Other headache syndromes  Gout  Other crystal arthropathies  Other specific arthropathies  Other arthritis  Arthropathies in other diseases classified elsewhere  Other soft tissue diseases, not elsewhere classified  Zoster [herpes zoster] | 131913  131915  131917  131919  131921  131923  131925  131927  131929  131055  131859  131861  131863  131865  131867  131961  130179 | 131912  131914  131916  131918  131920  131922  131924  131926  131928  131054  131858  131860  131862  131864  131866  131960  130178 |
| Parkinson’s disease | G20  G21 | Parkinson’s disease  Secondary parkinsonism | 131023  131025 | 131022  131024 |
| Peripheral vascular disease | I70  I73 | Atherosclerosis  Other peripheral vascular diseases | 131381  131387 | 131380  131386 |
| Pernicious anaemia | D51 | Vitamin B12 Deficiency Anaemia | 130625 | 130624 |
| Prostate conditions (not cancer) | N40  N41  N42 | Hyperplasia of prostate  Inflammatory diseases of prostate  Other disorders of prostate | 132073  132075  132077 | 132072  132074  132076 |
| Psoriasis/eczema | L20  L21  L22  L23  L24  L25  L26  L27  L28  L29  L30  L40  L41 | Atopic dermatitis  Seborrhoeic dermatitis  Diaper [napkin] dermatitis  Allergic contact dermatitis  Irritant contact dermatitis  Unspecified contact dermatitis  Exfoliative dermatitis  Dermatitis due to substances taken internally  Lichen simplex chronicus and prurigo  Pruritus  Other dermatitis  Psoriasis  Parapsoriasis | 131721  131723  131725  131727  131729  131731  131733  131735  131737  131739  131741  131743  131745 | 131720  131722  131724  131726  131728  131730  131732  131734  131736  131738  131740  131742  131744 |
| Stroke/transient ischaemic attack | I60  I61  I62  I63  I65  I66  I67  I68  I69 | Subarachnoid haemorrhage  Intracerebral haemorrhage  Other nontraumatic intracranial haemorrhage  Cerebral infarction  Occlusion and stenosis of precerebral arteries, not resulting in cerebral infarction  Occlusion and stenosis of cerebral arteries, not resulting in cerebral infarction  Other cerebrovascular diseases  Cerebrovascular disorders in diseases classified elsewhere  Sequelae of cerebrovascular disease | 131361  131363  131365  131367  131371  131373  131375  131377  131379 | 131360  131362  131364  131366  131370  131372  131374  131376  131378 |
| Thyroid conditions | E00  E01  E02  E03  E04  E05  E06  E07 | Congenital iodine-deficiency syndrome  Iodine-deficiency-related thyroid disorders and allied conditions  Subclinical iodine-deficiency hypothyroidism  Other hypothyroidism  Other non-toxic goitre  Thyrotoxicosis [hyperthyroidism]  Thyroiditis  Other disorders of thyroid | 130691  130693  130695  130697  130699  130701  130703  130705 | 130690  130692  130694  130696  130698  130700  130702  130704 |
| Depression | F32  F33  F34  F38  F39 | Depressive episode  Recurrent depressive disorder  Persistent mood [affective] disorders  Other mood [affective] disorders  Unspecified mood [affective] disorder | 130895  130897  130899  130901  130903 | 130894  130896  130898  130900  130902 |
| Anxiety | F41  F42  F43 | Other anxiety disorders  Obsessive-compulsive disorder  Reaction to severe stress, and adjustment disorders | 130907  130909  130911 | 130906  130908  130910 |
| Alcohol/substance dependency | F10  F11  F12  F13  F14  F15  F16  F19 | Mental and behavioral disorders due to use of alcohol  Mental and behavioral disorders due to use of opioids  Mental and behavioral disorders due to use of cannabinoids  Mental and behavioral disorders due to use of sedatives or hypnotics  Mental and behavioral disorders due to use of cocaine  Mental and behavioral disorders due to use of other stimulants, incl. caffeine  Mental and behavioral disorders due to use of hallucinogens  Mental and behavioral disorders due to use of multiple drug use and use of other psychoactive substances | 130855  130857  130859  130861  130863  130865  130867  130873 | 130854  130856  130858  130860  130862  130864  130866  130872 |
| Severe mental illness | F20  F21  F22  F23  F24  F25  F28  F29  F30  F31 | Schizophrenia  Schizotypal disorder  Persistent delusional disorders  Acute and transient psychotic disorders  Induced delusional disorder  Schizoaffective disorders  Other nonorganic psychotic disorders  Unspecified nonorganic psychosis  Manic episode  Bipolar affective disorder | 130875  130877  130879  130881  130883  130885  130887  130889  130891  130893 | 130874  130876  130878  130880  130882  130884  130886  130888  130890  130892 |
| Anorexia or bulimia | F50 | Eating disorders | 130919 | 130918 |

Abbreviation: ICD: International Classification of Diseases; ICD-10 codes for chronic conditions were based on the list of codes used to define these conditions in Ronaldson et al.^1^

**Table B. Chronic conditions used in multimorbidity definitions**

| **Full List of Conditions**  **(Count All)** | **List of 12 Most Prevalent Conditions (Count MostPrev)** | **List at the System Level Corresponding to ICD Chapter**  **(Count Syst)** |
| --- | --- | --- |
| Asthma | Asthma | 10. Diseases of the respiratory system |
| Atrial fibrillation |  | 9. Diseases of the circulatory system |
| Bronchiectasis |  | 10. Diseases of the respiratory system |
| Cancer | Cancer | 2. Neoplasms |
| Chronic kidney disease |  | 14. Diseases of the genitourinary system |
| Chronic obstructive pulmonary disorder (COPD) |  | 10. Diseases of the respiratory system |
| Chronic sinusitis |  | 10. Diseases of the respiratory system |
| Connective tissue disorders |  | 13. Diseases of the musculoskeletal system and connective tissue |
| Coronary heart disease | Coronary heart disease | 9. Diseases of the circulatory system |
| Dementia |  | 6. Diseases of the nervous system |
| Diabetes |  | 4. Endocrine, nutritional and metabolic diseases |
| Diverticular disease |  | 11. Diseases of the digestive system |
| Dyspepsia/ulcer | Dyspepsia/ulcer | 11. Diseases of the digestive system |
| Endometriosis |  | 14. Diseases of the genitourinary system |
| Epilepsy |  | 6. Diseases of the nervous system |
| Glaucoma |  | 7. Diseases of the eye and adnexa |
| Heart failure |  | 9. Diseases of the circulatory system |
| Hepatitis |  | 1. Infectious and parasitic diseases |
| Hypertension | Hypertension | 9. Diseases of the circulatory system |
| Inflammatory bowel disease | Inflammatory bowel disease | 11. Diseases of the digestive system |
| Irritable bowel syndrome | Irritable bowel syndrome | 11. Diseases of the digestive system |
| Liver disease |  | 11. Diseases of the digestive system |
| Migraine |  | 6. Diseases of the nervous system |
| Multiple sclerosis |  | 6. Diseases of the nervous system |
| Osteoporosis |  | 13. Diseases of the musculoskeletal system and connective tissue |
| Painful conditions | Painful conditions | 13. Diseases of the musculoskeletal system and connective tissue |
| Parkinson’s disease |  | 6. Diseases of the nervous system |
| Peripheral vascular disease |  | 9. Diseases of the circulatory system |
| Pernicious anaemia |  | 3. Diseases of the blood and blood-forming organs and certain disorders involving the immune mechanism |
| Prostate conditions (not cancer) |  | 14. Diseases of the genitourinary system |
| Psoriasis/eczema | Psoriasis/eczema | 12. Diseases of the skin and subcutaneous tissue |
| Stroke/transient ischaemic attack |  | 9. Diseases of the circulatory system |
| Thyroid conditions | Thyroid conditions | 4. Endocrine, nutritional and metabolic diseases |
| Depression | Depression | 5. Mental and behavioral disorders |
| Anxiety | Anxiety | 5. Mental and behavioral disorders |
| Alcohol/substance dependency |  | 5. Mental and behavioral disorders |
| Severe mental illness |  | 5. Mental and behavioral disorders |
| Anorexia or bulimia |  | 5. Mental and behavioral disorders |

Abbreviation: ICD: International Classification of Diseases

**Table C.** **Descriptive data (% (N) or Mean (SD)) on covariates and concurrent health outcomes in included( N=474,397) and excluded (N=5815) participants.**

|  | | **Included**  **(N=474,397)** | | | **Excluded**  **(N=5815)** | | |
| --- | --- | --- | --- | --- | --- | --- | --- |
|  | | **All**  **(N=474,397)** | **Men**  **(N=215,822)** | **Women**  **(N=258,575)** | **All**  **(N=5815)** | **Men**  **(N=2492)** | **Women**  **(N=3323)** |
| Age^1^ | | 56.5 (8.1) | 56.7 (8.2) | 56.3 (8.0) | 56.9 (8.4) | 56.3 (8.6) | 57.4 (8.1) |
| Townsend Deprivation Index^1^ | | -1.4 (3.1) | -1.3 (3.1) | -1.4 (3.0) | 0.39 (3.7) | 0.90 (3.6) | 0.01 (3.6) |
| Body Mass Index^1^ | | 27.4 (4.8) | 27.8 (4.2) | 27.1 (5.1) | 29.2 (6.1) | 29.0 (5.3) | 29.3 (6.6) |
| Condition  Count | All (Count All) ^1^ | 1.3 (1.6) | 1.3 (1.5) | 1.4 (1.6) | 2.1 (2.1) | 2.1 (2.1) | 2.2 (2.1) |
|  | 12 most prevalent conditions (Count MostPrev) ^1^ | 1.0 (1.2) | 1.0 (1.2) | 1.1 (1.3) | 1.6 (1.5) | 1.5 (1.5) | 1.6 (1.6) |
|  | Affected body systems (Count Syst) ^1^ | 1.2 (1.3) | 1.2 (1.3) | 1.2 (1.3) | 1.8 (1.6) | 1.7 (1.6) | 1.9 (1.6) |
| Concurrent Health Outcomes | Polypharmacy | 17.7 (83,936) | 17.1 (36,912) | 18.2 (47,024) | 36.2 (2099) | 35.1 (873) | 37.0 (1226) |
|  | Poor self-rated health | 4.2 (19,819) | 4.8 (10,425) | 3.6 (9,394) | 23.1 (859) | 26.0 (397) | 21.1 (462) |
|  | Frailty | 4.0 (19,209) | 3.2 (7,005) | 4.7 (12,204) | 14.3 (556) | 34.2 (607) | 28.8 (607) |
|  | Falls | 19.4 (92,133) | 15.5 (33,396) | 22.7 (58,737) | 36.6 (1680) | 33.5 (629) | 38.8 (1051) |
|  | Surgery | 62.0 (294,183) | 58.5 (126,249) | 64.9 (167,934) | 75.1 (4365) | 72.6 (1810) | 76.9 (2555) |
|  | Pain | 43.3 (205,232) | 40.3 (86,965) | 45.7 (118,267) | 66.1 (3175) | 64.7 (1343) | 67.2 (1832) |
| Mortality | At 3 years from baseline | 0.8 (3,873) | 1.1 (2,417) | 0.6 (1,456) | 2.1 (121) | 2.9 (73) | 1.4 (48) |
|  | At 10 years from baseline | 4.7 (22,097) | 6.1 (13,177) | 3.4 (8,920) | 10.4 (602) | 13.3 (331) | 8.2 (271) |

Abbreviation: SD: standard deviation; ^1^Mean (Standard Deviation) reported for these measures

**Table D.** **Descriptive data (% (N) or Mean (SD)) on covariates and concurrent health outcomes**

|  | | **All**  **(N=474,397)** | **Men**  **(N=215,822)** | **Women**  **(N=258,575)** |
| --- | --- | --- | --- | --- |
| Age^1^ | | 56.5 (8.1) | 56.7 (8.2) | 56.3 (8.0) |
| Townsend Deprivation Index^1^ | | -1.4 (3.1) | -1.3 (3.1) | -1.4 (3.0) |
| Body Mass Index^1^ | | 27.4 (4.8) | 27.8 (4.2) | 27.1 (5.1) |
| Condition  Count | All (Count All) ^1^ | 1.3 (1.6) | 1.3 (1.5) | 1.4 (1.6) |
|  | 12 most prevalent conditions (Count MostPrev) ^1^ | 1.0 (1.2) | 1.0 (1.2) | 1.1 (1.3) |
|  | Affected body systems (Count Syst) ^1^ | 1.2 (1.3) | 1.2 (1.3) | 1.2 (1.3) |
| Concurrent Health Outcomes | Polypharmacy | 17.7 (83,936) | 17.1 (36,912) | 18.2 (47,024) |
|  | Poor self-rated health | 4.2 (19,819) | 4.8 (10,425) | 3.6 (9,394) |
|  | Frailty | 4.0 (19,209) | 3.2 (7,005) | 4.7 (12,204) |
|  | Falls | 19.4 (92,133) | 15.5 (33,396) | 22.7 (58,737) |
|  | Surgery | 62.0 (294,183) | 58.5 (126,249) | 64.9 (167,934) |
|  | Pain | 43.3 (205,232) | 40.3 (86,965) | 45.7 (118,267) |
| Mortality | At 3 years from baseline | 0.8 (3,873) | 1.1 (2,417) | 0.6 (1,456) |
|  | At 10 years from baseline | 4.7 (22,097) | 6.1 (13,177) | 3.4 (8,920) |

^1^Mean (Standard Deviation) reported for these measures

| **Multimorbidity measures^1^** | | | **Prevalence** | **Condition Count^2,3^** | **Age at baseline^2^** | **Concurrent Health Outcomes** | | | | | | **Mortality** | |
| --- | --- | --- | --- | --- | --- | --- | --- | --- | --- | --- | --- | --- | --- |
|  |  |  |  |  |  | **Polypharmacy** | **Poor self-rated health** | **Frailty** | **Falls** | **Surgery** | **Chronic Pain** | **3 years** | **10 years** |
| **Count**  **All** | **2+** | No Multimorbidity | 66.0 (142,460) | 0.4 (0.5) | 55.3 (8.2) | 6.8 (9,686) | 2.0 (2,831) | 1.5 (2,117) | 13.3 (18,913) | 47.1 (67,036) | 35.4 (50,401) | 0.7 (1,025) | 4.2 (6,017) |
|  |  | Multimorbidity | 34.0 (73,362) | 3.1 (1.4) | 59.5 (7.4) | 37.1 (27,226) | 10.4 (7,594) | 6.7 (4,888) | 19.7 (14,483) | 80.7 (59,213) | 49.8 (36,564) | 1.9 (1,392) | 9.8 (7,160) |
|  | **3+** | No Multimorbidity | 82.0 (176,898) | 0.7 (0.8) | 55.9 (8.2) | 10.1 (17,933) | 2.7 (4,769) | 1.9 (3,328) | 14.0 (24,702) | 52.3 (92,435) | 37.2 (65,840) | 0.8 (1,491) | 4.8 (8,480) |
|  |  | Multimorbidity | 18.0 (38,924) | 4.0 (1.3) | 60.3 (7.1) | 48.8 (18,979) | 14.5 (5,656) | 9.4 (3,677) | 22.3 (8,694) | 86.9 (33,814) | 54.3 (21,125) | 2.4 (926) | 12.1 (4,697) |
| **Count MostPrev** | **2+** | No Multimorbidity | 73.6 (158,740) | 0.4 (0.5) | 55.7 (8.2) | 9.1 (14,393) | 2.6 (4,104) | 1.9 (2,994) | 13.8 (21,926) | 50.5 (80,199) | 36.2 (57,516) | 0.8 (1,324) | 4.8 (7,618) |
|  |  | Multimorbidity | 26.4 (57,082) | 2.6 (0.9) | 59.4 (7.4) | 39.5 (22,519) | 11.1 (6,321) | 7.0 (4,011) | 20.1 (11,470) | 80.7 (46,050) | 51.6 (29,449) | 1.9 (1,093) | 9.7 (5,559) |
|  | **3+** | No Multimorbidity | 89.0 (192,155) | 0.7 (0.8) | 56.3 (8.2) | 12.9 (24,705) | 3.5 (6,639) | 2.4 (4,580) | 14.5 (27,928) | 55.0 (105,671) | 38.2 (73,392) | 1.0 (1,882) | 5.4 (10,446) |
|  |  | Multimorbidity | 11.0 (23,667) | 3.5 (0.8) | 60.0 (7.2) | 51.6 (12,207) | 16.0 (3,786) | 10.2 (2,425) | 23.1 (5,468) | 86.9 (20,578) | 57.3 (13,573) | 2.3 (535) | 11.5 (2,731) |
| **Count**  **Syst** | **2+** | No Multimorbidity | 68.2 (147,241) | 0.4 (0.5) | 55.4 (8.2) | 8.0 (11,720) | 2.2 (3,235) | 1.6 (2,361) | 13.4 (19,717) | 48.4 (71,295) | 35.6 (52,425) | 0.8 (1,114) | 4.4 (6,494) |
|  |  | Multimorbidity | 31.8 (68,581) | 2.7 (1.0) | 59.5 (7.4) | 36.7 (25,192) | 10.5 (7,190) | 6.8 (4,644) | 19.9 (13,679) | 80.1 (54,954) | 50.4 (34,540) | 1.9 (1,303) | 9.7 (6,683) |
|  | **3+** | No Multimorbidity | 85.3 (184,139) | 0.7 (0.8) | 56.1 (8.2) | 11.8 (21,753) | 3.1 (5,696) | 2.1 (3,898) | 14.2 (26,108) | 53.8 (99,063) | 37.7 (69,450) | 0.9 (1,653) | 5.1 (9,340) |
|  |  | Multimorbidity | 14.7 (31,683) | 3.6 (0.9) | 60.3 (7.1) | 47.8 (15,159) | 14.9 (4,729) | 9.8 (3,107) | 23.0 (7,288) | 85.8 (27,186) | 55.3 (17,515) | 2.4 (764) | 12.1 (3,837) |
| **Clustering** | | No Multimorbidity | 66.0 (142,460) | 0.4 (0.5) | 55.3 (8.2) | 6.8 (9,686) | 2.0 (2,831) | 1.5 (2,117) | 13.3 (18,913) | 47.1 (67,036) | 35.4 (50,401) | 0.7 (1,025) | 4.2 (6,017) |
|  |  | Mental Disorders | 3.7 (7,994) | 3.1 (1.3) | 56.9 (7.9) | 30.9 (2,474) | 13.0 (1,039) | 7.8 (622) | 23.3 (1,861) | 75.5 (6,039) | 51.7 (4,129) | 1.6 (125) | 8.5 (682) |
|  |  | Respiratory | 4.9 (10,490) | 3.2 (1.4) | 58.6 (7.8) | 41.9 (4,394) | 13.2 (1,382) | 8.0 (842) | 20.3 (2,126) | 76.8 (8,053) | 51.0 (5,348) | 1.8 (186) | 10.1 (1,061) |
|  |  | Digestive + Osteoporosis | 4.1 (8,929) | 3.5 (1.6) | 59.7 (7.5) | 31.7 (2,831) | 11.0 (978) | 6.5 (583) | 18.8 (1,677) | 90.8 (8,106) | 53.9 (4,815) | 2.2 (197) | 9.3 (831) |
|  |  | Painful Conditions + Cardiovascular and Metabolic Disorders | 10.5 (22,605) | 2.8 (1.1) | 59.1 (7.4) | 35.5 (8,028) | 8.1 (1,838) | 6.1 (1,381) | 19.0 (4,284) | 73.6 (16,635) | 49.7 (11,225) | 1.4 (310) | 8.1 (1,826) |
|  |  | Hypertension + Other | 8.2 (17,769) | 2.5 (0.8) | 61.6 (6.3) | 36.7 (6,524) | 6.8 (1,216) | 4.2 (748) | 17.8 (3,156) | 86.0 (15,285) | 45.6 (8,098) | 2.0 (352) | 9.9 (1759) |
|  |  | Liver + Alcohol + Dementia | 1.2 (2,518) | 4.2 (2.0) | 57.6 (7.6) | 41.6 (1,048) | 20.7 (521) | 12.8 (323) | 26.3 (662) | 87.8 (2,211) | 54.2 (1,364) | 4.3 (108) | 18.2 (459) |
|  |  | Circulatory | 1.4 (3,057) | 4.9 (2.0) | 60.9 (6.7) | 63.0 (1,927) | 20.3 (620) | 12.7 (389) | 23.5 (717) | 94.3 (2,884) | 51.8 (1,585) | 3.7 (114) | 17.7 (542) |

**Table E.**  **Descriptive data (% (N) or Mean (SD)) on concurrent health outcomes and mortality as a function of multimorbidity measures in MEN**

**Abbreviations**: Count MostPrev = 12 most prevalent conditions in the list; Count Syst = count of body-systems involved; 2+ = multimorbidity defined using 2 or more conditions; 3+ = multimorbidity defined using 3 or more conditions.

^1^Multimorbidity definitions are based on counting conditions or clustering. Count All, Count MostPrev and Count Syst, count the number of all conditions, 12 most prevalent conditions, or number of body systems involved by conditions, respectively. For count-based approaches, 2+ or 3+ thresholds were used to categorize participants as multimorbid or not. Cluster groups were identified among those multimorbid based on the Count All 2+ Measure.

^2^Mean (SD) reported for these metrics

^3^The condition count is reported using the respective list for each method

**Table F.** **Descriptive data (% (N) or Mean (SD)) on concurrent health outcomes and mortality as a function of multimorbidity measures in WOMEN**

| **Multimorbidity measures^1^** | | | **Prevalence** | **Condition Count^2,3^** | **Age at baseline^2^** | **Concurrent Health Outcomes** | | | | | | **Mortality** | |
| --- | --- | --- | --- | --- | --- | --- | --- | --- | --- | --- | --- | --- | --- |
|  |  |  |  |  |  | **Polypharmacy** | **Poor self-rated health** | **Frailty** | **Falls** | **Surgery** | **Chronic Pain** | **3 years** | **10 years** |
| **Count**  **All** | **2+** | No Multimorbidity | 64.7 (167,314) | 0.4 (0.5) | 55.3 (8.0) | 9.1 (15,233) | 1.4 (2,342) | 2.4 (3,940) | 19.9 (33,248) | 55.8 (93,427) | 39.7 (66,363) | 0.4 (691) | 2.5 (4,254) |
|  |  | Multimorbidity | 35.3 (91,261) | 3.1 (1.4) | 58.2 (7.6) | 34.8 (31,791) | 7.7 (7,052) | 9.1 (8,264) | 27.9 (25,489) | 81.6 (74,507) | 56.9 (51,904) | 0.8 (765) | 5.1 (4,666) |
|  | **3+** | No Multimorbidity | 80.9 (209,058) | 0.7 (0.8) | 55.8 (8.0) | 12.0 (25,101) | 1.9 (4,014) | 3.0 (6,218) | 20.8 (43,512) | 59.8 (125,119) | 41.9 (87,570) | 0.5 (977) | 2.8 (5,952) |
|  |  | Multimorbidity | 19.1 (49,517) | 4.0 (1.4) | 58.7 (7.5) | 44.3 (21,923) | 10.9 (5,380) | 12.1 (5,986) | 30.7 (15,225) | 86.5 (42,815) | 62.0 (30,697) | 1.0 (479) | 6.0 (2,968) |
| **Count MostPrev** | **2+** | No Multimorbidity | 70.9 (183,456) | 0.4 (0.5) | 55.5 (8.0) | 10.8 (19,753) | 1.8 (3,308) | 2.8 (5,096) | 20.4 (37,419) | 58.0 (106,424) | 40.8 (74,790) | 0.4 (801) | 2.8 (5,076) |
|  |  | Multimorbidity | 29.1 (75,119) | 2.8 (1.0) | 58.3 (7.6) | 36.3 (27,271) | 8.1 (6,086) | 9.5 (7,108) | 28.4 (21,318) | 81.9 (61,510) | 57.9 (43,477) | 0.9 (655) | 5.1 (3,844) |
|  | **3+** | No Multimorbidity | 86.6 (224,009) | 0.7 (0.8) | 56.0 (8.0) | 13.9 (31,029) | 2.4 (5,346) | 3.5 (7,792) | 21.4 (47,849) | 61.5 (137,858) | 43.0 (96,228) | 0.5 (1,121) | 3.1 (6,890) |
|  |  | Multimorbidity | 13.4 (34,566) | 3.7 (1.0) | 58.7 (7.5) | 46.3 (15,995) | 11.7 (4,048) | 12.8 (4,412) | 31.5 (10,888) | 87.0 (30,076) | 63.8 (22,039) | 1.0 (335) | 5.9 (2,030) |
| **Count**  **Syst** | **2+** | No Multimorbidity | 66.1 (170,924) | 0.4 (0.5) | 55.4 (8.0) | 9.5 (16,297) | 1.5 (2,548) | 2.4 (4,180) | 20.0 (34,161) | 56.4 (96,482) | 39.9 (68,245) | 0.4 (717) | 2.6 (4,428) |
|  |  | Multimorbidity | 33.9 (87,651) | 2.8 (1.0) | 58.2 (7.6) | 35.1 (30,727) | 7.8 (6,846) | 9.2 (8,024) | 28.0 (24,576) | 81.5 (71,452) | 57.1 (50,022) | 0.8 (739) | 5.1 (4,492) |
|  | **3+** | No Multimorbidity | 83.4 (215,715) | 0.8 (0.8) | 55.9 (8.0) | 12.9 (27,834) | 2.1 (4,574) | 3.2 (6,866) | 21.1 (45,452) | 60.7 (130,978) | 42.4 (91,454) | 0.5 (1,043) | 2.9 (6,328) |
|  |  | Multimorbidity | 16.6 (42,860) | 3.7 (0.9) | 58.7 (7.5) | 44.8 (19,190) | 11.2 (4,820) | 12.5 (5,338) | 31.0 (13,285) | 86.2 (36,956) | 62.6 (26,813) | 1.0 (413) | 6.0 (2,592) |
| **Clustering** | | No Multimorbidity | 64.7 (167,314) | 0.4 (0.5) | 55.3 (8.0) | 9.1 (15,233) | 1.4 (2,342) | 2.4 (3,940) | 19.9 (33,248) | 55.8 (93,427) | 39.7 (66,363) | 0.4 (691) | 2.5 (4,254) |
|  |  | Mental Disorders | 1.1 (2,768) | 3.7 (1.9) | 54.6 (7.9) | 34.3 (950) | 14.3 (395) | 13.4 (370) | 32.3 (895) | 82.4 (2,280) | 56.1 (1,553) | 1.6 (45) | 7.9 (220) |
|  |  | Respiratory | 1.0 (2,680) | 4.2 (2.0) | 60.0 (6.9) | 51.6 (1,382) | 18.8 (503) | 17.3 (464) | 30.8 (825) | 85.1 (2,281) | 60.5 (1,622) | 1.8 (49) | 11.4 (306) |
|  |  | Digestive | 9.2 (23,868) | 3.2 (1.5) | 58.7 (7.4) | 34.7 (8,288) | 7.4 (1,765) | 8.7 (2,077) | 27.4 (6,546) | 89.7 (21,414) | 60.9 (14,526) | 0.7 (171) | 4.3 (1,032) |
|  |  | Painful Conditions + Other | 13.7 (35,516) | 2.7 (1.0) | 58.1 (7.6) | 27.4 (9,730) | 4.8 (1,705) | 6.5 (2,316) | 25.6 (9,109) | 74.8 (26,560) | 52.5 (18,656) | 0.8 (267) | 4.4 (1,563) |
|  |  | Asthma + Other | 5.3 (13,721) | 3.1 (1.4) | 57.0 (8.0) | 42.6 (5,851) | 8.3 (1,140) | 9.7 (1,327) | 29.3 (4,017) | 80.9 (11,105) | 59.1 (8,114) | 0.6 (87) | 4.6 (626) |
|  |  | Connective + Osteoporosis + Neurological | 4.9 (12,708) | 3.5 (1.7) | 59.4 (7.3) | 44.0 (5,590) | 12.1 (1,544) | 13.5 (1,710) | 32.2 (4,097) | 85.5 (10,867) | 58.5 (7,433) | 1.1 (146) | 7.2 (919) |

**Abbreviations**: Count MostPrev = 12 most prevalent conditions in the list; Count Syst = count of body-systems involved; 2+ = multimorbidity defined using 2 or more conditions; 3+ = multimorbidity defined using 3 or more conditions.

^1^Multimorbidity definitions are based on counting conditions or clustering. Count All, Count MostPrev and Count Syst, count the number of all conditions, 12 most prevalent conditions, or number of body systems involved by conditions, respectively. For count-based approaches, 2+ or 3+ thresholds were used to categorize participants as multimorbid or not. Cluster groups were identified among those multimorbid based on the Count All 2+ Measure.

^2^Mean (SD) reported for these metrics

^3^The condition count is reported using the respective list for each method

**Table G. Multimorbidity prevalence, overall and by age groups, defined using terciles, in men and women.**

| **MEN (N=215,822), mean (SD) age: 56.7 (8.2) years** | |  | **Age groups** | | |
| --- | --- | --- | --- | --- | --- |
| **Multimorbidity measures^1^** | | **All (N=215,822**) | **From 38 to 53**  **(N= 68,501)** | **From 53 to 61**  **(N= 61,290)** | **From 61 to 73**  **(N= 86,031)** |
| **Count**  **All** | **2+** | 34.0 | 20.2 | 32.3 | 46.2 |
|  | **3+** | 18.0 | 8.9 | 16.4 | 26.5 |
| **Count MostPrev** | **2+** | 26.4 | 16.1 | 25.2 | 35.5 |
|  | **3+** | 11.0 | 5.8 | 10.2 | 15.6 |
| **Count**  **Syst** | **2+** | 31.8 | 19.0 | 30.2 | 43.1 |
|  | **3+** | 14.7 | 7.2 | 13.3 | 21.6 |
| **Clustering** | Mental Disorders | 3.7 | 3.5 | 3.9 | 3.7 |
|  | Respiratory | 4.9 | 3.5 | 4.6 | 6.1 |
|  | Digestive + Osteoporosis | 4.1 | 2.4 | 3.7 | 5.8 |
|  | Painful Conditions + **Cardiovascular and Metabolic disorders** | 10.5 | 6.6 | 10.6 | 13.5 |
|  | Hypertension + Other | 8.2 | 2.6 | 7.0 | 13.7 |
|  | Liver + Alcohol + Dementia | 1.2 | 1.0 | 1.3 | 1.2 |
|  | Circulatory | 1.4 | 0.6 | 1.2 | 2.2 |
| **WOMEN (N=258,575), mean (SD) age: 56.3 (8.0) years** | |  | **Age groups** | | |
| **Multimorbidity measures^1^** | | **All (N=258,575**) | **From 38 to 53**  **(N= 85,176)** | **From 53 to 61**  **(N=78,186)** | **From 61 to 73**  **(N= 95,213)** |
| **Count**  **All** | **2+** | 35.3 | 25.9 | 34.4 | 44.4 |
|  | **3+** | 19.1 | 12.6 | 18.4 | 25.6 |
| **Count MostPrev** | **2+** | 29.1 | 21.0 | 28.5 | 36.7 |
|  | **3+** | 13.4 | 8.9 | 13.0 | 17.7 |
| **Count**  **Syst** | **2+** | 33.9 | 25.9 | 33.1 | 42.7 |
|  | **3+** | 16.6 | 11.0 | 16.0 | 22.1 |
| **Clustering** | Mental Disorders | 1.1 | 1.3 | 1.1 | 0.8 |
|  | Respiratory | 1.0 | 0.5 | 1.0 | 1.6 |
|  | Digestive | 9.2 | 6.1 | 9.0 | 12.2 |
|  | Painful Conditions + Other | 13.7 | 10.2 | 13.6 | 17.0 |
|  | Asthma + Other | 5.3 | 4.9 | 5.1 | 5.9 |
|  | Connective + Osteoporosis + Neurological | 4.9 | 2.8 | 4.6 | 7.0 |

**Abbreviations**: Count MostPrev = 12 most prevalent conditions in the list; Count Syst = count of body-systems involved; 2+ = multimorbidity defined using 2 or more conditions; 3+ = multimorbidity defined using 3 or more conditions

^1^Multimorbidity definitions are based on counting conditions or clustering. Count All, Count MostPrev and Count Syst, count the number of all conditions, 12 most prevalent conditions, or number of body systems involved by conditions, respectively. For count-based approaches, 2+ or 3+ thresholds were used to categorize participants as multimorbid or not. Cluster groups were identified among those multimorbid based on the Count All: 2+ Measure.

**Table H:** **Cross-sectional associations of multimorbidity measures with health outcomes in men and women (unadjusted results).**

| **Multimorbidity measures^1^** | | **Prevalence**  % | **Health outcomes (Odds Ratio (95% Confidence Interval)^2^** | | | | | |
| --- | --- | --- | --- | --- | --- | --- | --- | --- |
|  |  |  | **Polypharmacy** | **Poor self-rated**  **health** | **Frailty** | **Falls** | **Surgery** | **Chronic Pain** |
| **MEN (N=215,822), mean (SD) age: 56.7 (8.2) years** | | | | | | | | |
| **Count**  **All** | **2+** | 34.0 | 8.09 (7.89, 8.3) | 5.69 (5.45, 5.95) | 4.73 (4.49, 4.98) | 1.61 (1.57, 1.65) | 4.71 (4.61, 4.81) | 1.81 (1.78, 1.85) |
|  | **3+** | 18.0 | 8.44 (8.23, 8.65) | 6.14 (5.89, 6.39) | 5.44 (5.18, 5.71) | 1.77 (1.72, 1.82) | 6.05 (5.86, 6.24) | 2 (1.96, 2.05) |
| **Count MostPrev** | **2+** | 26.4 | 6.53 (6.38, 6.69) | 4.69 (4.51, 4.89) | 3.93 (3.75, 4.13) | 1.57 (1.53, 1.61) | 4.09 (4, 4.18) | 1.88 (1.84, 1.91) |
|  | **3+** | 11.0 | 7.22 (7.02, 7.43) | 5.32 (5.1, 5.55) | 4.68 (4.44, 4.92) | 1.77 (1.71, 1.83) | 5.45 (5.25, 5.67) | 2.18 (2.12, 2.24) |
| **Count**  **Syst** | **2+** | 31.8 | 6.71 (6.55, 6.88) | 5.21 (5, 5.44) | 4.46 (4.24, 4.69) | 1.61 (1.57, 1.65) | 4.3 (4.21, 4.39) | 1.84 (1.8, 1.87) |
|  | **3+** | 14.7 | 6.85 (6.67, 7.03) | 5.5 (5.28, 5.72) | 5.03 (4.79, 5.28) | 1.81 (1.76, 1.86) | 5.19 (5.02, 5.37) | 2.04 (1.99, 2.09) |
| **Clustering** | Mental Disorders | 3.7 | 6.14 (5.83, 6.47) | 7.37 (6.83, 7.94) | 5.59 (5.1, 6.13) | 1.98 (1.88, 2.09) | 3.48 (3.3, 3.66) | 1.95 (1.87, 2.04) |
|  | Respiratory | 4.9 | 9.88 (9.46, 10.32) | 7.48 (6.99, 8.01) | 5.79 (5.33, 6.28) | 1.66 (1.58, 1.75) | 3.72 (3.55, 3.9) | 1.9 (1.83, 1.98) |
|  | Digestive + Osteoporosis | 4.1 | 6.36 (6.06, 6.68) | 6.07 (5.62, 6.54) | 4.63 (4.21, 5.09) | 1.51 (1.43, 1.6) | 11.08 (10.31, 11.92) | 2.14 (2.05, 2.23) |
|  | Painful Conditions + **Cardiovascular and Metabolic disorders** | 10.5 | 7.55 (7.3, 7.81) | 4.37 (4.11, 4.64) | 4.31 (4.02, 4.62) | 1.53 (1.47, 1.58) | 3.14 (3.04, 3.24) | 1.8 (1.75, 1.85) |
|  | Hypertension + Other | 8.2 | 7.95 (7.67, 8.25) | 3.62 (3.38, 3.88) | 2.91 (2.67, 3.17) | 1.41 (1.35, 1.47) | 6.92 (6.63, 7.23) | 1.53 (1.48, 1.58) |
|  | Liver + Alcohol + Dementia | 1.2 | 9.77 (9, 10.6) | 12.87 (11.6, 14.26) | 9.76 (8.6, 11.03) | 2.33 (2.13, 2.55) | 8.1 (7.2, 9.15) | 2.16 (1.99, 2.34) |
|  | Circulatory | 1.4 | 23.38 (21.66, 25.23) | 12.55 (11.4, 13.8) | 9.67 (8.61, 10.83) | 2.00 (1.84, 2.18) | 18.76 (16.14, 21.95) | 1.97 (1.83, 2.11) |
| **WOMEN (N=258,575), mean (SD) age: 56.3 (8.0) years** | | | | | | | | |
| **Count**  **All** | **2+** | 35.3 | 5.34 (5.22, 5.45) | 5.9 (5.63, 6.19) | 4.13 (3.97, 4.29) | 1.56 (1.53, 1.59) | 3.52 (3.45, 3.59) | 2.01 (1.97, 2.04) |
|  | **3+** | 19.1 | 5.82 (5.7, 5.95) | 6.23 (5.97, 6.49) | 4.49 (4.32, 4.65) | 1.69 (1.65, 1.73) | 4.29 (4.17, 4.4) | 2.26 (2.22, 2.31) |
| **Count MostPrev** | **2+** | 29.1 | 4.72 (4.63, 4.82) | 4.8 (4.6, 5.01) | 3.66 (3.53, 3.8) | 1.55 (1.52, 1.58) | 3.27 (3.2, 3.34) | 2 (1.96, 2.03) |
|  | **3+** | 13.4 | 5.36 (5.23, 5.49) | 5.43 (5.2, 5.66) | 4.06 (3.91, 4.22) | 1.69 (1.65, 1.74) | 4.19 (4.05, 4.32) | 2.34 (2.28, 2.39) |
| **Count**  **Syst** | **2+** | 33.9 | 5.12 (5.01, 5.23) | 5.6 (5.35, 5.86) | 4.02 (3.87, 4.18) | 1.56 (1.53, 1.59) | 3.4 (3.34, 3.47) | 2 (1.97, 2.03) |
|  | **3+** | 16.6 | 5.47 (5.35, 5.6) | 5.85 (5.61, 6.1) | 4.33 (4.17, 4.49) | 1.68 (1.64, 1.72) | 4.05 (3.93, 4.17) | 2.27 (2.22, 2.32) |
| **Clustering** | Mental Disorders | 1.1 | 5.22 (4.81, 5.65) | 11.73 (10.45, 13.13) | 6.4 (5.7, 7.16) | 1.93 (1.78, 2.09) | 3.69 (3.35, 4.08) | 1.94 (1.8, 2.1) |
|  | Respiratory | 1.0 | 10.63 (9.84, 11.49) | 16.28 (14.64, 18.07) | 8.68 (7.81, 9.63) | 1.79 (1.65, 1.95) | 4.52 (4.07, 5.04) | 2.33 (2.16, 2.52) |
|  | Digestive | 9.2 | 5.31 (5.15, 5.48) | 5.62 (5.28, 5.99) | 3.95 (3.74, 4.18) | 1.52 (1.48, 1.57) | 6.9 (6.61, 7.2) | 2.37 (2.3, 2.43) |
|  | Painful Conditions + Other | 13.7 | 3.77 (3.66, 3.88) | 3.55 (3.33, 3.78) | 2.89 (2.74, 3.05) | 1.39 (1.35, 1.43) | 2.35 (2.29, 2.41) | 1.68 (1.64, 1.72) |
|  | Asthma + Other | 5.3 | 7.42 (7.15, 7.71) | 6.38 (5.93, 6.86) | 4.44 (4.16, 4.74) | 1.67 (1.61, 1.73) | 3.36 (3.21, 3.51) | 2.2 (2.12, 2.28) |
|  | Connective + Osteoporosis + Neurological | 4.9 | 7.84 (7.54, 8.15) | 9.74 (9.11, 10.42) | 6.45 (6.07, 6.84) | 1.92 (1.84, 1.99) | 4.67 (4.44, 4.91) | 2.14 (2.07, 2.22) |

**Abbreviations**: Count MostPrev = 12 most prevalent conditions in the list; Count Syst = count of body-systems involved; 2+ = multimorbidity defined using 2 or more conditions; 3+ = multimorbidity defined using 3 or more conditions

**^1^**Multimorbidity definitions are based on counting conditions or clustering. Count All, Count MostPrev and Count Syst count the number of all conditions, 12 most prevalent conditions or number of body-systems affected, respectively. Cluster groups were identified among those multimorbid based on the Count All: 2+ Measure.

**^2^**Logistic regression adjusted for age, Townsend deprivation index, ethnicity, and body mass index. Odds Ratios represent the odds of a health outcome among those with the corresponding multimorbid status, as compared to the reference group of those identified as “not multimorbid”.

**Table I. Standardized mean value of blood-based measures as a function of multimorbidity measures**

| **Multimorbidity measures^1^** | | | **Prevalence, %** | **Blood-based measures^3^** | | | | |
| --- | --- | --- | --- | --- | --- | --- | --- | --- |
|  |  |  |  | **C-reactive protein** | **Cystatin C** | **HDL Cholesterol** | **IGF-1** | **LDL direct** |
| **MEN (N=186,873)^2^** | | | | | | | | |
| **Count**  **All** | **2+** | No Multimorbidity | 66.2 (123,674) | -0.06 | -0.14 | 0.06 | 0.06 | 0.14 |
|  |  | Multimorbidity | 33.8 (63,199) | 0.12 | 0.27 | -0.11 | -0.12 | -0.27 |
|  | **3+** | No Multimorbidity | 82.1 (153,413) | -0.04 | -0.09 | 0.04 | 0.03 | 0.09 |
|  |  | Multimorbidity | 17.9 (33,460) | 0.19 | 0.43 | -0.17 | -0.16 | -0.39 |
| **Count MostPrev** | **2+** | No Multimorbidity | 73.6 (137,617) | -0.05 | -0.10 | 0.05 | 0.04 | 0.10 |
|  |  | Multimorbidity | 26.4 (49,256) | 0.13 | 0.29 | -0.13 | -0.12 | -0.28 |
|  | **3+** | No Multimorbidity | 89.1 (166,567) | -0.02 | -0.05 | 0.02 | 0.02 | 0.05 |
|  |  | Multimorbidity | 10.9 (20,306) | 0.19 | 0.42 | -0.19 | -0.15 | -0.39 |
| **Count Syst** | **2+** | No Multimorbidity | 68.4 (127,783) | -0.06 | -0.13 | 0.05 | 0.05 | 0.12 |
|  |  | Multimorbidity | 31.6 (59,090) | 0.13 | 0.27 | -0.11 | -0.12 | -0.25 |
|  | **3+** | No Multimorbidity | 85.4 (159,645) | -0.03 | -0.08 | 0.03 | 0.03 | 0.06 |
|  |  | Multimorbidity | 14.6 (27,228) | 0.19 | 0.44 | -0.15 | -0.15 | -0.36 |
| **Clustering** | | No multimorbidity | 66.2 (123,674) | -0.06 | -0.14 | 0.06 | 0.06 | 0.14 |
|  |  | Mental Disorders | 3.7 (6,844) | 0.07 | 0.09 | -0.13 | -0.04 | -0.07 |
|  |  | Respiratory | 4.9 (9,108) | 0.22 | 0.17 | 0.02 | -0.13 | -0.13 |
|  |  | Digestive + Osteoporosis | 4.1 (7,672) | 0.15 | 0.31 | -0.08 | -0.06 | -0.15 |
|  |  | Painful Conditions + Cardiovascular and Metabolic Disorders | 10.4 (19,478) | 0.12 | 0.29 | -0.14 | -0.13 | -0.32 |
|  |  | Hypertension + Other | 8.2 (15,350) | 0.05 | 0.25 | -0.12 | -0.10 | -0.35 |
|  |  | Liver + Alcohol + Dementia | 1.1 (2,131) | 0.33 | 0.55 | -0.16 | -0.47 | -0.38 |
|  |  | Circulatory | 1.4 (2,616) | 0.17 | 0.73 | -0.37 | -0.20 | -0.66 |
| **WOMEN (N=218,781)^2^** | | | | | | | | |
| **Count**  **All** | **2+** | No Multimorbidity | 64.9 (141,917) | -0.08 | -0.14 | 0.07 | 0.07 | 0.02 |
|  |  | Multimorbidity | 35.1 (76,864) | 0.15 | 0.25 | -0.13 | -0.14 | -0.05 |
|  | **3+** | No Multimorbidity | 81.0 (177,152) | -0.05 | -0.09 | 0.04 | 0.04 | 0.02 |
|  |  | Multimorbidity | 19.0 (41,629) | 0.22 | 0.38 | -0.19 | -0.19 | -0.09 |
| **Count MostPrev** | **2+** | No Multimorbidity | 71.1 (155,525) | -0.07 | -0.11 | 0.06 | 0.06 | 0.02 |
|  |  | Multimorbidity | 28.9 (63,256) | 0.16 | 0.27 | -0.15 | -0.15 | -0.05 |
|  | **3+** | No Multimorbidity | 86.7 (189,770) | -0.04 | -0.06 | 0.03 | 0.03 | 0.01 |
|  |  | Multimorbidity | 13.3 (29,011) | 0.24 | 0.39 | -0.22 | -0.20 | -0.08 |
| **Count Syst** | **2+** | No Multimorbidity | 66.2 (144,937) | -0.08 | -0.13 | 0.07 | 0.07 | 0.02 |
|  |  | Multimorbidity | 33.8 (73,844) | 0.16 | 0.26 | -0.13 | -0.14 | -0.04 |
|  | **3+** | No Multimorbidity | 83.5 (182,743) | -0.05 | -0.08 | 0.04 | 0.04 | 0.02 |
|  |  | Multimorbidity | 16.5 (36,038) | 0.23 | 0.39 | -0.19 | -0.19 | -0.08 |
| **Clustering** | | No Multimorbidity | 64.9 (141,917) | -0.08 | -0.14 | 0.07 | 0.07 | 0.02 |
|  |  | Mental Disorders | 1.0 (2,294) | 0.18 | 0.22 | -0.10 | -0.12 | -0.06 |
|  |  | Respiratory | 1.0 (2,266) | 0.50 | 0.50 | -0.04 | -0.23 | -0.06 |
|  |  | Digestive | 9.2 (20,156) | 0.17 | 0.25 | -0.21 | -0.18 | -0.03 |
|  |  | Painful Conditions + Other | 13.6 (29,834) | 0.07 | 0.22 | -0.13 | -0.09 | -0.05 |
|  |  | Asthma + Other | 5.3 (11,647) | 0.23 | 0.18 | -0.13 | -0.15 | -0.05 |
|  |  | Connective + Osteoporosis + Neurological | 4.9 (10,667) | 0.20 | 0.39 | -0.02 | -0.16 | -0.07 |

**Abbreviations**: Count MostPrev = 12 most prevalent conditions in the list; Count Syst = count of body-systems involved; 2+ = multimorbidity defined using 2 or more conditions; 3+ = multimorbidity defined using 3 or more conditions; HDL = High-Density Lipoprotein; IGF = Insulin-like Growth Factor; LDL = low-density lipoprotein.

^1^Multimorbidity definitions are based on counting conditions or clustering. Count All, Count MostPrev and Count Syst, count the number of all conditions, 12 most prevalent conditions, or number of body systems involved by conditions, respectively. For count-based approaches, 2+ or 3+ thresholds were used to categorize participants as multimorbid or not. Cluster groups were identified among those multimorbid based on the Count All 2+ Measure.

^2^These analyses were completed among all 405,654 participants (# Men = 186,873, # Women =218,781) with fully observed biomarker data

^3^Blood-based measures were standardized to have mean 0 and standard deviation equal to 1, separately in men and women.

**Table J.** **Cross-sectional association of** **multimorbidity measures with blood-based measures**

| **Multimorbidity measures^1^** | | **Prevalence, %** | **Blood-based measures,^3^**  **Estimate (95% Confidence Interval)^4^** | | | | |
| --- | --- | --- | --- | --- | --- | --- | --- |
|  |  |  | **C-reactive**  **protein** | **Cystatin**  **C** | **HDL**  **Cholesterol** | **IGF-1** | **LDL**  **cholesterol** |
| **MEN (N=186,873)^2^** | | | | | | | |
| **Count**  **All** | **2+** | 33.8 | 0.12 (0.11, 0.13) | 0.22 (0.21, 0.23) | -0.11 (-0.12, -0.10) | -0.04 (-0.05, -0.03) | -0.35 (-0.36, -0.34) |
|  | **3+** | 17.9 | 0.14 (0.13, 0.16) | 0.32 (0.31, 0.33) | -0.13 (-0.14, -0.12) | -0.04 (-0.05, -0.03) | -0.42 (-0.43, -0.40) |
| **Count MostPrev** | **2+** | 26.4 | 0.10 (0.09, 0.11) | 0.21 (0.20, 0.22) | -0.10 (-0.11, -0.09) | -0.03 (-0.04, -0.02) | -0.32 (-0.33, -0.31) |
|  | **3+** | 10.9 | 0.13 (0.12, 0.15) | 0.28 (0.26, 0.29) | -0.12 (-0.13, -0.11) | -0.03 (-0.04, -0.01) | -0.38 (-0.39, -0.36) |
| **Count**  **Syst** | **2+** | 31.6 | 0.12 (0.11, 0.13) | 0.22 (0.21, 0.23) | -0.09 (-0.10, -0.08) | -0.04 (-0.05, -0.03) | -0.31 (-0.32, -0.30) |
|  | **3+** | 14.6 | 0.14 (0.13, 0.16) | 0.32 (0.31, 0.33) | -0.10 (-0.11, -0.08) | -0.03 (-0.04, -0.02) | -0.35 (-0.36, -0.34) |
| **Clustering** | Mental Disorders | 3.7 | 0.08 (0.06, 0.10) | 0.12 (0.10, 0.14) | -0.12 (-0.14, -0.09) | -0.03 (-0.05, 0.00) | -0.18 (-0.20, -0.16) |
|  | Respiratory | 4.9 | 0.21 (0.19, 0.23) | 0.15 (0.13, 0.17) | 0.03 (0.01, 0.05) | -0.07 (-0.09, -0.05) | -0.22 (-0.24, -0.20) |
|  | Digestive + Osteoporosis | 4.1 | 0.17 (0.14, 0.19) | 0.29 (0.26, 0.31) | -0.13 (-0.16, -0.11) | 0.01 (-0.02, 0.03) | -0.23 (-0.26, -0.21) |
|  | Painful Conditions + Cardiovascular and Metabolic Disorders | 10.4 | 0.10 (0.08, 0.11) | 0.23 (0.22, 0.25) | -0.10 (-0.11, -0.09) | -0.05 (-0.06, -0.03) | -0.41 (-0.42, -0.40) |
|  | Hypertension + Other | 8.2 | 0.04 (0.02, 0.05) | 0.15 (0.13, 0.16) | -0.14 (-0.16, -0.12) | 0.03 (0.01, 0.05) | -0.41 (-0.43, -0.40) |
|  | Liver + Alcohol + Dementia | 1.1 | 0.31 (0.27, 0.35) | 0.53 (0.49, 0.57) | -0.12 (-0.16, -0.08) | -0.40 (-0.45, -0.36) | -0.46 (-0.50, -0.42) |
|  | Circulatory | 1.4 | 0.13 (0.09, 0.17) | 0.61 (0.58, 0.65) | -0.34 (-0.38, -0.30) | -0.07 (-0.10, -0.03) | -0.73 (-0.77, -0.69) |
| **WOMEN (N=218,781)^2^** | | | | | | | |
| **Count**  **All** | **2+** | 35.1 | 0.11 (0.10, 0.12) | 0.17 (0.16, 0.18) | -0.11 (-0.12, -0.11) | -0.06 (-0.07, -0.05) | -0.17 (-0.17, -0.16) |
|  | **3+** | 19.0 | 0.14 (0.13, 0.15) | 0.24 (0.23, 0.24) | -0.13 (-0.14, -0.12) | -0.07 (-0.08, -0.06) | -0.21 (-0.22, -0.20) |
| **Count MostPrev** | **2+** | 28.9 | 0.10 (0.09, 0.11) | 0.16 (0.15, 0.17) | -0.11 (-0.12, -0.10) | -0.06 (-0.07, -0.05) | -0.16 (-0.17, -0.15) |
|  | **3+** | 13.3 | 0.13 (0.11, 0.14) | 0.20 (0.19, 0.21) | -0.13 (-0.14, -0.12) | -0.08 (-0.09, -0.07) | -0.19 (-0.20, -0.18) |
| **Count**  **Syst** | **2+** | 33.8 | 0.11 (0.10, 0.12) | 0.17 (0.17, 0.18) | -0.11 (-0.12, -0.10) | -0.06 (-0.07, -0.05) | -0.16 (-0.17, -0.15) |
|  | **3+** | 16.5 | 0.14 (0.13, 0.15) | 0.24 (0.23, 0.25) | -0.12 (-0.13, -0.11) | -0.07 (-0.08, -0.06) | -0.19 (-0.20, -0.18) |
| **Clustering** | Mental Disorders | 1.0 | 0.15 (0.11, 0.19) | 0.27 (0.23, 0.30) | -0.03 (-0.07, 0.01) | -0.16 (-0.20, -0.12) | -0.07 (-0.11, -0.03) |
|  | Respiratory | 1.0 | 0.46 (0.42, 0.50) | 0.34 (0.30, 0.38) | -0.05 (-0.09, -0.01) | -0.09 (-0.13, -0.05) | -0.23 (-0.27, -0.19) |
|  | Digestive | 9.2 | 0.10 (0.09, 0.12) | 0.13 (0.12, 0.15) | -0.18 (-0.19, -0.16) | -0.08 (-0.09, -0.07) | -0.17 (-0.18, -0.15) |
|  | Painful Conditions + Other | 13.6 | 0.03 (0.02, 0.04) | 0.15 (0.14, 0.16) | -0.11 (-0.13, -0.10) | -0.02 (-0.03, -0.01) | -0.17 (-0.18, -0.15) |
|  | Asthma + Other | 5.3 | 0.15 (0.13, 0.17) | 0.10 (0.09, 0.12) | -0.04 (-0.06, -0.02) | -0.09 (-0.11, -0.07) | -0.14 (-0.16, -0.12) |
|  | Connective + Osteoporosis + Neurological | 4.9 | 0.23 (0.22, 0.25) | 0.33 (0.31, 0.35) | -0.11 (-0.13, -0.09) | -0.08 (-0.10, -0.06) | -0.20 (-0.22, -0.18) |

**Abbreviations**: Count MostPrev = 12 most prevalent conditions in the list; Count Syst = count of body-systems involved; 2+ = multimorbidity defined using 2 or more conditions; 3+ = multimorbidity defined using 3 or more conditions; HDL = High-Density Lipoprotein; IGF = Insulin-like Growth Factor; LDL = low-density lipoprotein.

^1^Multimorbidity definitions are based on counting conditions or clustering. Count All, Count MostPrev and Count Syst, count the number of all conditions, 12 most prevalent conditions, or number of body systems involved by conditions, respectively. For count-based approaches, 2+ or 3+ thresholds were used to categorize participants as multimorbid or not. Cluster groups were identified among those multimorbid based on the Count All 2+ Measure.

^2^Analyses based on 405,654 participants (# Men = 186,873, # Women =218,781) with complete data.

^3^Blood-based measures were standardized to have mean 0 and standard deviation equal to 1, separately in men and women.

^4^Associations were computed using linear regression analysis adjusted for age, Townsend deprivation index, ethnicity, and body mass index. Estimates represent the expected change in each blood-based measure among those with the corresponding multimorbid status, as compared to the reference group of those identified as “not multimorbid”.

**Table K. Association of multimorbidity measures and mortality at 3- and 10-years of follow-up**

| **Multimorbidity measures^1^** | | **Prevalence, %** | **Mortality,**  **Odds Ratio (95% Confidence Interval)^2^** | |
| --- | --- | --- | --- | --- |
|  |  |  | **3 years** | **10 years** |
| **MEN (N=215,822), mean (SD) age: 56.7 (8.2) years** | | | | |
| **Count**  **All** | **2+** | 34.0 | 1.94 (1.78, 2.11) | 1.70 (1.64, 1.76) |
|  | **3+** | 18.0 | 2.04 (1.87, 2.22) | 1.86 (1.79, 1.94) |
| **Count MostPrev** | **2+** | 26.4 | 1.74 (1.60, 1.89) | 1.54 (1.49, 1.60) |
|  | **3+** | 11.0 | 1.72 (1.55, 1.90) | 1.62 (1.55, 1.70) |
| **Count**  **Syst** | **2+** | 31.8 | 1.86 (1.71, 2.02) | 1.64 (1.58, 1.70) |
|  | **3+** | 14.7 | 1.96 (1.79, 2.14) | 1.79 (1.71, 1.86) |
| **Clustering** | Mental Disorders | 3.7 | 1.84 (1.51, 2.21) | 1.77 (1.62, 1.92) |
|  | Respiratory | 4.9 | 1.84 (1.57, 2.16) | 1.84 (1.71, 1.97) |
|  | Digestive + Osteoporosis | 4.1 | 2.23 (1.90, 2.60) | 1.59 (1.47, 1.72) |
|  | Painful Conditions + Cardiovascular and Metabolic Disorders | 10.5 | 1.44 (1.26, 1.64) | 1.42 (1.34, 1.50) |
|  | Hypertension + Other | 8.2 | 1.85 (1.63, 2.09) | 1.53 (1.44, 1.62) |
|  | Liver + Alcohol + Dementia | 1.2 | 4.60 (3.73, 5.62) | 3.86 (3.46, 4.30) |
|  | Circulatory | 1.4 | 3.46 (2.82, 4.21) | 3.03 (2.74, 3.34) |
| **WOMEN (N=258,575), mean (SD) age: 56.3 (8.0) years** | | | | |
| **Count**  **All** | **2+** | 35.3 | 1.70 (1.53, 1.89) | 1.61 (1.54, 1.68) |
|  | **3+** | 19.1 | 1.71 (1.53, 1.92) | 1.67 (1.60, 1.75) |
| **Count MostPrev** | **2+** | 29.1 | 1.69 (1.52, 1.88) | 1.49 (1.42, 1.56) |
|  | **3+** | 13.4 | 1.62 (1.43, 1.83) | 1.53 (1.45, 1.61) |
| **Count**  **Syst** | **2+** | 33.9 | 1.69 (1.52, 1.88) | 1.59 (1.52, 1.66) |
|  | **3+** | 16.6 | 1.66 (1.47, 1.86) | 1.65 (1.57, 1.73) |
| **Clustering** | Mental Disorders | 1.1 | 3.83 (2.78, 5.14) | 3.23 (2.79, 3.72) |
|  | Respiratory | 1.0 | 3.26 (2.40, 4.33) | 3.33 (2.93, 3.77) |
|  | Digestive | 9.2 | 1.42 (1.19, 1.67) | 1.29 (1.21, 1.39) |
|  | Painful Conditions + Other | 13.7 | 1.55 (1.34, 1.79) | 1.40 (1.31, 1.48) |
|  | Asthma + Other | 5.3 | 1.35 (1.07, 1.69) | 1.51 (1.38, 1.65) |
|  | Connective + Osteoporosis + Neurological | 4.9 | 2.18 (1.82, 2.61) | 2.19 (2.03, 2.36) |

**Abbreviations**: Count MostPrev = 12 most prevalent conditions in the list; Count Syst = count of body-systems involved; 2+ = multimorbidity defined using 2 or more conditions; 3+ = multimorbidity defined using 3 or more conditions.

^1^Multimorbidity definitions are based on counting conditions or clustering. Count All, Count MostPrev and Count Syst, count the number of all conditions, 12 most prevalent conditions, or number of body systems involved by conditions, respectively. For count-based approaches, 2+ or 3+ thresholds were used to categorize participants as multimorbid or not. Cluster groups were identified among those multimorbid based on the Count All 2+ Measure.

^2^Association between multimorbidity and death at 3 or 10 years were computed using logistic regression adjusted for age, Townsend deprivation index, ethnicity, and body mass index. Odds Ratios represent the odds of dying among those with the corresponding multimorbid status, as compared to the reference group of those identified as “not multimorbid”.

**Table L:** **Association of multimorbidity measures and mortality at 3- and 10-years of follow-up (unadjusted results).**

| **Multimorbidity measures^1^** | | **Prevalence, %** | **Mortality,**  **Odds Ratio (95% Confidence Interval)^2^** | |
| --- | --- | --- | --- | --- |
|  |  |  | **3 years** | **10 years** |
| **MEN (N=215,822), mean (SD) age: 56.7 (8.2) years** | | | | |
| **Count**  **All** | **2+** | 34.0 | 2.67 (2.46, 2.89) | 2.45 (2.37, 2.54) |
|  | **3+** | 18.0 | 2.87 (2.64, 3.11) | 2.73 (2.63, 2.83) |
| **Count MostPrev** | **2+** | 26.4 | 2.32 (2.14, 2.52) | 2.14 (2.06, 2.22) |
|  | **3+** | 11.0 | 2.34 (2.12, 2.57) | 2.27 (2.17, 2.37) |
| **Count**  **Syst** | **2+** | 31.8 | 2.54 (2.34, 2.75) | 2.34 (2.26, 2.42) |
|  | **3+** | 14.7 | 2.73 (2.5, 2.97) | 2.58 (2.48, 2.68) |
| **Clustering** | Mental Disorders | 3.7 | 2.19 (1.81, 2.63) | 2.12 (1.95, 2.3) |
|  | Respiratory | 4.9 | 2.49 (2.12, 2.91) | 2.55 (2.38, 2.73) |
|  | Digestive + Osteoporosis | 4.1 | 3.11 (2.66, 3.62) | 2.33 (2.16, 2.51) |
|  | Painful Conditions + Cardiovascular and Metabolic Disorders | 10.5 | 1.92 (1.69, 2.18) | 1.99 (1.89, 2.1) |
|  | Hypertension + Other | 8.2 | 2.79 (2.47, 3.15) | 2.49 (2.36, 2.63) |
|  | Liver + Alcohol + Dementia | 1.2 | 6.18 (5.02, 7.53) | 5.06 (4.55, 5.61) |
|  | Circulatory | 1.4 | 5.34 (4.37, 6.48) | 4.89 (4.43, 5.38) |
| **WOMEN (N=258,575), mean (SD) age: 56.3 (8.0) years** | | | | |
| **Count**  **All** | **2+** | 35.3 | 2.04 (1.84, 2.26) | 2.07 (1.98, 2.15) |
|  | **3+** | 19.1 | 2.08 (1.86, 2.32) | 2.18 (2.08, 2.28) |
| **Count MostPrev** | **2+** | 29.1 | 2.01 (1.81, 2.22) | 1.9 (1.82, 1.98) |
|  | **3+** | 13.4 | 1.95 (1.72, 2.2) | 1.97 (1.87, 2.07) |
| **Count**  **Syst** | **2+** | 33.9 | 2.02 (1.82, 2.24) | 2.03 (1.95, 2.12) |
|  | **3+** | 16.6 | 2 (1.78, 2.24) | 2.13 (2.03, 2.23) |
| **Clustering** | Mental Disorders | 1.1 | 3.98 (2.9, 5.33) | 3.31 (2.87, 3.8) |
|  | Respiratory | 1.0 | 4.49 (3.31, 5.95) | 4.94 (4.36, 5.58) |
|  | Digestive | 9.2 | 1.74 (1.47, 2.05) | 1.73 (1.62, 1.86) |
|  | Painful Conditions + Other | 13.7 | 1.83 (1.58, 2.1) | 1.76 (1.66, 1.87) |
|  | Asthma + Other | 5.3 | 1.54 (1.22, 1.91) | 1.83 (1.68, 1.99) |
|  | Connective + Osteoporosis + Neurological | 4.9 | 2.8 (2.33, 3.34) | 2.99 (2.77, 3.22) |

**Abbreviations**: Count MostPrev = 12 most prevalent conditions in the list; Count Syst = count of body-systems involved; 2+ = multimorbidity defined using 2 or more conditions; 3+ = multimorbidity defined using 3 or more conditions.

^1^Multimorbidity definitions are based on counting conditions or clustering. Count All, Count MostPrev and Count Syst, count the number of all conditions, 12 most prevalent conditions, or number of body systems involved by conditions, respectively. For count-based approaches, 2+ or 3+ thresholds were used to categorize participants as multimorbid or not. Cluster groups were identified among those multimorbid based on the Count All 2+ Measure.

^2^Association between multimorbidity and death at 3 or 10 years were computed using logistic regression adjusted for age, Townsend deprivation index, ethnicity, and body mass index. Odds Ratios represent the odds of dying among those with the corresponding multimorbid status, as compared to the reference group of those identified as “not multimorbid”.

**Table M. Association of multimorbidity measures and mortality using a time-to-event framework.**

| **Multimorbidity measures^1^** | | **HR**^2^ **(95% CI)**  **Men (N=215,822)** |
| --- | --- | --- |
| **Count All** | **2+** | 1.71 (1.66, 1.75) |
|  | **3+** | 1.81 (1.76, 1.86) |
| **Count MostPrev** | **2+** | 1.56 (1.52, 1.60) |
|  | **3+** | 1.59 (1.54, 1.64) |
| **Count Syst** | **2+** | 1.65 (1.61, 1.69) |
|  | **3+** | 1.73 (1.68, 1.78) |
| **Clustering** | Mental Disorders | 1.79 (1.69, 1.90) |
|  | Respiratory | 1.92 (1.83, 2.01) |
|  | Digestive + Osteoporosis | 1.57 (1.49, 1.66) |
|  | Painful Conditions + Cardiovascular and Metabolic Disorders | 1.54 (1.48, 1.60) |
|  | Hypertension + Other | 1.50 (1.44, 1.56) |
|  | Liver + Alcohol + Dementia | 3.24 (3.00, 3.50) |
|  | Circulatory | 2.72 (2.54, 2.91) |
| **Multimorbidity measures^1^** | | **HR**^2^ **(95% CI)**  **Women (N=258,575)** |
| **Count All** | **2+** | 1.64 (1.59, 1.69) |
|  | **3+** | 1.69 (1.64, 1.75) |
| **Count MostPrev** | **2+** | 1.51 (1.46, 1.56) |
|  | **3+** | 1.55 (1.50, 1.61) |
| **Count Syst** | **2+** | 1.62 (1.57, 1.67) |
|  | **3+** | 1.66 (1.61, 1.72) |
| **Clustering** | Mental Disorders | 3.09 (2.79, 3.43) |
|  | Respiratory | 3.15 (2.89, 3.44) |
|  | Digestive | 1.38 (1.31, 1.45) |
|  | Painful Conditions + Other | 1.43 (1.37, 1.49) |
|  | Asthma + Other | 1.57 (1.48, 1.67) |
|  | Connective + Osteoporosis + Neurological | 2.17 (2.06, 2.28) |

**Abbreviations**: Count MostPrev = 12 most prevalent conditions in the list; Count Syst = count of body-systems involved; 2+ = multimorbidity defined using 2 or more conditions; 3+ = multimorbidity defined using 3 or more conditions; HR: Hazard Ratio; CI: Confidence Interval

^1^Multimorbidity definitions are based on counting conditions or clustering. Count All, Count MostPrev and Count Syst, count the number of all conditions, 12 most prevalent conditions, or number of body systems involved by conditions, respectively. For count-based approaches, 2+ or 3+ thresholds were used to categorize participants as multimorbid or not. Cluster groups were identified among those multimorbid based on the Count All 2+ Measure.

^2^Association between multimorbidity and death were computed using Cox models adjusted for age, Townsend deprivation index, ethnicity, and body mass index.

**Supplementary References**

1. Ronaldson A, Arias de la Torre J, Ashworth M, et al. Associations between air pollution and multimorbidity in the UK Biobank: A cross-sectional study. *Front Public Health* 2022; **10**: 1035415.

**List of titles and legends**

**Supplementary Material Table A. List of chronic conditions and corresponding ICD-10 codes used to define multimorbidity indicators**

Abbreviation: ICD: International Classification of Diseases; ICD-10 codes for chronic conditions were based on the list of codes used to define these conditions in Ronaldson et al.[25]

**Supplementary Material Table B. Chronic conditions used in multimorbidity definitions**

Abbreviation: ICD: International Classification of Diseases

**Supplementary Material Table C.** **Descriptive data (% (N) or Mean (SD)) on covariates and concurrent health outcomes in included( N=474,397) and excluded (N=5815) participants.**

Abbreviation: SD: standard deviation; ^1^Mean (Standard Deviation) reported for these measure

**Supplementary Material Table D.** **Descriptive data (% (N) or Mean (SD)) on covariates and concurrent health outcomes**

^1^Mean (Standard Deviation) reported for these measure

**Supplementary Material Table E.**  **Descriptive data (% (N) or Mean (SD)) on concurrent health outcomes and mortality as a function of multimorbidity measures in MEN**

**Abbreviations**: Count MostPrev = 12 most prevalent conditions in the list; Count Syst = count of body-systems involved; 2+ = multimorbidity defined using 2 or more conditions; 3+ = multimorbidity defined using 3 or more conditions.

^1^Multimorbidity definitions are based on counting conditions or clustering. Count All, Count MostPrev and Count Syst, count the number of all conditions, 12 most prevalent conditions, or number of body systems involved by conditions, respectively. For count-based approaches, 2+ or 3+ thresholds were used to categorize participants as multimorbid or not. Cluster groups were identified among those multimorbid based on the Count All 2+ Measure.

^2^Mean (SD) reported for these metrics

^3^The condition count is reported using the respective list for each method

**Supplementary Material Table F.** **Descriptive data (% (N) or Mean (SD)) on concurrent health outcomes and mortality as a function of multimorbidity measures in WOMEN**

**Abbreviations**: Count MostPrev = 12 most prevalent conditions in the list; Count Syst = count of body-systems involved; 2+ = multimorbidity defined using 2 or more conditions; 3+ = multimorbidity defined using 3 or more conditions.

^1^Multimorbidity definitions are based on counting conditions or clustering. Count All, Count MostPrev and Count Syst, count the number of all conditions, 12 most prevalent conditions, or number of body systems involved by conditions, respectively. For count-based approaches, 2+ or 3+ thresholds were used to categorize participants as multimorbid or not. Cluster groups were identified among those multimorbid based on the Count All 2+ Measure.

^2^Mean (SD) reported for these metrics

^3^The condition count is reported using the respective list for each method

**Supplementary Material Table G. Multimorbidity prevalence, overall and by age groups, defined using terciles, in men and women.**

**Abbreviations**: Count MostPrev = 12 most prevalent conditions in the list; Count Syst = count of body-systems involved; 2+ = multimorbidity defined using 2 or more conditions; 3+ = multimorbidity defined using 3 or more conditions

^1^Multimorbidity definitions are based on counting conditions or clustering. Count All, Count MostPrev and Count Syst, count the number of all conditions, 12 most prevalent conditions, or number of body systems involved by conditions, respectively. For count-based approaches, 2+ or 3+ thresholds were used to categorize participants as multimorbid or not. Cluster groups were identified among those multimorbid based on the Count All: 2+ Measure

**Supplementary Material Table H:** **Cross-sectional associations of multimorbidity measures with health outcomes in men and women (unadjusted results).**

**Abbreviations**: Count MostPrev = 12 most prevalent conditions in the list; Count Syst = count of body-systems involved; 2+ = multimorbidity defined using 2 or more conditions; 3+ = multimorbidity defined using 3 or more conditions

**^1^**Multimorbidity definitions are based on counting conditions or clustering. Count All, Count MostPrev and Count Syst count the number of all conditions, 12 most prevalent conditions or number of body-systems affected, respectively. Cluster groups were identified among those multimorbid based on the Count All: 2+ Measure.

**^2^**Logistic regression adjusted for age, Townsend deprivation index, ethnicity, and body mass index. Odds Ratios represent the odds of a health outcome among those with the corresponding multimorbid status, as compared to the reference group of those identified as “not multimorbid”.

**Supplementary Material Table I. Standardized mean value of blood-based measures as a function of multimorbidity measures**

**Abbreviations**: Count MostPrev = 12 most prevalent conditions in the list; Count Syst = count of body-systems involved; 2+ = multimorbidity defined using 2 or more conditions; 3+ = multimorbidity defined using 3 or more conditions; HDL = High-Density Lipoprotein; IGF = Insulin-like Growth Factor; LDL = low-density lipoprotein.

^1^Multimorbidity definitions are based on counting conditions or clustering. Count All, Count MostPrev and Count Syst, count the number of all conditions, 12 most prevalent conditions, or number of body systems involved by conditions, respectively. For count-based approaches, 2+ or 3+ thresholds were used to categorize participants as multimorbid or not. Cluster groups were identified among those multimorbid based on the Count All 2+ Measure.

^2^These analyses were completed among all 405,654 participants (# Men = 186,873, # Women =218,781) with fully observed biomarker data

^3^Blood-based measures were standardized to have mean 0 and standard deviation equal to 1, separately in men and women.

**Supplementary Material Table J.** **Cross-sectional association of** **multimorbidity measures with blood-based measures**

**Abbreviations**: Count MostPrev = 12 most prevalent conditions in the list; Count Syst = count of body-systems involved; 2+ = multimorbidity defined using 2 or more conditions; 3+ = multimorbidity defined using 3 or more conditions; HDL = High-Density Lipoprotein; IGF = Insulin-like Growth Factor; LDL = low-density lipoprotein.

^1^Multimorbidity definitions are based on counting conditions or clustering. Count All, Count MostPrev and Count Syst, count the number of all conditions, 12 most prevalent conditions, or number of body systems involved by conditions, respectively. For count-based approaches, 2+ or 3+ thresholds were used to categorize participants as multimorbid or not. Cluster groups were identified among those multimorbid based on the Count All 2+ Measure.

^2^Analyses based on 405,654 participants (# Men = 186,873, # Women =218,781) with complete data.

^3^Blood-based measures were standardized to have mean 0 and standard deviation equal to 1, separately in men and women.

^4^Associations were computed using linear regression analysis adjusted for age, Townsend deprivation index, ethnicity, and body mass index. Estimates represent the expected change in each blood-based measure among those with the corresponding multimorbid status, as compared to the reference group of those identified as “not multimorbid”.

**Supplementary Material Table K. Association of multimorbidity measures and mortality at 3- and 10-years of follow-up**

**Abbreviations**: Count MostPrev = 12 most prevalent conditions in the list; Count Syst = count of body-systems involved; 2+ = multimorbidity defined using 2 or more conditions; 3+ = multimorbidity defined using 3 or more conditions.

^1^Multimorbidity definitions are based on counting conditions or clustering. Count All, Count MostPrev and Count Syst, count the number of all conditions, 12 most prevalent conditions, or number of body systems involved by conditions, respectively. For count-based approaches, 2+ or 3+ thresholds were used to categorize participants as multimorbid or not. Cluster groups were identified among those multimorbid based on the Count All 2+ Measure.

^2^Association between multimorbidity and death at 3 or 10 years were computed using logistic regression adjusted for age, Townsend deprivation index, ethnicity, and body mass index. Odds Ratios represent the odds of dying among those with the corresponding multimorbid status, as compared to the reference group of those identified as “not multimorbid”.

**Supplementary Material Table L:** **Association of multimorbidity measures and mortality at 3- and 10-years of follow-up (unadjusted results).**

**Abbreviations**: Count MostPrev = 12 most prevalent conditions in the list; Count Syst = count of body-systems involved; 2+ = multimorbidity defined using 2 or more conditions; 3+ = multimorbidity defined using 3 or more conditions.

^1^Multimorbidity definitions are based on counting conditions or clustering. Count All, Count MostPrev and Count Syst, count the number of all conditions, 12 most prevalent conditions, or number of body systems involved by conditions, respectively. For count-based approaches, 2+ or 3+ thresholds were used to categorize participants as multimorbid or not. Cluster groups were identified among those multimorbid based on the Count All 2+ Measure.

^2^Association between multimorbidity and death at 3 or 10 years were computed using logistic regression adjusted for age, Townsend deprivation index, ethnicity, and body mass index. Odds Ratios represent the odds of dying among those with the corresponding multimorbid status, as compared to the reference group of those identified as “not multimorbid”.

**Supplementary Material Table M. Association of multimorbidity measures and mortality using a time-to-event framework.**

**Abbreviations**: Count MostPrev = 12 most prevalent conditions in the list; Count Syst = count of body-systems involved; 2+ = multimorbidity defined using 2 or more conditions; 3+ = multimorbidity defined using 3 or more conditions; HR: Hazard Ratio; CI: Confidence Interval

^1^Multimorbidity definitions are based on counting conditions or clustering. Count All, Count MostPrev and Count Syst, count the number of all conditions, 12 most prevalent conditions, or number of body systems involved by conditions, respectively. For count-based approaches, 2+ or 3+ thresholds were used to categorize participants as multimorbid or not. Cluster groups were identified among those multimorbid based on the Count All 2+ Measure.

^2^Association between multimorbidity and death were computed using Cox models adjusted for age, Townsend deprivation index, ethnicity, and body mass index.
