# Supplementary material for: Comparison of count-based and clustering definitions of multimorbidity and their association with prevalence of multimorbidity, health profiles, and mortality: A cohort study of UK Biobank participants
Source: PLoS Med. 2026 Jun 12;23(6):e1004914. doi: 10.1371/journal.pmed.1004914 (PMC13309015; doi:10.1371/journal.pmed.1004914)
Supplement: S1 Checklist — Available at https://www.strobe-statement.org/checklists/. (DOCX) [file pmed.1004914.s002.docx]

**Comparison of count-based and clustering definitions of multimorbidity and their association with prevalence of multimorbidity, health profiles, and mortality: A cohort study of UK Biobank participants**

Gabriella C SILVA, PhD^1^; Aurore FAYOSSE, MSc^1^; Louis JACOB, MD, PhD^1,2,3^; Séverine SABIA, PhD^1, 4^; Archana SINGH-MANOUX, PhD^1, 4^; Benjamin LANDRÉ, PhD*^1^

1 Université Paris Cité, Inserm U1153, CRESS, Epidemiology of Ageing and Neurodegenerative diseases, Paris, France

2 Department of Physical Medicine and Rehabilitation, Université Paris Cité, AP-HP, Lariboisière-Fernand Widal Hospital, Paris 75010, France

3 Research and Development Unit, Parc Sanitari Sant Joan de Déu, CIBERSAM, ISCIII, Dr. Antoni Pujadas, 42, Sant Boi de Llobregat, Barcelona 08030, Spain

4 Faculty of Brain Sciences, University College London, London, UK

*Address for correspondence

Université Paris Cité

Inserm U1153, Epidemiology of Ageing and Neurodegenerative diseases

10 Avenue de Verdun, 75010 Paris, France

Email: [benjamin.landre@inserm.fr](mailto:gabriella.silva@inserm.fr)

**Checklist**

**S1 STROBE checklist:**

|  | Item No. | Recommendation | Page  No. |
| --- | --- | --- | --- |
| **Title and abstract** | 1 | (*a*) Indicate the study’s design with a commonly used term in the title or the abstract | Title page |
|  |  | (*b*) Provide in the abstract an informative and balanced summary of what was done and what was found | Abstract |
| Introduction | | | |
| Background/rationale | 2 | Explain the scientific background and rationale for the investigation being reported | Introduction, paragraphs 1-5 |
| Objectives | 3 | State specific objectives, including any prespecified hypotheses | Introduction, paragraph 6 |
| Methods | | | |
| Study design | 4 | Present key elements of study design early in the paper | Title, abstract, method paragraph 1 |
| Setting | 5 | Describe the setting, locations, and relevant dates, including periods of recruitment, exposure, follow-up, and data collection | Method paragraph 1 |
| Participants | 6 | (*a*) *Cohort study*—Give the eligibility criteria, and the sources and methods of selection of participants. Describe methods of follow-up  *Case-control study*—Give the eligibility criteria, and the sources and methods of case ascertainment and control selection. Give the rationale for the choice of cases and controls  *Cross-sectional study*—Give the eligibility criteria, and the sources and methods of selection of participants | Method paragraph 1  Results paragraph 1  Figure 1 |
|  |  | (*b*) *Cohort study*—For matched studies, give matching criteria and number of exposed and unexposed  *Case-control study*—For matched studies, give matching criteria and the number of controls per case |  |
| Variables | 7 | Clearly define all outcomes, exposures, predictors, potential confounders, and effect modifiers. Give diagnostic criteria, if applicable | Method, paragraphs 2 to 4. |
| Data sources/ measurement | 8* | For each variable of interest, give sources of data and details of methods of assessment (measurement). Describe comparability of assessment methods if there is more than one group |  |
| Bias | 9 | Describe any efforts to address potential sources of bias | Methods paragraphs 2-5 |
| Study size | 10 | Explain how the study size was arrived at | Results, paragraph 1  Figure 1: flow chart for study population. |

| Quantitative variables | 11 | Explain how quantitative variables were handled in the analyses. If applicable, describe which groupings were chosen and why | Methods paragraphs 2-6 |
| --- | --- | --- | --- |
| Statistical methods | 12 | (*a*) Describe all statistical methods, including those used to control for confounding | Methods, paragraphs 6-8. |
|  |  | (*b*) Describe any methods used to examine subgroups and interactions |  |
|  |  | (*c*) Explain how missing data were addressed |  |
|  |  | (*d*) *Cohort study*—If applicable, explain how loss to follow-up was addressed  *Case-control study*—If applicable, explain how matching of cases and controls was addressed  *Cross-sectional study*—If applicable, describe analytical methods taking account of sampling strategy | Not relevant |
|  |  | (*e*) Describe any sensitivity analyses | Methods paragraph 8 |
| Participants | 13* | (a) Report numbers of individuals at each stage of study—eg numbers potentially eligible, examined for eligibility, confirmed eligible, included in the study, completing follow-up, and analysed | Results, paragraph 1  Figure 1: flow chart for study population. |
|  |  | (b) Give reasons for non-participation at each stage |  |
|  |  | (c) Consider use of a flow diagram |  |
| Descriptive data | 14* | (a) Give characteristics of study participants (eg demographic, clinical, social) and information on exposures and potential confounders | Results, paragraph 1-2 |
|  |  | (b) Indicate number of participants with missing data for each variable of interest |  |
|  |  | (c) *Cohort study*—Summarise follow-up time (eg, average and total amount) |  |
| Outcome data | 15* | *Cohort study*—Report numbers of outcome events or summary measures over time |  |
|  |  | *Case-control study—*Report numbers in each exposure category, or summary measures of exposure |  |
|  |  | *Cross-sectional study—*Report numbers of outcome events or summary measures |  |
| Main results | 16 | (*a*) Give unadjusted estimates and, if applicable, confounder-adjusted estimates and their precision (eg, 95% confidence interval). Make clear which confounders were adjusted for and why they were included | Results, paragraphs 4-end of results |
|  |  | (*b*) Report category boundaries when continuous variables were categorized | NR |
|  |  | (*c*) If relevant, consider translating estimates of relative risk into absolute risk for a meaningful time period |  |

| Other analyses | 17 | Report other analyses done—eg analyses of subgroups and interactions, and sensitivity analyses | Results, last paragraph.  Supplementary table S11 |
| --- | --- | --- | --- |
| Key results | 18 | Summarise key results with reference to study objectives | Discussion paragraph 1 |
| Limitations | 19 | Discuss limitations of the study, taking into account sources of potential bias or imprecision. Discuss both direction and magnitude of any potential bias | Discussion paragraph 6 |
| Interpretation | 20 | Give a cautious overall interpretation of results considering objectives, limitations, multiplicity of analyses, results from similar studies, and other relevant evidence | Discussion, last paragraph |
| Generalisability | 21 | Discuss the generalisability (external validity) of the study results | Discussion, paragraph 4-5 |
| Other information | |  |  |
| Funding | 22 | Give the source of funding and the role of the funders for the present study and, if applicable, for the original study on which the present article is based | Competing interests, acknowledgment, role of the funding source |

Strobe checklist is available at https://www.strobe-statement.org/checklists/
