# Supplementary material for: Comparison of count-based and clustering definitions of multimorbidity and their association with prevalence of multimorbidity, health profiles, and mortality: A cohort study of UK Biobank participants
Source: PLoS Med. 2026 Jun 12;23(6):e1004914. doi: 10.1371/journal.pmed.1004914 (PMC13309015; doi:10.1371/journal.pmed.1004914)
Supplement: S1 Methods — Including: Method A: methods used for cluster analyses. Method B: Men, step 1. Selecting the optimal number of clusters among 73,362 multimorbid men using various clustering metrics. Method C: Men, step 2. Labeling and summarizing the clusters. Method D: Table. Summary values at the optimal number of clusters in men. Method E: Figure. Prevalence, Observed/Expected Ratio, Exclusivity for the seven clusters in men. Method F: Figure. Ten most frequent combinations of conditions within each cluster in men. Method G: WOMEN, Step 1. Selecting the optimal number of clusters among 91,261 multimorbid women using various clustering metrics. Method H: WOMEN, Step 2. Labeling and summarizing the clusters. Method I: Table. Summary values at the optimal number of clusters in women. Method J: Figure. Prevalence, Observed/Expected Ratio, Exclusivity for the six clusters in women. Method K: Figure. Ten most frequent combinations of conditions within each cluster in women. (DOCX) [file pmed.1004914.s003.docx]

**Comparison of count-based and clustering definitions of multimorbidity and their association with prevalence of multimorbidity, health profiles, and mortality: A cohort study of UK Biobank participants**

Gabriella C SILVA, PhD^1^; Aurore FAYOSSE, MSc^1^; Louis JACOB, MD, PhD^1,2,3^; Séverine SABIA, PhD^1, 4^; Archana SINGH-MANOUX, PhD^1, 4^; Benjamin LANDRÉ, PhD*^1^

1 Université Paris Cité, Inserm U1153, CRESS, Epidemiology of Ageing and Neurodegenerative diseases, Paris, France

2 Department of Physical Medicine and Rehabilitation, Université Paris Cité, AP-HP, Lariboisière-Fernand Widal Hospital, Paris 75010, France

3 Research and Development Unit, Parc Sanitari Sant Joan de Déu, CIBERSAM, ISCIII, Dr. Antoni Pujadas, 42, Sant Boi de Llobregat, Barcelona 08030, Spain

4 Faculty of Brain Sciences, University College London, London, UK

*Address for correspondence

Université Paris Cité

Inserm U1153, Epidemiology of Ageing and Neurodegenerative diseases

10 Avenue de Verdun, 75010 Paris, France

Email: [benjamin.landre@inserm.fr](mailto:gabriella.silva@inserm.fr)

**Supplementary material: cluster analyses**

**Methods A: Methods used for cluster analyses**

We conducted the cluster analyses using the method described by Violan and colleagues^1^ separately in men and women and on participants with two or more conditions from the full list of 38 chronic conditions.

We first applied multiple correspondence analysis to the dataset of 37 binary indicators of chronic conditions (*Endometriosis* was removed for the analyses on men and *Prostate Conditions* in women). We then used the summary from the scree plot to determine that six dimensions were sufficient to summarize the data for both men and women. We then applied k-means clustering to these six dimensions. Due to the highly subjective nature of selecting the optimal number of clusters, we combined multiple metrics to determine potential values for the optimal number of clusters. In men, this corresponded to 5, 6, 7 using the Elbow Method and 13 using the Davies-Bouldin Index. In women, 5, 6 or 7 seemed to be the optimal value according to the Elbow Method while the Davies-Bouldin Index identified 6 as the optimal value. For each of these potential values, we calculated the Jaccard similarities using a bootstrap technique, as in Violan et al.^1^ The authors specify that highly stable clusters should have average Jaccard similarities of at least 0.85. Using this value as a benchmark, we also examined which additional clusters would be identified by increasing the number of clusters. The final decision on the number of clusters was 7 in men and 6 in women.

We then used plots to examine the prevalence (the proportion of people in the cluster with the condition), observed/expected ratio (the proportion of the number of people with the condition in the cluster divided by the expected number of people with that condition if these were equally distributed among the clusters) and exclusivity (the proportion of people with the condition in a specific cluster) for each cluster to meaningfully label the clusters. We also examined the most commonly occurring combinations of conditions within each cluster. We found that for men the 7 multimorbidity clusters were: mental disorders, respiratory, digestive + osteoporosis, painful conditions + cardiovascular and metabolic disorders, hypertension + other, liver + alcohol + dementia, and circulatory conditions. For women, these were: mental disorders, respiratory, digestive, painful conditions + other, asthma + other, and connective + osteoporosis + neurological.

The section below provides the results of these analyses in a step-wise manner, first for men and then for women.

**Supplementary References**

^1^ Violan C, Roso-Llorach A, Foguet-Boreu Q, et al. Multimorbidity patterns with K-means nonhierarchical cluster analysis. *BMC Fam Pract* 2018; **19**(1): 108.

**Methods B: MEN, Step 1.** Selecting the optimal number of clusters among 73,362 multimorbid men using various clustering metrics

| **Number of Clusters** | **Total Within Sum of Squares** | **Davies Bouldin Index** | **Jaccard Similarities** |
| --- | --- | --- | --- |
| 2 | 262127153.91 | 1.89 |  |
| 3 | 224367410.00 | 1.61 |  |
| 4 | 193891351.93 | 1.43 |  |
| 5 | 167160379.05 | 1.39 | 0.995, 0.997, 0.998, 0.992, 0.995 |
| 6 | 150356216.74 | 1.33 | 0.995, 0.988, 0.949, 0.987, 0.972, 0.995 |
| 7 | 140184342.78 | 1.35 | 0.973, 0.995, 0.933, 0.902, 0.995, 0.985, 0.978 |
| 8 | 131141557.67 | 1.36 |  |
| 9 | 123623658.49 | 1.39 |  |
| 10 | 116174161.49 | 1.34 |  |
| 11 | 110866375.20 | 1.36 |  |
| 12 | 106609356.04 | 1.35 |  |
| 13 | 102391738.77 | **1.32** | 0.831, 0.789, 0.745, 0.789, 0.541, 0.975, 0.370, 0.869, 0.745, 0.863, 0.865, 0.540, 0.468 |
| 14 | 99070765.41 | 1.40 |  |
| 15 | 95348194.10 | 1.37 |  |
| 16 | 92302484.70 | 1.36 |  |
| 17 | 89641116.89 | 1.37 |  |
| 18 | 87084682.67 | 1.40 |  |
| 19 | 84766423.65 | 1.41 |  |
| 20 | 82615952.85 | 1.42 |  |


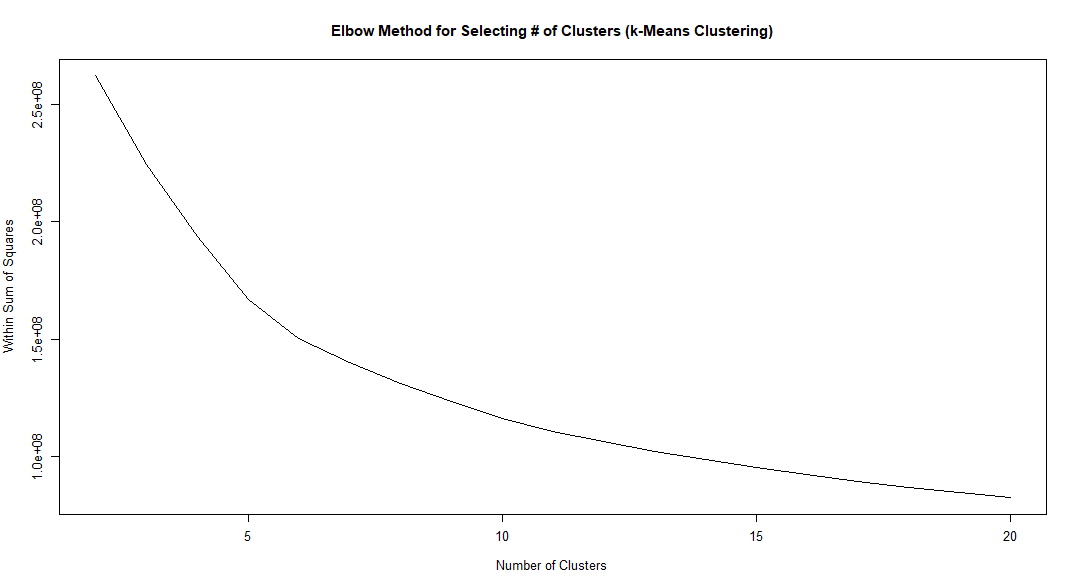


**Methods C: MEN, Step 2. Labeling and summarizing the clusters**

**Figure.** Prevalence, Observed/Expected Ratio, Exclusivity and corresponding labels for the seven clusters in men


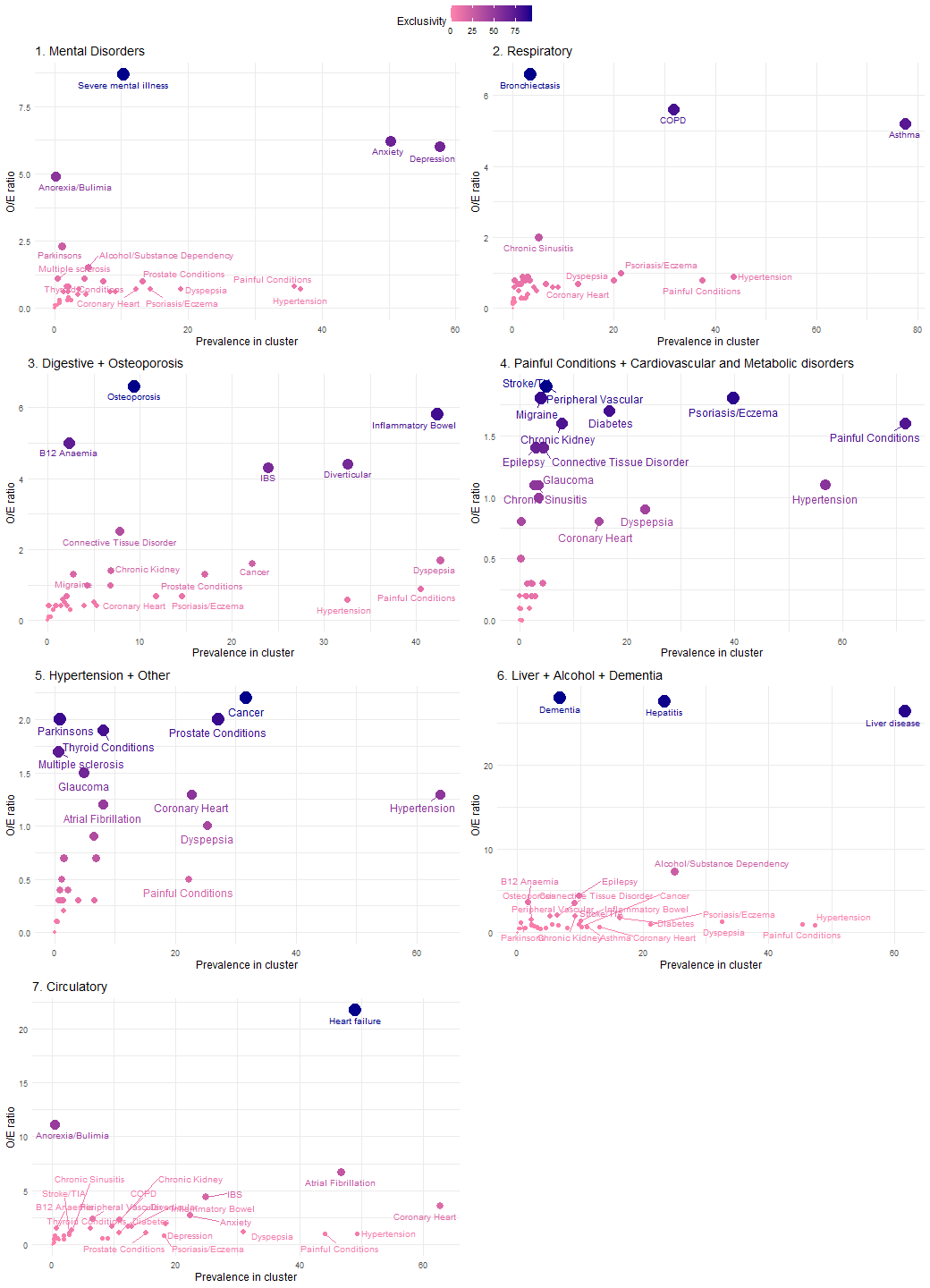


**Methods D: Table.** Summary values at the optimal number of clusters in men

|  | **Number of men per cluster** | **Median number of conditions** |
| --- | --- | --- |
| 1. Mental Disorders | 7,994 | 3 |
| 2. Respiratory | 10,490 | 3 |
| 3. Digestive + Osteoporosis | 8,929 | 3 |
| 4. Painful Conditions + Cardiovascular and Metabolic disorders | 22,605 | 2 |
| 5. Hypertension + Other | 17,769 | 2 |
| 6. Liver + Alcohol + Dementia | 2,518 | 4 |
| 7. Circulatory | 3,057 | 5 |

**Methods E: Figure.** Prevalence, Observed/Expected Ratio, Exclusivity for the seven clusters in men

*
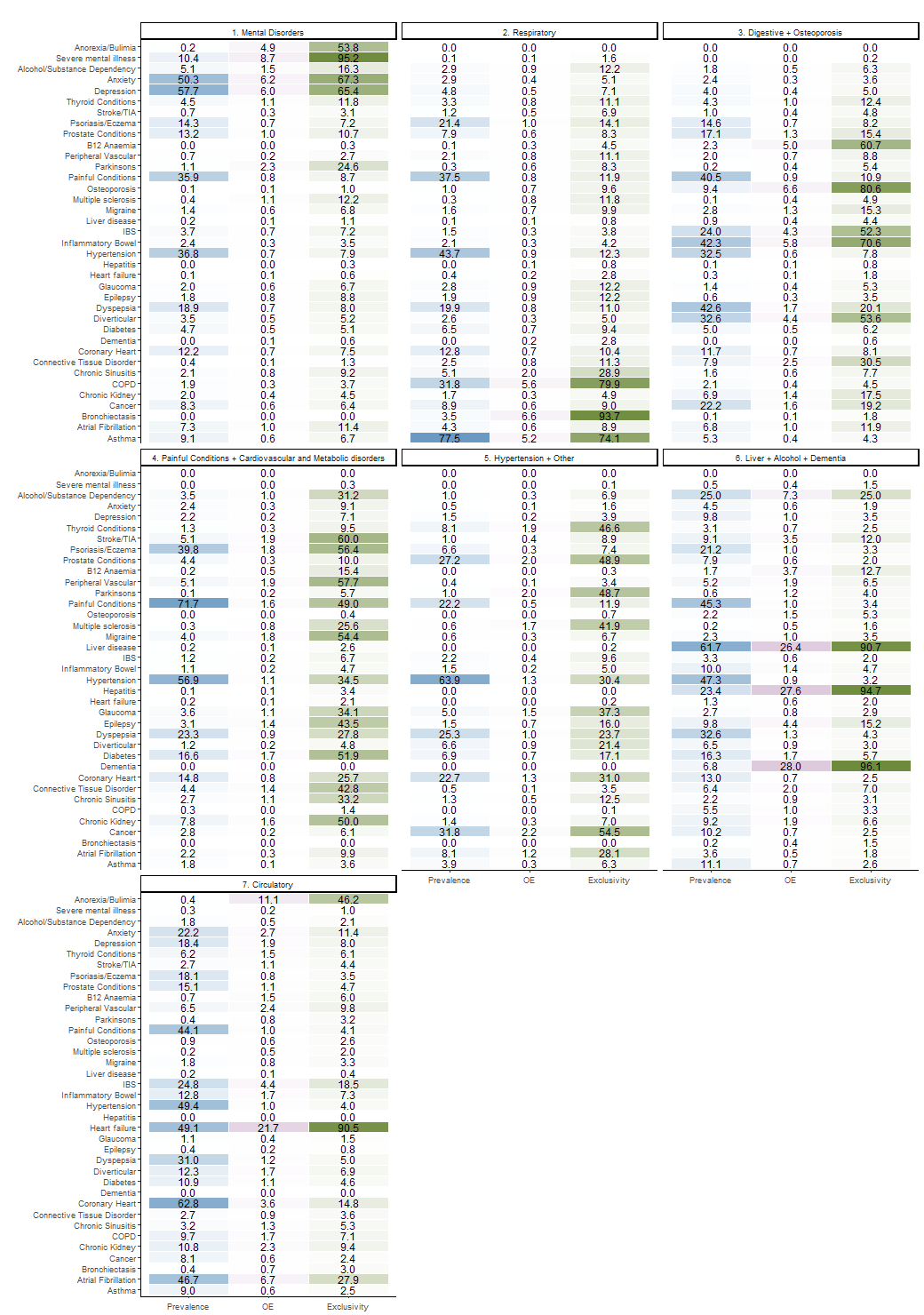
*

**Methods F: Figure.** 10 most frequent combinations of conditions within each cluster in men

**
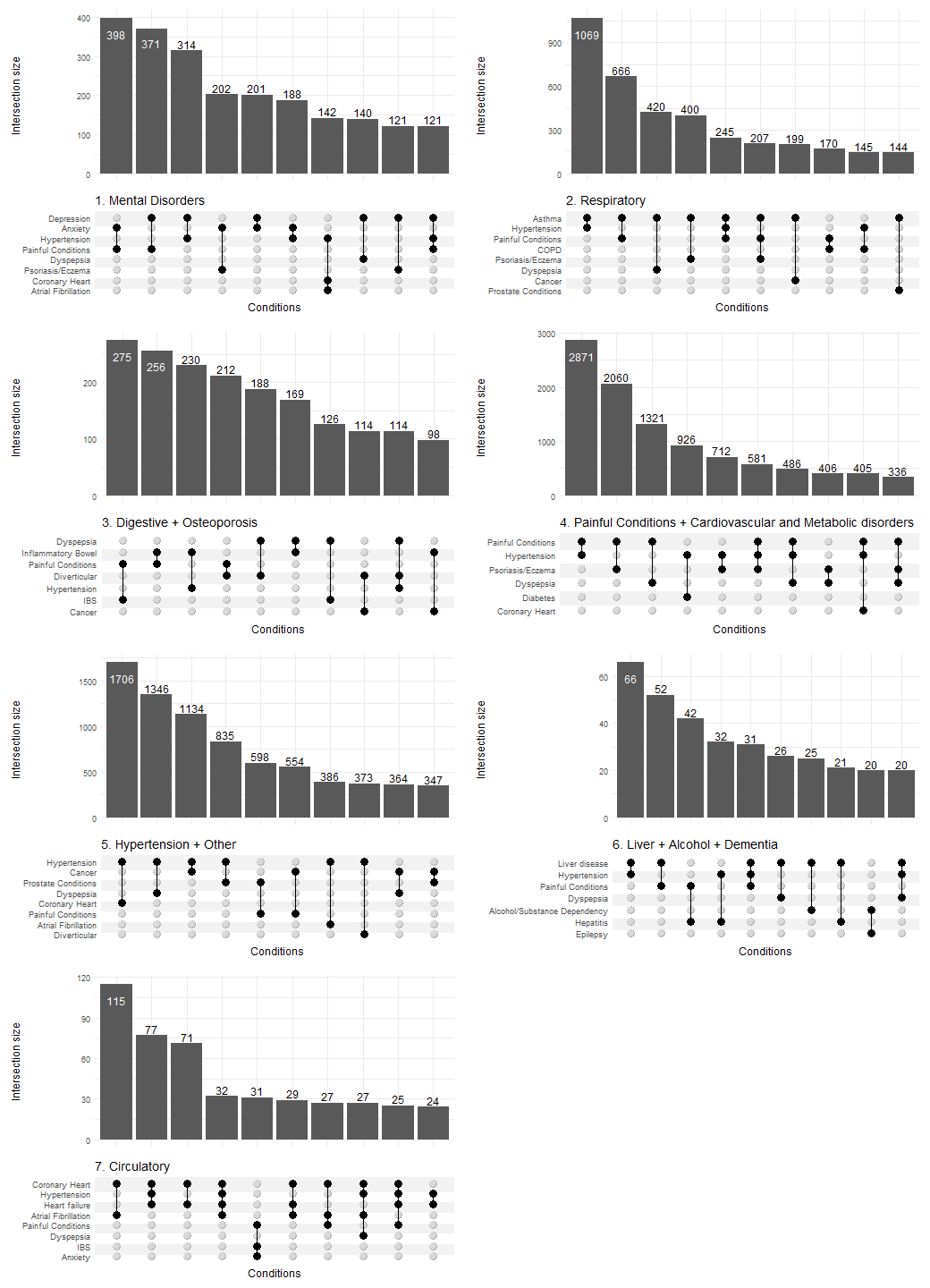
**

**Methods G: WOMEN, Step 1.** Selecting the optimal number of clusters among 91,261 multimorbid women using various clustering metrics

| **Number of Clusters** | **Total Within Sum of Squares** | **Davies Bouldin Index** | **Jaccard Similarities** |
| --- | --- | --- | --- |
| 2 | 395981642.93 | 1.42 |  |
| 3 | 329269681.53 | 1.66 |  |
| 4 | 273732643.25 | 1.40 |  |
| 5 | 250712300.95 | 1.27 | 0.987, 0.997, 0.978, 0.993, 0.997 |
| 6 | 230830637.54 | **1.26** | 0.996, 0.986, 0.999, 0.997, 0.999, 0.997 |
| 7 | 213281636.14 | 1.37 | 0.981, 0.996, 0.987, 0.999, 0.983, 0.993, 0.997 |
| 8 | 198728064.04 | 1.42 |  |
| 9 | 187040422.01 | 1.45 |  |
| 10 | 176532837.29 | 1.43 |  |
| 11 | 168914557.35 | 1.47 |  |
| 12 | 162272434.18 | 1.45 |  |
| 13 | 156361243.31 | 1.45 |  |
| 14 | 150497405.93 | 1.41 |  |
| 15 | 145268959.51 | 1.42 |  |
| 16 | 140763976.09 | 1.39 |  |
| 17 | 136563134.48 | 1.38 |  |
| 18 | 132577133.80 | 1.37 |  |
| 19 | 128906740.98 | 1.36 |  |
| 20 | 126934363.14 | 1.36 |  |


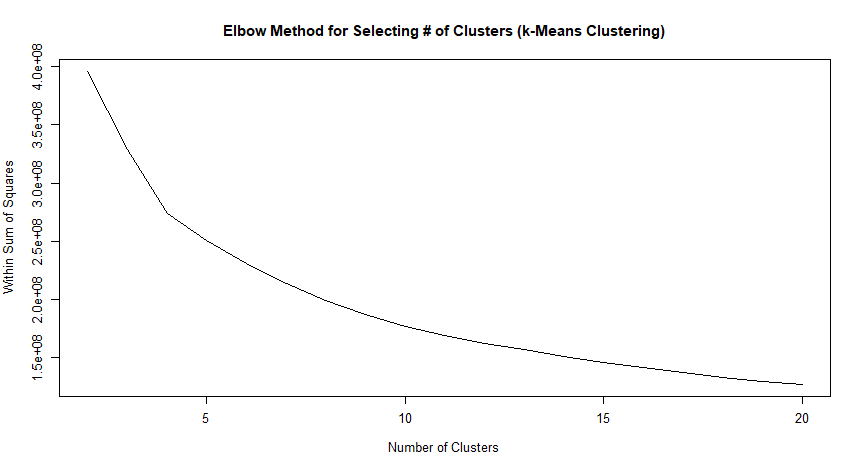


**Methods H: WOMEN, Step 2. Labeling and summarizing the clusters**

**Figure.** Prevalence, Observed/Expected ratio, Exclusivity and corresponding labels for the six clusters in women


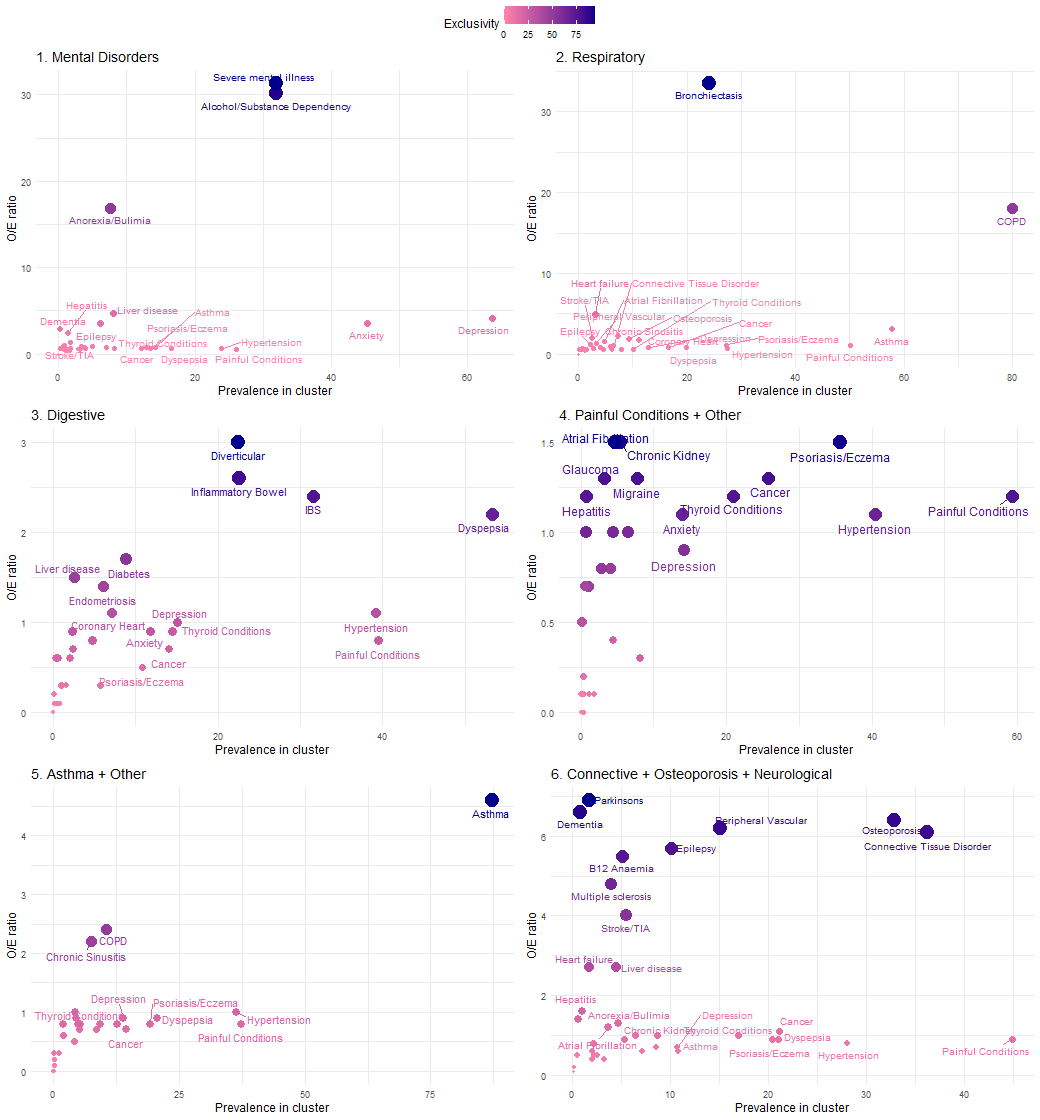


|  | **Number of women per cluster** | **Median number of conditions** |
| --- | --- | --- |
| 1. Mental Disorders | 2,768 | 3 |
| 2. Respiratory | 2,680 | 4 |
| 3. Digestive | 23,868 | 3 |
| 4. Painful Conditions + Other | 35,516 | 2 |
| 5. Asthma + Other | 13,721 | 3 |
| 6. Connective + Osteoporosis + Neurological | 12,708 | 3 |

**Methods I: Table.** Summary values at the optimal number of clusters in women

**Methods J: Figure.** Prevalence, Observed/Expected Ratio, Exclusivity for the six clusters in women


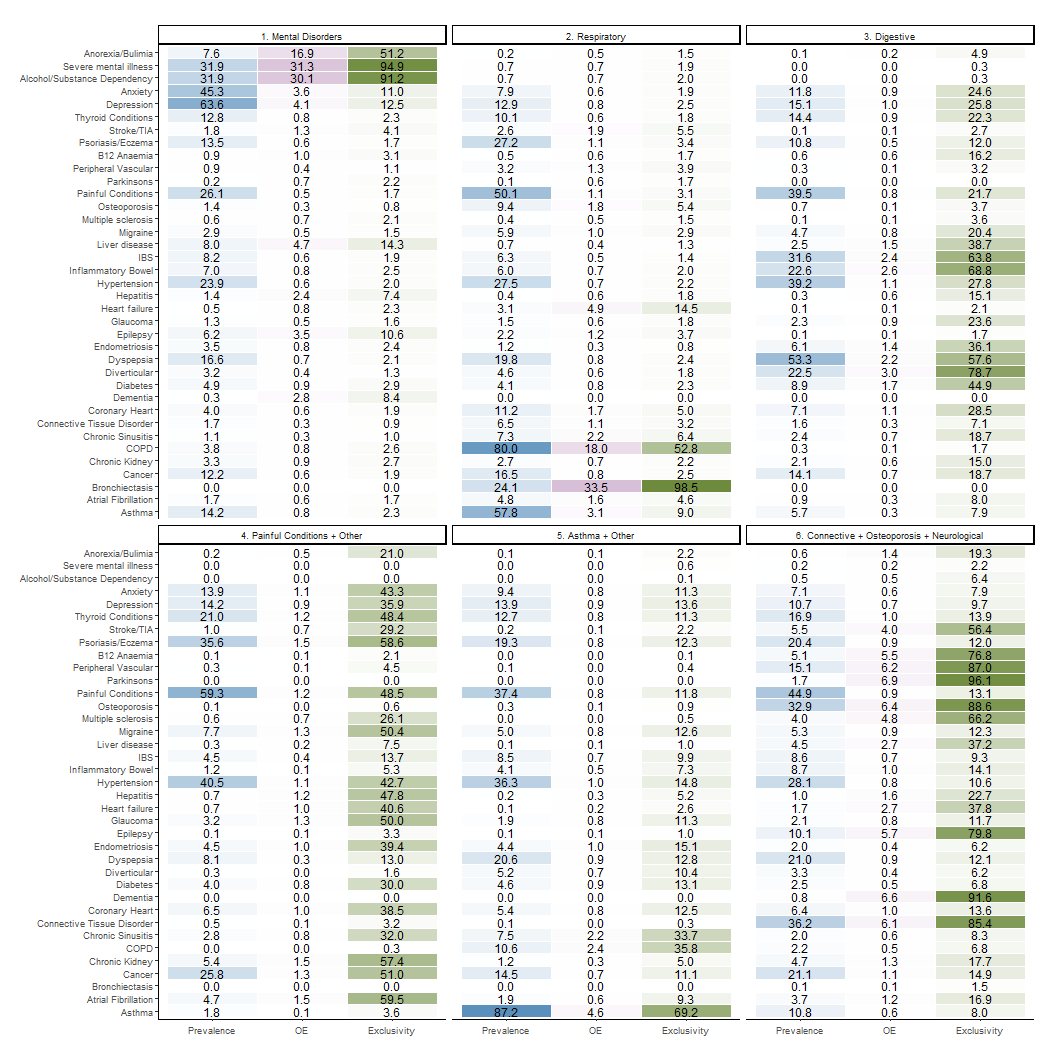


**Methods K: Figure.** 10 most frequent combinations of conditions within each cluster in women


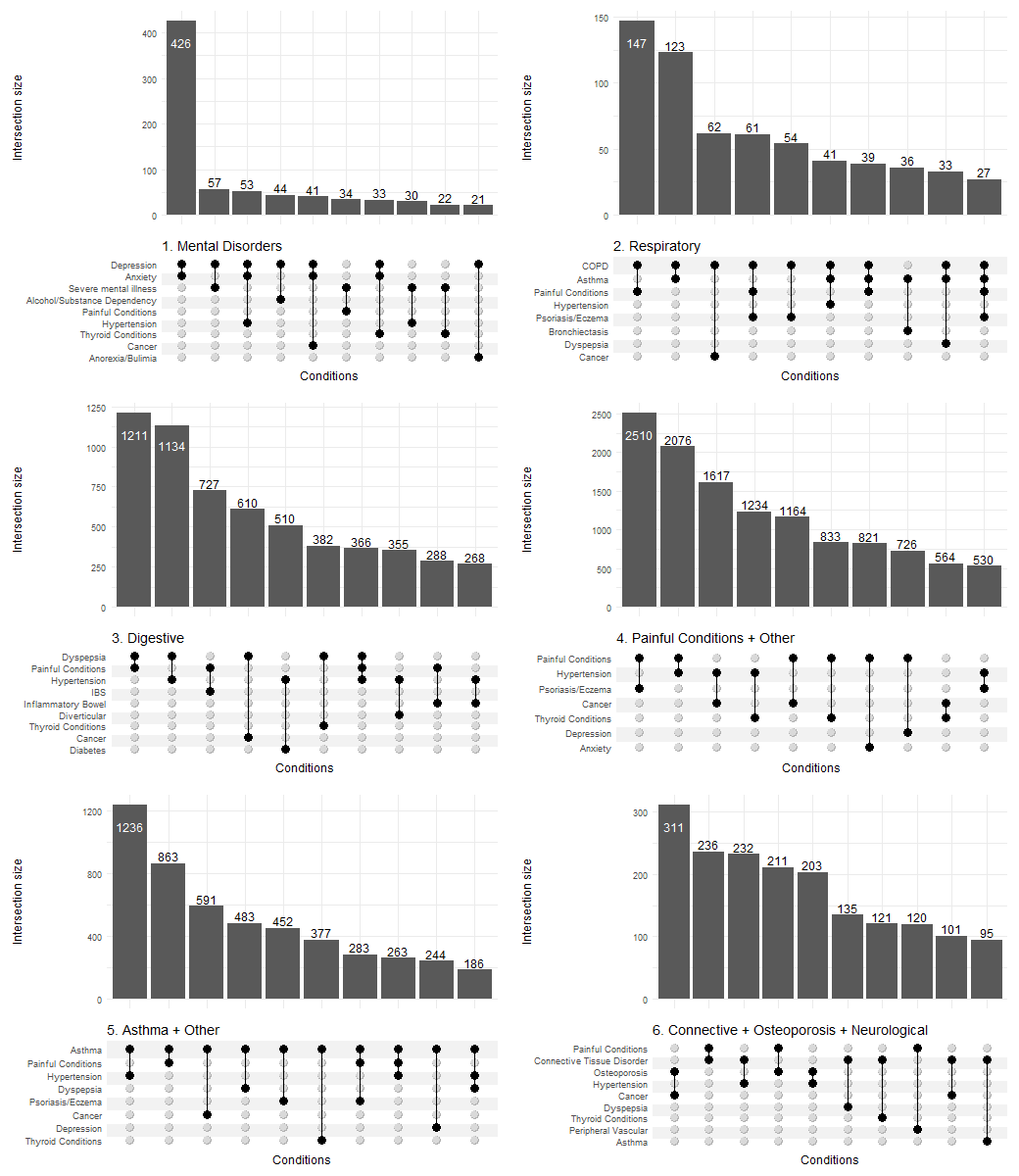


**List of tables & legends**

Supplementary Method Method A: methods used for cluster analyses

Supplementary Method Method B: Men, step 1. Selecting the optimal number of clusters among 73,362 multimorbid men using various clustering metrics

Supplementary Method Method C: Men, step 2. Labeling and summarizing the clusters

Supplementary Method Method D: Table. Summary values at the optimal number of clusters in men

Supplementary Method Method E: Figure. Prevalence, Observed/Expected Ratio, Exclusivity for the seven clusters in men

Supplementary Method Method F: Figure. 10 most frequent combinations of conditions within each cluster in men

Supplementary Method Method G: WOMEN, Step 1. Selecting the optimal number of clusters among 91,261 multimorbid women using various clustering metrics

Supplementary Method Method H: WOMEN, Step 2. Labeling and summarizing the clusters

Supplementary Method Method I: Table. Summary values at the optimal number of clusters in women

Supplementary Method Method J: Figure. Prevalence, Observed/Expected Ratio, Exclusivity for the six clusters in women

Supplementary Method Method K: Figure. 10 most frequent combinations of conditions within each cluster in women
