# Supplementary material for: Comparison of count-based and clustering definitions of multimorbidity and their association with prevalence of multimorbidity, health profiles, and mortality: A cohort study of UK Biobank participants
Source: PLoS Med. 2026 Jun 12;23(6):e1004914. doi: 10.1371/journal.pmed.1004914 (PMC13309015; doi:10.1371/journal.pmed.1004914)
Supplement: Attachment — Submitted filename: PLUSMEDICINE-D-26-00095_R1_reviewer.pdf [file pmed.1004914.s004.pdf]

PLOS Medicine

Comparing count-based and clustering definitions of multimorbidity: implications for prevalence, health profiles, and mortality in the UK Biobank

--Manuscript Draft--

**Manuscript Number:**	PMEDICINE-D-26-00095R1
**Full Title:**	Comparing count-based and clustering definitions of multimorbidity: implications for prevalence, health profiles, and mortality in the UK Biobank
**Short Title:**	Comparing count-based and clustering definitions of multimorbidity
**Article Type:**	Research Article
**Abstract:**	Background<br>Multimorbidity, the presence of several chronic conditions, is linked to higher mortality and healthcare use and thus poses a major challenge for aging populations. While most studies use simple counts of conditions to define multimorbidity, clustering may better reflect how conditions interact and vary across individuals. The impact of these methodological choices on prevalence, as well as on the relationship of multimorbidity with health profiles and mortality, remains unclear.<br>Methods<br>Using UK Biobank data (n=474,397), we compared seven multimorbidity definitions: six count-based with different condition lists (extended, most prevalent or by disease chapter), thresholds (2 or more vs 3 or more) and one based on clustering. We evaluated prevalence and associations with concurrent health outcomes (polypharmacy, self-rated health, frailty, falls, surgery, chronic pain), blood-based measures (C-reactive protein, Cystatin-C, HDL, LDL Cholesterol, IGF-1), and 3- and 10-year mortality risks. Analyses were undertaken separately in men and women using multivariate regression models adjusted for sociodemographic characteristics and body mass index.<br>Results<br>Multimorbidity prevalence ranged from 1.0% (cluster-based) to 35.3% (count-based). Count-based definitions using lists with more conditions yielded higher prevalence. Higher thresholds identified more severe health profiles on all measured health outcomes, blood-based measures but not higher mortality risks. Associations with blood-based measures were more pronounced using clustering, with highest differences from the standard definition distributed across clusters. Odds ratios for 3-year mortality ranged from 1.44 [1.26; 1.64] to 4.60 [3.73; 5.62] for men and 1.35 [1.07; 1.69] to 3.83 [2.78; 5.14] for women. For 10-year mortality they ranged from 1.42 [1.34; 1.50] to 3.86 [3.46; 4.30] in men and 1.29 [1.21; 1.39] to 3.33 [2.93; 3.77] for women, with clustering identifying groups with low prevalence and high mortality risks.<br>Conclusion<br>Methodological choices significantly influence multimorbidity estimates and their clinical implications. While count-based definitions are simple, clustering better identifies high-risk subgroups. Standardization is critical for comparability, but context should guide selection of multimorbidity definition.
**Additional Information:**	
**Question**	**Response**
**Financial Disclosure**<br><br>Enter a financial disclosure statement that describes the sources of funding for the work included in this submission. Review the submission guidelines for detailed requirements. View published research articles from *PLOS Medicine* for specific examples.	Yes

Powered by Editorial Manager® and ProduXion Manager® from Aries Systems Corporation

This statement is required for submission and **will appear in the published article** if the submission is accepted. Please make sure it is accurate.<br><br>**Funded studies**<br>Enter a statement with the following details:<br>• Initials of the authors who received each award<br>• Grant numbers awarded to each author<br>• The full name of each funder<br>• URL of each funder website<br>• Did the sponsors or funders play any role in the study design, data collection and analysis, decision to publish, or preparation of the manuscript?<br><br>Did you receive funding for this work?	
Please add funding details.<br>as follow-up to "**Financial Disclosure**<br><br>Enter a financial disclosure statement that describes the sources of funding for the work included in this submission. Review the submission guidelines for detailed requirements. View published research articles from *PLOS Medicine* for specific examples.<br><br>This statement is required for submission and **will appear in the published article** if the submission is accepted. Please make sure it is accurate.<br><br>**Funded studies**<br>Enter a statement with the following details:<br>• Initials of the authors who received each award<br>• Grant numbers awarded to each author<br>• The full name of each funder<br>• URL of each funder website<br>• Did the sponsors or funders play any role in the study design, data collection and analysis, decision to publish, or preparation of the manuscript?<br><br>Did you receive funding for this work?"	BL - France 2030 ANR-23-PAVH-0006 - Agence National de la Recherche - https://anr.fr/ - No.<br><br>GS - AAP-2023-APAOB-318115 - Institut pour la Recherche en Santé Publique - https://iresp.net/ - No

Please select the country of your main research funder (please select carefully as in some cases this is used in fee calculation).<br>as follow-up to "**Financial Disclosure**<br><br>Enter a financial disclosure statement that describes the sources of funding for the work included in this submission. Review the submission guidelines for detailed requirements. View published research articles from *PLOS Medicine* for specific examples.<br><br>This statement is required for submission and **will appear in the published article** if the submission is accepted. Please make sure it is accurate.<br><br>**Funded studies**<br>Enter a statement with the following details:<br>• Initials of the authors who received each award<br>• Grant numbers awarded to each author<br>• The full name of each funder<br>• URL of each funder website<br>• Did the sponsors or funders play any role in the study design, data collection and analysis, decision to publish, or preparation of the manuscript?<br><br>Did you receive funding for this work?"	FRANCE - FR
**Competing Interests**<br><br>Use the instructions below to enter a competing interest statement for this submission. On behalf of all authors, disclose any competing interests that could be perceived to bias this work—acknowledging all financial support and any other relevant financial or non-financial competing interests.<br><br>This statement **will appear in the published article** if the submission is accepted. Please make sure it is accurate. View published research articles from *PLOS Medicine* for specific	The authors have declared that no competing interests exist.

Powered by Editorial Manager® and ProduXion Manager® from Aries Systems Corporation

examples.<br><br>**NO authors have competing interests**<br><br>Enter: *The authors have declared that no competing interests exist.*<br><br>**Authors with competing interests**<br>Enter competing interest details beginning with this statement:<br><br>*I have read the journal's policy and the authors of this manuscript have the following competing interests: [insert competing interests here]*<br><br>* typeset<br><br>This statement is required for submission and **will appear in the published article** if the submission is accepted. Please make sure it is accurate and that any funding sources listed in your Funding Information later in the submission form are also declared in your Financial Disclosure statement.	
**Ethics Statement**<br><br>You must include an Ethics Statement in the Methods section of your manuscript if your study involved:<br>• Human participants<br>• Human specimens or tissue<br>• Vertebrate animals or cephalopods<br>• Vertebrate embryos or tissues<br>• Field research<br><br>**Not including a statement when required will delay the review process.**<br><br>General guidance is provided below. Please consult the guidelines on human subjects research and animal research for	I confirm that I have read and understood PLOS' requirements for an Ethics Statement.

detailed instructions.<br><br>**Format for specific study types**<br><br>**Human Subject Research (involving human participants and/or tissue)**<br>• Give the name of the institutional review board or ethics committee that approved the study<br>• Include the approval number and/or a statement indicating approval of this research<br>• Indicate the form of consent obtained (written/oral) or the reason that consent was not obtained (e.g. the data were analyzed anonymously)<br><br>**Animal Research (involving vertebrate animals, embryos or tissues)**<br>• Provide the name of the Institutional Animal Care and Use Committee (IACUC) or other relevant ethics board that reviewed the study protocol, and indicate whether they approved this research or granted a formal waiver of ethical approval<br>• Include an approval number if one was obtained<br>• If the study involved *non-human primates*, add *additional details* about animal welfare and steps taken to ameliorate suffering<br><br>**Field Research**<br><br>Include the following details if this study involves the collection of plant, animal, or other materials from a natural setting:<br>• Field permit number<br>• Name of the institution or relevant body that granted permission<br><br>Please check the box to confirm your understanding of this policy, then read and agree to one of the statements.	
Please respond:<br>as follow-up to "**Ethics Statement**	My study requires an ethics statement. I confirm I have included my statement in the Methods section of my uploaded manuscript file.

Powered by Editorial Manager® and ProduXion Manager® from Aries Systems Corporation

You must include an Ethics Statement in the Methods section of your manuscript if your study involved:

- Human participants
- Human specimens or tissue
- Vertebrate animals or cephalopods
- Vertebrate embryos or tissues
- Field research

Not including a statement when required will delay the review process.

General guidance is provided below. Please consult the guidelines on human subjects research and animal research for detailed instructions.

Powered by Editorial Manager® and ProduXion Manager® from Aries Systems Corporation

**Format for specific study types**<br><br>**Human Subject Research (involving human participants and/or tissue)**<br>• Give the name of the institutional review board or ethics committee that approved the study<br>• Include the approval number and/or a statement indicating approval of this research<br>• Indicate the form of consent obtained (written/oral) or the reason that consent was not obtained (e.g. the data were analyzed anonymously)<br><br>**Animal Research (involving vertebrate animals, embryos or tissues)**<br>• Provide the name of the Institutional Animal Care and Use Committee (IACUC) or other relevant ethics board that reviewed the study protocol, and indicate whether they approved this research or granted a formal waiver of ethical approval<br>• Include an approval number if one was obtained<br>• If the study involved *non-human primates*, add *additional details* about animal welfare and steps taken to ameliorate suffering<br><br>**Field Research**<br><br>Include the following details if this study involves the collection of plant, animal, or other materials from a natural setting:<br>• Field permit number<br>• Name of the institution or relevant body that granted permission<br><br>Please check the box to confirm your understanding of this policy, then read and agree to one of the statements. "	
**Data Availability**<br><br>Authors are required to make all data underlying the findings described fully available, without restriction, and from the time of publication. PLOS allows rare exceptions to address legal and ethical concerns. See the PLOS Data Policy and	No - some restrictions will apply

FAQ for detailed information.<br><br>A Data Availability Statement describing where the data can be found is required at submission. Your answers to this question constitute the Data Availability Statement and **will be published in the article**, if accepted.<br><br>**Important:** Stating 'data available on request from the author' is not sufficient. If your data are only available upon request, select 'No' for the first question and explain your exceptional situation in the text box.<br><br>Do the authors confirm that all data underlying the findings described in their manuscript are fully available without restriction?	
**Describe where the data may be found in full sentences. If you are copying our sample text, replace any instances of XXX with the appropriate details.**<br><br>• If the data are **held or will be held in a public repository**, include URLs, accession numbers or DOIs. If this information will only be available after acceptance, indicate this by ticking the box below. For example: *All XXX files are available from the XXX database (accession number(s) XXX, XXX.*).<br>• If the data are all contained **within the manuscript and/or Supporting Information files**, enter the following: *All relevant data are within the manuscript and its Supporting Information files.*<br>• If neither of these applies but you are able to provide **details of access elsewhere**, with or without limitations, please do so. For example:<br><br>*Data cannot be shared publicly because of [XXX]. Data are available from the XXX Institutional Data Access / Ethics Committee (contact via XXX) for*	Data Availability<br>UKB data is available, upon request, by following the application procedure detailed in https://www.ukbiobank.ac.uk/use-our-data/apply-for-access. This work uses data provided by patients and collected by the NHS as part of their care and support.

researchers who meet the criteria for access to confidential data.<br><br>*The data underlying the results presented in the study are available from (include the name of the third party and contact information or URL).*<br>• This text is appropriate if the data are owned by a third party and authors do not have permission to share the data.<br><br>* typeset	
Additional data availability information:	

Powered by Editorial Manager® and ProduXion Manager® from Aries Systems Corporation

Manuscript

Click here to access/download;Manuscript;MMDefinition_UKBiobank_Paper

Comparing count-based and clustering definitions of multimorbidity: implications for prevalence, health profiles, and mortality in the UK Biobank

Gabriella C SILVA, PhD[1]; Aurore FAYOSSE, MSc[1]; Louis JACOB, MD, PhD[1,2,3]; Séverine SABIA, PhD[1,4]; Archana SINGH-MANOUX, PhD[1,4]; Benjamin LANDRÉ, PhD*[1]

1 Université Paris Cité, Inserm U1153, CRESS, Epidemiology of Ageing and Neurodegenerative diseases, Paris, France

2 Université Paris Cité, Assistance Publique – Hôpitaux de Paris (AP-HP), Lariboisière-Fernand Widal Hospital, Department of Physical Medicine and Rehabilitation, Paris 75010, France

3 Research and Development Unit, Parc Sanitari Sant Joan de Déu, CIBERSAM, ISCIII, Dr. Antoni Pujadas, 42, Sant Boi de Llobregat, Barcelona 08030, Spain

4 Faculty of Brain Sciences, University College London, London, UK

*Address for correspondence
Université Paris Cité
Inserm U1153, Epidemiology of Ageing and Neurodegenerative diseases
10 Avenue de Verdun, 75010 Paris, France
Email: benjamin.landre@inserm.fr

Word count:

Abstract: 249/250

Manuscript: 3648/3500

References: 36/30

Tables & figures: 4/5, 2 tables, 2 figures.

Supplementary material: 8 tables, 1 figure, 1 supplementary methods

Keywords

multimorbidity, chronic conditions, clustering, mortality, epidemiology, prevalence, UK Biobank

Abstract

Background

Multimorbidity, the presence of several chronic conditions, is linked to higher mortality and healthcare use and thus poses a major challenge for aging populations. While most studies use simple counts of conditions to define multimorbidity, clustering may better reflect how conditions interact and vary across individuals. The impact of these methodological choices on prevalence, as well as on the relationship of multimorbidity with health profiles and mortality, remains unclear.

Methods

Using UK Biobank data (n=474,397), we compared seven multimorbidity definitions: six count-based with different condition lists (extended, most prevalent or by disease chapter), thresholds (2 or more vs 3 or more) and one based on clustering. We evaluated prevalence and associations with concurrent health outcomes (polypharmacy, self-rated health, frailty, falls, surgery, chronic pain), blood-based measures (C-reactive protein, Cystatin-C, HDL, LDL Cholesterol, IGF-1), and 3- and 10-year mortality risks. Analyses were undertaken separately in men and women using multivariate regression models adjusted for sociodemographic characteristics and body mass index.

Results

Multimorbidity prevalence ranged from 1.0% (cluster-based) to 35.3% (count-based). Count-based definitions using lists with more conditions yielded higher prevalence. Higher thresholds identified more severe health profiles on all measured health outcomes, blood-based measures but not higher mortality risks. Associations with blood-based measures were more pronounced using clustering, with highest differences from the standard definition distributed across clusters. Odds ratios for 3-year mortality ranged from 1.44 [1.26; 1.64] to 4.60 [3.73; 5.62] for men and 1.35 [1.07; 1.69] to 3.83 [2.78; 5.14] for women. For 10-year mortality they ranged from 1.42 [1.34; 1.50] to 3.86 [3.46; 4.30] in men and 1.29 [1.21; 1.39] to 3.33 [2.93; 3.77] for women, with clustering identifying groups with low prevalence and high mortality risks.

Conclusion

Methodological choices significantly influence multimorbidity estimates and their clinical implications. While count-based definitions are simple, clustering better identifies high-risk subgroups. Standardization is critical for comparability, but context should guide selection of multimorbidity definition.

Introduction

Multimorbidity, defined as the presence of multiple chronic conditions in an individual, is increasingly used to describe and understand transitions in health, notably in aging populations. Individuals with multimorbidity consistently experience poorer health outcomes, including higher risks of mortality,[1-3] dementia,[4,5] frailty,[6] and lower quality of life.[7] They also engage more frequently with healthcare systems, leading to higher service utilization and costs.[1,2,8,9]

An umbrella review found that the estimated prevalence of multimorbidity ranges widely, from 3.5% to 100%.[10] Besides differences in study populations, the lack of standardization in defining multimorbidity contributes to substantial variability in prevalence estimates and associations with health outcomes. This inconsistency limits the generalizability of findings, hindering a better understanding of the impact of multimorbidity and the identification of effective interventions or strategies.

The most common approach to measuring multimorbidity involves totalling the number of conditions from a predefined list.[11] Lack of consensus on the list of conditions that should be used has led to extensive variability in the number and type of conditions included in count-based approaches,[12-14] ranging from 2 to 285 according to a recent systematic review.[15] Previous studies relied on self-reported conditions and access to electronic health records has expanded these lists, encoded using different classification schemes with varying levels of granularity.[16,17] This lack of harmonization has contributed to discrepancies in multimorbidity prevalence estimates across studies.[18] Counts of individual conditions or ~~alternatively~~, the affected body systems, generally use a threshold of two or more conditions to define multimorbidity; larger cut-offs such as three or four conditions are considered to identify multimorbid populations with greater needs.[11,16,17]

An alternative to count-based approaches that reflects multimorbidity patterns is clustering. Studies suggest that this method allows better identification of common risk factors, enhanced management of patients' individualized needs,[19,20] and prioritization of patients with the greatest risk.[7,21,22] However, a standardized approach to identify the clusters is lacking.[2]

While previous studies have conceptually compared the strengths and limitations of count-based versus clustering approaches to defining multimorbidity, few have simultaneously evaluated these methods in large scale studies. To our knowledge, no study has directly compared the effect of count-based and clustering multimorbidity measurements on associations between multimorbidity and health profiles. Understanding differences in prevalence and association estimates across these approaches is critical for determining their appropriate applications.

The primary aim of this study is to assess how alternative, commonly used definitions of multimorbidity, including both count-based and clustering methods, affect prevalence estimates and associations with blood-based measures, concurrent health outcomes and subsequent mortality.

Methods

Study population and design

The UK Biobank (UKB) is a population-based cohort study that recruited over 500,000 participants, aged 40-69 years, between 2006 and 2010.[23] At baseline, participants completed an interview, clinical examination, and provided biological samples. Written informed consent was obtained from all participants and ethical approval for the UKB study was granted by the NHS North West Centre for Research Ethics Committee (reference 11/NW/0382). This research used UKB Resource under data sharing application 96856.

Chronic conditions

We identified 38 conditions, spanning 13 ICD-10 (International Classification of Diseases) chapters, using the list defined by Ronaldson et al[24] (**Table S1**). Similar versions of this list have been used in other UKB studies; we made minor modifications to it to account for the availability of data.[3,6] Any chronic conditions occurring prior to initial assessment were ascertained using electronic health records (primary care, hospital admissions, cancer registry, death register) and self-reported data, while excluding conditions reported solely through self-report.

Concurrent health outcomes, blood-based measures and mortality

Health profiles were determined using a set of concurrent health outcomes and blood-based measures.

Health outcomes were assessed at baseline and included polypharmacy (use of five or more medications), poor self-rated health (where participants reported their health as "poor" compared to other responses), Fried's frailty phenotype[25] (three or more impaired criteria as defined by Jiang et al.[26]), falls in the past year, prior surgery (any recorded operations or procedures in hospital records), and chronic pain (pain lasting more than three months in any area – back, facial, general, headache, hip, knee, neck/shoulder, or stomach/abdominal).

Blood-based measures were measured from blood samples collected at baseline. We included C-reactive protein (CRP) (mg/L), Cystatin C (mg/L), HDL cholesterol (mmol/L), IGF-1 (nmol/L), and LDL cholesterol (mmol/L), known to be associated with ageing processes.[27-30] Biomarker data were standardized (mean = 0, standard deviation, SD = 1) separately for men and women prior to analysis.

Mortality over the follow-up was ascertained through November 30, 2022 using the national mortality register. We examined mortality risk at 3- and 10-years.

Covariates

Sociodemographic variables included age, sex (male/female), Townsend deprivation index, and reported ethnicity (White/Non-White). We additionally included body mass index (BMI), calculated using measured height and weight.

Statistical Analyses

We implemented seven definitions of multimorbidity: six based on condition counts and one based on clustering.

For count-based measures, we considered three variations of the condition list (**Table S2**). Count All included all 38 identified conditions, Count MostPrev focused on the 12 most prevalent conditions, as recommended by Fortin et al.[16], and Count Syst counted the number of body organ systems involved, based on the ICD-10 chapter classification.[17] For each list, we used two thresholds to define multimorbidity: having two or more conditions (vs. 0 or 1) and three or more conditions (vs. 0, 1, or 2). For the clustering approach, we replicated the method proposed by Violan et al[31] separately in men and women with at least two conditions according to the Count All measure. Details on the clustering method are provided in **Supplementary Methods: Cluster Analyses**. Briefly, we performed a multiple correspondence analysis followed by k-means clustering on the resulting dimensions.

Subsequent analyses using the seven multimorbidity definitions were composed of (1) comparison of multimorbidity prevalence, (2) associations with health profiles and (3) mortality risk at the 3- and 10-year horizon. All analyses were undertaken separately in men and women.

<u>Comparison of prevalence:</u> For each multimorbidity definition, we calculated the prevalence of multimorbidity, as well as the mean (and SD) condition count and age for multimorbid and non-multimorbid participants.

<u>Cross-sectional associations with concurrent health outcomes and blood-based measures:</u> For the six health outcomes, we used separate multivariate logistic regression models and reported the odds ratio (OR), along with 95% confidence intervals, comparing those identified as multimorbid (or identified as belonging to a particular multimorbidity cluster) to those not identified as multimorbid. Adjustment variables included sociodemographic variables and BMI. For analysis of biomarkers, we used multivariate linear regression models adjusted for the same set of covariates.

<u>Associations with mortality:</u> The association of multimorbidity with mortality at 3 and 10 years of follow-up was examined using multivariate logistic regression, adjusting for sociodemographic factors and BMI.

Analyses were conducted using R (version 4.5.1).[32] The multiple correspondence analysis used for clustering was completed using the mjca function in the ca package (version 0.71.1).[33]

Role of the funding source

The funder had no role in the study design, data collection, analysis, interpretation, or the writing of the report. The corresponding author had full access to the data used in the study and had final responsibility for the decision to submit for publication.

Results

Sample

From the 501,941 participants included in the UKB study at baseline, we excluded 27,544 participants with missing covariate or health outcome data, yielding a sample of 474,397 participants (**Figure S1**). Data on blood-based measures were missing for some participants and so these analyses were based on 405,654 participants with fully available blood-based samples.

The characteristics of the 474,397 participants are shown in **Table S3**; the mean age (SD) was 56.5 years (±8.1), mean Townsend Deprivation Index was -1.4 (±3.1), and mean BMI was 27.4 (±4.8) kg/m^2. Most participants were women (54.5%, n=258,575).

Comparison of prevalence using seven multimorbidity definitions

The prevalence of multimorbidity varied significantly as a function of the multimorbidity definition and sex (**Table 1**). For count approaches, the prevalence of multimorbidity was highest using the full list of 38 conditions and lowest using the reduced list of 12 conditions; the prevalence of multimorbidity in men (women) was 34.0% (35.3%) for *Count All: 2+*, 26.4% (29.1%) for *Count MostPrev: 2+*, and 31.8% (33.9%) for *Count Syst: 2+*. The prevalence for approaches using a threshold of 3+ conditions to define multimorbidity was lower than that using 2+. The mean count of conditions and mean age of participants were greater using the 3+ definition. For instance, while the mean age and condition count for multimorbid men in *Count All: 2+* were 59.5 years and 3.1 conditions, men who were multimorbid using the *Count All: 3+* definition were, on average, 60.3 years and had 4.0 conditions.

The clustering approach (**Supplementary Methods: Cluster Analyses**), which stratified participants identified as multimorbid using the *Count All: 2+* definition (n=73,362 men and 91,261 women) based on the combination of their conditions, identified seven multimorbidity clusters in men and six in women. Based on the prevalence of conditions, observed/expected ratio, and exclusivity of disease in each identified cluster, in men the clusters and their respective prevalence were: liver + alcohol + dementia (1.2%), circulatory (1.4%), mental disorders (3.7%), digestive + osteoporosis (4.1%), respiratory (4.9%), hypertension + other (8.2%), and painful conditions + cardiovascular and metabolic disorders (10.5%). In women, the clusters were: respiratory (1.0%), mental disorders (1.1%), connective + osteoporosis + neurological (4.9%), asthma + other (5.3%), digestive (9.2%), and painful conditions + other (13.7%). The most frequent combinations of disease for each cluster are shown in **Supplementary Methods: Cluster Analyses**. There was greater variation in mean ages and condition counts using the clustering approach, compared to multimorbidity definitions based on counts. For those considered multimorbid, the mean number of conditions varied between 2.5 (hypertension + other) and 4.9 (circulatory) in men and between 2.7 (painful conditions + other) and 4.2 (respiratory) in women. Men and women in the mental disorder clusters were younger compared to other multimorbidity clusters (mean age in men = 56.9, women = 54.6 years).

The most common concurrent health outcome was surgery (62.0%) and least common was frailty (4.0%) (**Table S3**). 4.7% of the study population had died at the 10-year time horizon (**Table S3**). Further details of concurrent health outcomes and mortality as a function of multimorbidity measures are shown in **Table S4** for men and **Table S5** for women.

Cross-sectional association of multimorbidity measures with health outcomes

All multimorbidity definitions were associated with health outcomes measured concurrently in men and women, as displayed in **Table 2**. The count-based approaches generally had the largest ORs for definitions using the full list of 38 conditions, while the smallest ORs were associated with definitions based on the 12 most prevalent conditions. Definitions using a threshold of three conditions to define multimorbidity yielded stronger associations compared to those using a threshold of two.

In men and women, the digestive cluster had comparatively large ORs of surgery and chronic pain. Among the other concurrent health outcomes considered, the largest OR estimates in men were generally linked to the circulatory and liver + alcohol + dementia clusters, corresponding to the smallest-prevalence clusters. In women, the respiratory and connective + osteoporosis + neurological clusters consistently had the largest ORs for associations with health outcomes.

Cross-sectional association of multimorbidity measures with blood-based measures

The mean values of standardized CRP, Cystatin C, HDL cholesterol, IGF-1, and LDL cholesterol are shown in **Table S6** and estimated coefficients for the association between multimorbidity measures and these measures in **Figure 1**, along with **Table S7**, in the sub-cohort of 405,654 participants with complete blood-based measures. In both men and women, regardless of the multimorbidity definition considered, multimorbidity was associated with higher levels of CRP and Cystatin C, and lower levels of HDL cholesterol, IGF-1, and LDL cholesterol, relative to those not identified as multimorbid. The magnitude of these associations was consistently larger when using a threshold of three conditions compared to two.

The coefficients for multimorbidity estimated for some of the clusters showed significant departures from those estimated for the other definitions of multimorbidity. For example, in men, while the estimated impact of multimorbidity on Cystatin C levels was 0.22 (0.21, 0.23) SD using *Count All: 2+*, the corresponding estimate for the circulatory cluster was 0.61 (0.58, 0.65) SD. In women, the estimated coefficient for multimorbidity on CRP levels was 0.11 (0.10, 0.12) SD using *Count All: 2+*, though those in the respiratory cluster had 0.46 (0.42, 0.50) SD higher levels of this protein compared to non-multimorbid women.

Association of Multimorbidity with Mortality

The risk of mortality at 3- and 10-years after initial assessment is displayed in **Figure 2** (corresponding results in **Table S8**). All ORs were greater than 1 and statistically significant, indicating a higher risk of

mortality in those with multimorbidity compared to non-multimorbid participants. The choice of threshold (two or three) and condition list both had minimal impact on the estimated association between multimorbidity and mortality. The magnitude of the ORs was highest using the full list of conditions and lowest using the MostPrev list, with corresponding prevalence's following the same pattern. In men, the OR (95% CI) for 10-year mortality was 1.70 (1.64, 1.76) using *Count All: 2+*, 1.64 (1.58, 1.70) using *Count Syst: 2+*, and 1.54 (1.49, 1.60) using *Count MostPrev: 2+*. A similar pattern was observed in women, with ORs of 1.67 (1.60, 1.75) for *Count All: 3+*, 1.65 (1.57, 1.73) for *Count Syst: 3+*, and 1.53 (1.45, 1.61) for *Count MostPrev: 3+*.

The cluster-based approach showed greater variability in estimated ORs compared to count-based definitions. Clusters with the lowest prevalence were associated with the highest odds of mortality. In men, those in the liver + alcohol + dementia and circulatory clusters had ORs (95% CI) of 3.86 (3.46, 4.30) and 3.03 (2.74, 3.34), respectively, for 10-year mortality, compared to non-multimorbid men. These ORs were significantly higher than those for men in the painful conditions + cardiovascular and metabolic disorders cluster, that had an OR (95% CI) of 1.42 (1.34, 1.50) and was the most prevalent cluster. In women, the mental disorders and respiratory clusters, both of which had the lowest prevalence, were associated with the highest ORs for mortality.

Discussion

This study examined the impact of differences in definitions of multimorbidity on our understanding of its prevalence and its association with health profiles and risk of mortality in a large-scale longitudinal cohort study. We identified significant variations in multimorbidity prevalence, ranging from 1.0% for the smallest multimorbidity cluster to 35.3% using the full list of 38 chronic conditions and the threshold of two or more conditions. Lists with fewer conditions resulted in lower prevalence estimates. We also found that while the choice of cut-off used for defining who was multimorbid had a large effect on the estimated prevalence's of multimorbidity, it had a smaller impact on associations with concurrent health outcomes, blood-based measures and mortality risk. An important aspect of our study is comparison of these count-based definitions with the clustering approach, which is thought to better identify patients with high risk and address their needs.[19,20] Clustering yielded significantly more variation in prevalence and association estimates, as compared to count-based approaches. Our results show that some clusters that were not very prevalent had extremely strong associations with health status; this was the case for the liver + alcohol + dementia and circulatory clusters in men and the respiratory and mental disorders clusters in women.

Previous studies have similarly identified that as the number of conditions listed in multimorbidity definitions increases, the prevalence of multimorbidity rises.[11,17] We found an approximately 5.5-8.0% difference in prevalence using the full list of 38 conditions compared to using the reduced list of 12 proposed by Fortin.[16] Harrison and colleagues[17] found a slightly larger difference using the 2+ or 3+ condition threshold; the prevalence of multimorbidity was 37.0% and 23.4%, respectively, using Fortin's reduced list[16] and 47.4% and 33.8%, respectively, using their list of chronic conditions. A study using Canadian data[11] similarly found higher prevalence using 60 conditions compared to a reduced list of 20. We identified less than a 3.5% difference in prevalence estimated using the granular, full list (38 chronic conditions) and that estimated using ICD chapter counts. The difference was larger when a threshold of two or more conditions was used to define multimorbidity, comparable to what Harrison and colleagues[17] identified when comparing treating every chronic condition as a separate disease entity versus counting the number of chapters affected by at least one condition.

Despite emerging consensus on a cut-off of three or more conditions being of greater clinical use as it captures a more vulnerable population, two or more conditions remain widely used in research studies.[8,11,17] The decline in prevalence of multimorbidity when increasing the threshold from two to three was most pronounced in definitions using counts of affected ICD-10 chapters. Using a list of 40 conditions and UK Biobank study, Hanlon and colleagues[34] found increasing the cut-off for number of conditions from two to three reduced the prevalence of multimorbidity by 13.8%; in our study on 38 conditions, the corresponding numbers were 16.0% in men and 16.2% in women. Results from another study confirm this pattern.[17]

The clustering approach identified meaningful combinations of conditions among those identified as multimorbid according to the *Count All: 2+* definition. It led to large variations in the prevalence of

these combinations and enabled us to identify specific subgroups of multimorbid participants that had higher risks of mortality and suboptimal health profiles. In men, the liver + alcohol + dementia and circulatory clusters were not very prevalent (1.2% and 1.4%, respectively) but the health profiles, determined using the six outcomes and five blood-based measures, were poorer and risk of mortality higher than in other clusters. In women, the mental disorders and respiratory clusters, with prevalence of 1.1% and 1.0% respectively, had higher mortality risk than the other clusters. The respiratory cluster in women also had a poorer health profile, including higher CRP levels. A recent study using UK Biobank data, conducted using a different condition list and clustering approach, identified similar clusters to ours and found the mental health and respiratory clusters for women and the cardiometabolic condition cluster for men to be associated with higher mortality risk.[22] Steell and colleagues noted the presence of pulmonary and cardiometabolic clusters in UK Biobank study and reported lower health-related quality of life in these clusters, compared to the not multimorbid group, to varying extents.[7] The insights from these previous studies, along with the results provided from this study, highlight the existence of non-random multimorbidity clusters and the higher risk of poorer health outcomes in certain clusters.

Our results have important implications for how multimorbidity ought to be defined and measured in future studies. This choice is likely to depend on the context and whether the goal is epidemiological description, identification of risk factor, management of patients at greater risk of adverse events, or evaluation of interventions. Key considerations include the need for comparability across studies, the ability to identify profiles at higher risk of adverse outcomes, and, particularly for interventions, the size of the groups that ought to be targeted for individual or large-scale interventions. Use of a standardized list, such as the one proposed by Ho et al.[14], is important for replication but faces challenges related to access to data and harmonization of relevant coding schemes for chronic conditions. Our findings suggest that the choice of conditions in a list, used by both the count-based and clustering approaches, has an impact on certain health correlates of multimorbidity. A Delphi consensus recommended defining multimorbidity using a simple condition count with a threshold of two or more conditions and no *a priori* limitation on the list of conditions, but it also suggested that severe multimorbidity ought to be measured using a threshold of three and above.[14] Our findings suggest that use of a higher threshold on the number of chronic conditions does not necessarily translate into significantly greater mortality risks, and that cluster-based approaches may better capture the complexity of health profiles and identify vulnerable groups. As compared to the count-based approach, clustering identifies smaller multimorbidity groups that are each less heterogenous in terms of conditions. However, labelling and defining the clusters remains challenging.

A primary strength of our study is the use of count and clustering methods to define multimorbidity and its impact on health in the same dataset. To allow for replication and facilitate comparisons with existing research, we used a previously developed clustering procedure[31] and a list of conditions employed in previous UK Biobank multimorbidity studies.[24] While use of the UK Biobank data for these analyses

provided access to a large sample size and a rich, comprehensive set of health data, previous studies have shown that the cohort study is not representative of the UK population.[35] Findings may not be generalizable to more vulnerable populations, such as institutionalized adults. In addition, some of the variables used in this study are based on self-reported data, which may be subject to bias.[36] A further limitation is that we did not consider the impact of some of the other previously identified differences in multimorbidity measurement, such as varying the lookback period or considering the use of self-reported versus electronic health records to define chronic conditions. Modifying these factors was outside the scope of our study, as we wished to focus on the most commonly reported differences in multimorbidity measurement across previous studies. Finally, the health outcomes and blood-based measures could only be examined cross-sectionally and no conclusions on the causal direction of associations can be drawn from these analyses.

Given the expected increase in multimorbidity prevalence linked to global aging and its impact on health, our results emphasize the need for consensus on definition and measurement of multimorbidity. The choice of a definition significantly impacts key epidemiological indicators and depends on both the research or clinical context, as well as the availability of data. We encourage researchers to carefully consider and justify their choice of definition when designing studies or interventions, as without such transparency, the potential for translational research on multimorbidity may remain limited.

Declaration of interest

The authors have declared that no competing interests exist.

Acknowledgment

This project is funded by a grant from "France 2030 ANR-23-PAVH-0006" and by IReSP/CNSA as part of the 2023 call for projects for the research support program "Older people, people with disabilities at all stages of life, relatives, and professionals," supported by the National Solidarity Fund for Autonomy (CNSA) "AAP-2023-APAOB-318115".

Role of the funding source

The funder had no role in the study design, data collection, data analysis, data interpretation, or the writing of the report. The corresponding author had full access to all the data in the study and had final responsibility for the decision to submit for publication.

Data Availability

UKB data is available, upon request, by following the application procedure detailed in https://www.ukbiobank.ac.uk/use-our-data/apply-for-access. This work uses data provided by patients and collected by the NHS as part of their care and support.

References

1. Gulea C, Zakeri R, Quint JK. Model-based comorbidity clusters in patients with heart failure: association with clinical outcomes and healthcare utilization. *BMC Med* 2021; **19**(1): 9.

2. Xi J, Miao M, Li PWC, Yu DSF. Prognostic effects of multimorbidity clusters on health outcomes in adults: A systematic review and meta-analysis. *Ageing Res Rev* 2025; **112**: 102897.

3. Jani BD, Hanlon P, Nicholl BI, et al. Relationship between multimorbidity, demographic factors and mortality: findings from the UK Biobank cohort. *BMC Med* 2019; **17**(1): 74.

4. Patel R, Gillis G, Mackay CE, et al. The lifetime accumulation of multimorbidity and its influence on dementia risk: a UK Biobank study. *Brain Commun* 2025; **7**(4): fcaf222.

5. Shang X, Zhang X, Huang Y, et al. Association of a wide range of individual chronic diseases and their multimorbidity with brain volumes in the UK Biobank: A cross-sectional study. *EClinicalMedicine* 2022; **47**: 101413.

6. Hanlon P, Nicholl BI, Jani BD, Lee D, McQueenie R, Mair FS. Frailty and pre-frailty in middle-aged and older adults and its association with multimorbidity and mortality: a prospective analysis of 493 737 UK Biobank participants. *Lancet Public Health* 2018; **3**(7): e323-e32.

7. Steell L, Krauth SJ, Ahmed S, et al. Multimorbidity clusters and their associations with health-related quality of life in two UK cohorts. *BMC Med* 2025; **23**(1): 1.

8. Skou ST, Mair FS, Fortin M, et al. Multimorbidity. *Nat Rev Dis Primers* 2022; **8**(1): 48.

9. Ubalde-Lopez M, Delclos GL, Benavides FG, Calvo-Bonacho E, Gimeno D. The effect of multimorbidity on sickness absence by specific diagnoses. *Occup Med (Lond)* 2017; **67**(2): 93-100.

10. Xu X, Mishra GD, Jones M. Evidence on multimorbidity from definition to intervention: An overview of systematic reviews. *Ageing Res Rev* 2017; **37**: 53-68.

11. Simard M, Rahme E, Dube M, Boiteau V, Talbot D, Sirois C. Multimorbidity prevalence and health outcome prediction: assessing the impact of lookback periods, disease count, and definition criteria in health administrative data at the population-based level. *BMC Med Res Methodol* 2024; **24**(1): 113.

12. Diederichs C, Berger K, Bartels DB. The measurement of multiple chronic diseases--a systematic review on existing multimorbidity indices. *J Gerontol A Biol Sci Med Sci* 2011; **66**(3): 301-11.

13. Johnston MC, Crilly M, Black C, Prescott GJ, Mercer SW. Defining and measuring multimorbidity: a systematic review of systematic reviews. *Eur J Public Health* 2019; **29**(1): 182-9.

14. Ho ISS, Azcoaga-Lorenzo A, Akbari A, et al. Measuring multimorbidity in research: Delphi consensus study. *BMJ Med* 2022; **1**(1): e000247.

15. Ho IS, Azcoaga-Lorenzo A, Akbari A, et al. Examining variation in the measurement of multimorbidity in research: a systematic review of 566 studies. *Lancet Public Health* 2021; **6**(8): e587-e97.

16. Fortin M, Stewart M, Poitras ME, Almirall J, Maddocks H. A systematic review of prevalence studies on multimorbidity: toward a more uniform methodology. *Ann Fam Med* 2012; **10**(2): 142-51.

17. Harrison C, Britt H, Miller G, Henderson J. Examining different measures of multimorbidity, using a large prospective cross-sectional study in Australian general practice. *BMJ Open* 2014; **4**(7): e004694.

18. Fortin M, Haggerty J, Sanche S, Almirall J. Self-reported versus health administrative data: implications for assessing chronic illness burden in populations. A cross-sectional study. *CMAJ Open* 2017; **5**(3): E729-E33.

19. Prados-Torres A, Poblador-Plou B, Calderon-Larranaga A, et al. Multimorbidity patterns in primary care: interactions among chronic diseases using factor analysis. *PLoS One* 2012; **7**(2): e32190.

20. Whitty CJM, Watt FM. Map clusters of diseases to tackle multimorbidity. *Nature* 2020; **579**(7800): 494-6.

21. Prasad B, Bjourson AJ, Shukla P. Data-driven patient stratification of UK Biobank cohort suggests five endotypes of multimorbidity. *Brief Bioinform* 2022; **23**(6).

22. Littlejohns TJ, Liu W, Calvin CM, et al. Multimorbidity, disease clusters and risk of all-cause and cause-specific mortality: a population-based prospective cohort study. *Sci Rep* 2025; **15**(1): 41393.

23. Littlejohns TJ, Holliday J, Gibson LM, et al. The UK Biobank imaging enhancement of 100,000 participants: rationale, data collection, management and future directions. *Nat Commun* 2020; **11**(1): 2624.

24. Ronaldson A, Arias de la Torre J, Ashworth M, et al. Associations between air pollution and multimorbidity in the UK Biobank: A cross-sectional study. *Front Public Health* 2022; **10**: 1035415.

25. Fried LP, Tangen CM, Walston J, et al. Frailty in older adults: evidence for a phenotype. *J Gerontol A Biol Sci Med Sci* 2001; **56**(3): M146-56.

26. Jiang R, Noble S, Sui J, et al. Associations of physical frailty with health outcomes and brain structure in 483 033 middle-aged and older adults: a population-based study from the UK Biobank. *Lancet Digit Health* 2023; **5**(6): e350-e9.

27. Lopez-Otin C, Blasco MA, Partridge L, Serrano M, Kroemer G. The hallmarks of aging. *Cell* 2013; **153**(6): 1194-217.

28. Sarnak MJ, Katz R, Fried LF, et al. Cystatin C and aging success. *Arch Intern Med* 2008; **168**(2): 147-53.

29. Baechle JJ, Chen N, Makhijani P, Winer S, Furman D, Winer DA. Chronic inflammation and the hallmarks of aging. *Mol Metab* 2023; **74**: 101755.

30. Sharma R, Diwan B. Lipids and the hallmarks of ageing: From pathology to interventions. *Mech Ageing Dev* 2023; **215**: 111858.

31. Violan C, Roso-Llorach A, Foguet-Boreu Q, et al. Multimorbidity patterns with K-means nonhierarchical cluster analysis. *BMC Fam Pract* 2018; **19**(1): 108.

32. R: A language and environment for statistical computing. R Development Core Team: R Foundation for Statistical Computing; 2010.

33. Nenadic O, Greenacre M. Correspondence Analysis in R, with Two- and Three-dimensional Graphics: The ca Package. *Journal of Statistical Software* 2007; **20**(3): 1 - 13.

34. Hanlon P, Jani BD, Nicholl B, Lewsey J, McAllister DA, Mair FS. Associations between multimorbidity and adverse health outcomes in UK Biobank and the SAIL Databank: A comparison of longitudinal cohort studies. *PLoS Med* 2022; **19**(3): e1003931.

35. Chudasama YV, Khunti K, Gillies CL, et al. Healthy lifestyle and life expectancy in people with multimorbidity in the UK Biobank: A longitudinal cohort study. *PLoS Med* 2020; **17**(9): e1003332.
36. Hanlon P, Jani BD, Butterly E, et al. An analysis of frailty and multimorbidity in 20,566 UK Biobank participants with type 2 diabetes. *Commun Med (Lond)* 2021; **1**: 28.

Table 1. Multimorbidity prevalence, count of chronic conditions, and age at baseline in men and women.

Multimorbidity measure[1]			Prevalence % (n)	Condition Count[2] Mean (SD)	Age at Baseline Mean (SD)
**MEN (N=215,822)**					
**Count All**	**2+**	No Multimorbidity	66.0 (142,460)	0.4 (0.5)	55.3 (8.2)
		Multimorbidity	34.0 (73,362)	3.1 (1.4)	59.5 (7.4)
	**3+**	No Multimorbidity	82.0 (176,898)	0.7 (0.8)	55.9 (8.2)
		Multimorbidity	18.0 (38,924)	4.0 (1.3)	60.3 (7.1)
**Count MostPrev**	**2+**	No Multimorbidity	73.6 (158,740)	0.4 (0.5)	55.7 (8.2)
		Multimorbidity	26.4 (57,082)	2.6 (0.9)	59.4 (7.4)
	**3+**	No Multimorbidity	89.0 (192,155)	0.7 (0.8)	56.3 (8.2)
		Multimorbidity	11.0 (23,667)	3.5 (0.8)	60.0 (7.2)
**Count Syst**	**2+**	No Multimorbidity	68.2 (147,241)	0.4 (0.5)	55.4 (8.2)
		Multimorbidity	31.8 (68,581)	2.7 (1.0)	59.5 (7.4)
	**3+**	No Multimorbidity	85.3 (184,139)	0.7 (0.8)	56.1 (8.2)
		Multimorbidity	14.7 (31,683)	3.6 (0.9)	60.3 (7.1)
**Clustering**		No Multimorbidity	66.0 (142,460)	0.4 (0.5)	55.3 (8.2)
		Mental Disorders	3.7 (7,994)	3.1 (1.3)	56.9 (7.9)
		Respiratory	4.9 (10,490)	3.2 (1.4)	58.6 (7.8)
		Digestive + Osteoporosis	4.1 (8,929)	3.5 (1.6)	59.7 (7.5)
		Painful Conditions + Cardiovascular and Metabolic disorders	10.5 (22,605)	2.8 (1.1)	59.1 (7.4)
		Hypertension + Other	8.2 (17,769)	2.5 (0.8)	61.6 (6.3)
		Liver + Alcohol + Dementia	1.2 (2,518)	4.2 (2.0)	57.6 (7.6)
		Circulatory	1.4 (3,057)	4.9 (2.0)	60.9 (6.7)
**WOMEN (N=258,575)**					
**Count All**	**2+**	No Multimorbidity	64.7 (167,314)	0.4 (0.5)	55.3 (8.0)
		Multimorbidity	35.3 (91,261)	3.1 (1.4)	58.2 (7.6)
	**3+**	No Multimorbidity	80.9 (209,058)	0.7 (0.8)	55.8 (8.0)
		Multimorbidity	19.1 (49,517)	4.0 (1.4)	58.7 (7.5)
**Count MostPrev**	**2+**	No Multimorbidity	70.9 (183,456)	0.4 (0.5)	55.5 (8.0)
		Multimorbidity	29.1 (75,119)	2.8 (1.0)	58.3 (7.6)
	**3+**	No Multimorbidity	86.6 (224,009)	0.7 (0.8)	56.0 (8.0)
		Multimorbidity	13.4 (34,566)	3.7 (1.0)	58.7 (7.5)
**Count Syst**	**2+**	No Multimorbidity	66.1 (170,924)	0.4 (0.5)	55.4 (8.0)
		Multimorbidity	33.9 (87,651)	2.8 (1.0)	58.2 (7.6)
	**3+**	No Multimorbidity	83.4 (215,715)	0.8 (0.8)	55.9 (8.0)
		Multimorbidity	16.6 (42,860)	3.7 (0.9)	58.7 (7.5)
**Clustering**		No Multimorbidity	64.7 (167,314)	0.4 (0.5)	55.3 (8.0)
		Mental Disorders	1.1 (2,768)	3.7 (1.9)	54.6 (7.9)
		Respiratory	1.0 (2,680)	4.2 (2.0)	60.0 (6.9)
		Digestive	9.2 (23,868)	3.2 (1.5)	58.7 (7.4)
		Painful Conditions + Other	13.7 (35,516)	2.7 (1.0)	58.1 (7.6)
		Asthma + Other	5.3 (13,721)	3.1 (1.4)	57.0 (8.0)
		Connective + Osteoporosis + Neurological	4.9 (12,708)	3.5 (1.7)	59.4 (7.3)

Abbreviations: Count MostPrev = 12 most prevalent conditions in the list; Count Syst = count of body-systems involved. 2+ = multimorbidity defined using 2 or more conditions; 3+ = multimorbidity defined using 3 or more conditions

[1]Multimorbidity definitions are based on counting conditions or clustering. Count All, Count MostPrev and Count Syst, count the number of all conditions, 12 most prevalent conditions, or number of body systems involved by conditions, respectively. For count-based approaches, 2+ or 3+ thresholds were used to categorize participants as multimorbid or not. Cluster groups were identified among those multimorbid based on the Count All: 2+ Measure.

[2]The condition count is reported using the respective list of conditions for each method.

Table 2. Cross-sectional associations of multimorbidity measures with health outcomes in men and women.

Multimorbidity measures[1]		Prevalence %	Health outcomes (Odds Ratio (95% Confidence Interval)[2]					
			Polypharmacy	Poor self-rated health	Frailty	Falls	Surgery	Chronic Pain
**MEN (N=215,822), mean (SD) age: 56.7 (8.2) years**								
**Count All**	**2+**	34.0	6.02 (5.86, 6.18)	5.43 (5.18, 5.69)	3.74 (3.54, 3.95)	1.45 (1.41, 1.49)	4.21 (4.12, 4.30)	1.68 (1.65, 1.71)
	**3+**	18.0	6.24 (6.07, 6.40)	5.72 (5.47, 5.97)	4.17 (3.96, 4.39)	1.57 (1.53, 1.62)	5.21 (5.04, 5.37)	1.82 (1.78, 1.86)
**Count MostPrev**	**2+**	26.4	4.99 (4.86, 5.11)	4.27 (4.09, 4.46)	3.06 (2.91, 3.23)	1.41 (1.38, 1.45)	3.61 (3.53, 3.70)	1.72 (1.69, 1.76)
	**3+**	11.0	5.57 (5.40, 5.74)	4.64 (4.44, 4.86)	3.51 (3.33, 3.71)	1.56 (1.51, 1.62)	4.66 (4.49, 4.85)	1.96 (1.91, 2.02)
**Count Syst**	**2+**	31.8	5.01 (4.88, 5.14)	4.92 (4.70, 5.15)	3.53 (3.35, 3.73)	1.46 (1.42, 1.50)	3.81 (3.73, 3.90)	1.70 (1.67, 1.73)
	**3+**	14.7	5.06 (4.92, 5.20)	4.98 (4.76, 5.20)	3.82 (3.62, 4.02)	1.61 (1.56, 1.65)	4.43 (4.28, 4.58)	1.85 (1.80, 1.90)
**Clustering**	Mental Disorders	3.7	5.35 (5.07, 5.65)	6.41 (5.93, 6.92)	4.48 (4.07, 4.92)	1.81 (1.71, 1.91)	3.24 (3.07, 3.41)	1.81 (1.73, 1.90)
	Respiratory	4.9	7.98 (7.62, 8.36)	6.96 (6.48, 7.46)	4.54 (4.17, 4.95)	1.50 (1.42, 1.57)	3.38 (3.22, 3.54)	1.76 (1.69, 1.83)
	Digestive + Osteoporosis	4.1	4.97 (4.72, 5.23)	6.41 (5.92, 6.93)	4.04 (3.66, 4.45)	1.40 (1.32, 1.47)	10.13 (9.42, 10.90)	2.06 (1.97, 2.15)
	Painful Conditions + Cardiovascular and Metabolic disorders	10.5	5.49 (5.30, 5.69)	3.89 (3.65, 4.14)	3.25 (3.02, 3.49)	1.37 (1.32, 1.42)	2.81 (2.72, 2.90)	1.63 (1.59, 1.68)
	Hypertension + Other	8.2	5.29 (5.09, 5.50)	3.76 (3.50, 4.05)	2.34 (2.14, 2.55)	1.27 (1.22, 1.33)	6.04 (5.78, 6.31)	1.42 (1.37, 1.47)
	Liver + Alcohol + Dementia	1.2	7.74 (7.09, 8.43)	10.10 (9.06, 11.25)	6.48 (5.68, 7.37)	1.98 (1.80, 2.16)	7.22 (6.41, 8.16)	1.91 (1.77, 2.07)
	Circulatory	1.4	16.58 (15.31, 17.97)	11.83 (10.68, 13.08)	7.01 (6.20, 7.91)	1.72 (1.58, 1.87)	16.11 (13.86, 18.86)	1.75 (1.63, 1.89)
**WOMEN (N=258,575), mean (SD) age: 56.3 (8.0) years**								
**Count All**	**2+**	35.3	4.33 (4.23, 4.43)	5.41 (5.15, 5.68)	3.20 (3.07, 3.33)	1.36 (1.33, 1.38)	3.44 (3.38, 3.51)	1.86 (1.82, 1.89)
	**3+**	19.1	4.69 (4.59, 4.80)	5.65 (5.41, 5.91)	3.41 (3.28, 3.55)	1.45 (1.42, 1.48)	4.10 (3.99, 4.22)	2.06 (2.02, 2.10)
**Count MostPrev**	**2+**	29.1	3.82 (3.74, 3.90)	4.28 (4.09, 4.48)	2.78 (2.67, 2.89)	1.34 (1.31, 1.37)	3.16 (3.09, 3.23)	1.83 (1.80, 1.86)
	**3+**	13.4	4.30 (4.19, 4.41)	4.69 (4.48, 4.90)	2.99 (2.87, 3.12)	1.44 (1.41, 1.48)	3.93 (3.81, 4.06)	2.09 (2.04, 2.14)
**Count Syst**	**2+**	33.9	4.16 (4.07, 4.25)	5.11 (4.87, 5.36)	3.11 (2.99, 3.24)	1.36 (1.33, 1.38)	3.32 (3.25, 3.39)	1.85 (1.82, 1.88)
	**3+**	16.6	4.40 (4.30, 4.51)	5.19 (4.97, 5.43)	3.25 (3.12, 3.38)	1.44 (1.41, 1.47)	3.84 (3.73, 3.95)	2.05 (2.01, 2.10)
**Clustering**	Mental Disorders	1.1	4.90 (4.51, 5.33)	8.70 (7.71, 9.79)	4.64 (4.11, 5.23)	1.75 (1.61, 1.90)	3.44 (3.12, 3.81)	1.73 (1.60, 1.87)
	Respiratory	1.0	8.16 (7.53, 8.85)	16.25 (14.53, 18.15)	6.78 (6.06, 7.58)	1.47 (1.36, 1.60)	4.44 (3.99, 4.95)	2.16 (2.00, 2.34)
	Digestive	9.2	4.10 (3.97, 4.24)	5.12 (4.79, 5.47)	2.93 (2.77, 3.11)	1.29 (1.25, 1.33)	6.76 (6.47, 7.06)	2.16 (2.10, 2.23)
	Painful Conditions + Other	13.7	3.03 (2.94, 3.12)	3.31 (3.10, 3.53)	2.27 (2.15, 2.40)	1.22 (1.19, 1.25)	2.31 (2.25, 2.37)	1.56 (1.53, 1.60)
	Asthma + Other	5.3	6.19 (5.95, 6.43)	5.06 (4.68, 5.45)	3.04 (2.84, 3.25)	1.44 (1.38, 1.49)	3.20 (3.07, 3.35)	1.95 (1.88, 2.02)
	Connective + Osteoporosis + Neurological	4.9	6.71 (6.45, 6.99)	10.91 (10.17, 11.70)	6.09 (5.72, 6.49)	1.71 (1.65, 1.78)	4.73 (4.50, 4.98)	2.12 (2.04, 2.20)

Abbreviations: Count MostPrev = 12 most prevalent conditions in the list; Count Syst = count of body-systems involved. 2+ = multimorbidity defined using 2 or more conditions; 3+ = multimorbidity defined using 3 or more conditions

[1]Multimorbidity definitions are based on counting conditions or clustering. Count All, Count MostPrev and Count Syst count the number of all conditions, 12 most prevalent conditions or number of body-systems affected, respectively. Cluster groups were identified among those multimorbid based on the Count All: 2+ Measure.

[2]Logistic regression adjusted for age, Townsend deprivation index, ethnicity, and body mass index. Odds Ratios represent the odds of a health outcome among those with the corresponding multimorbid status, as compared to the reference group of those identified as “not multimorbid”.

Figure 1. Cross-sectional association (regression coefficient) between multimorbidity measures and blood-based measures in men (N = 186,873) and women (N = 218,781).

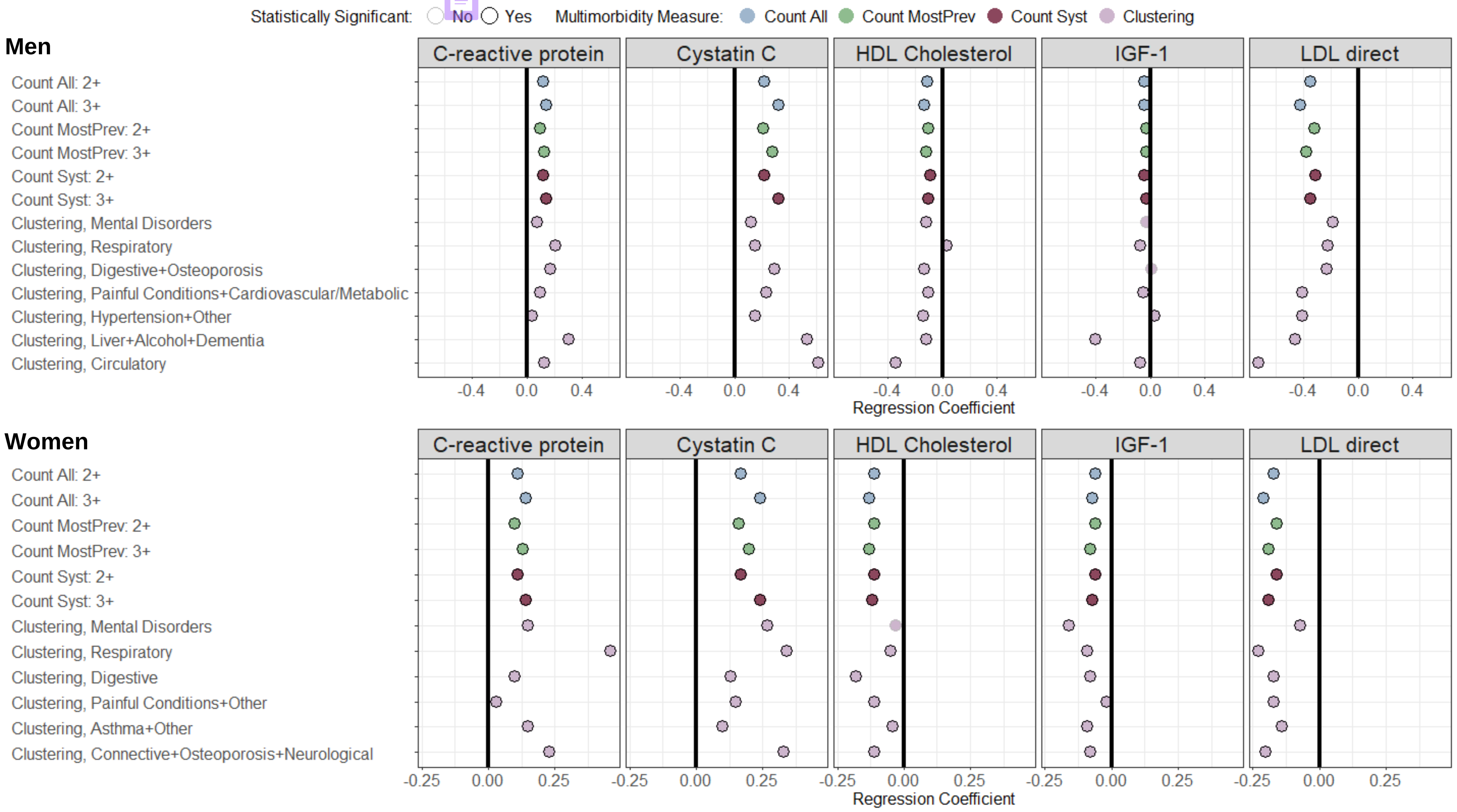

Abbreviations: Count MostPrev = 12 most prevalent conditions in the list; Count Syst = count of body-systems involved. 2+ = multimorbidity defined using 2 or more conditions; 3+ = multimorbidity defined using 3 or more conditions; HDL = High-Density Lipoprotein; IGF = Insulin-like Growth Factor; LDL = low-density lipoprotein.

Multimorbidity definitions are based on counting conditions or clustering. Count All, Count MostPrev and Count Syst, count the number of all conditions, 12 most prevalent conditions, or number of body systems involved by conditions, respectively. For count-based approaches, 2+ or 3+ thresholds were used to categorize participants as multimorbid or not. Cluster groups were identified among those multimorbid based on the Count All: 2+ Measure.
Analyses were undertaken using linear regression adjusted for age, Townsend deprivation index, ethnicity, and body mass index. Estimates represent the expected change in each blood-based measure among those with the corresponding multimorbid status, as compared to the reference group of those identified as “not multimorbid”.

Biomarkers were standardized to have mean 0 and standard deviation equal to 1, separately in men and women.
Analyses were completed among 405,654 participants with fully observed biomarker data.

Figure 2. Associations (Odds Ratio (95% Confidence Interval)) between multimorbidity measures at baseline and mortality (at 3 or 10 years of follow-up) in men and women.

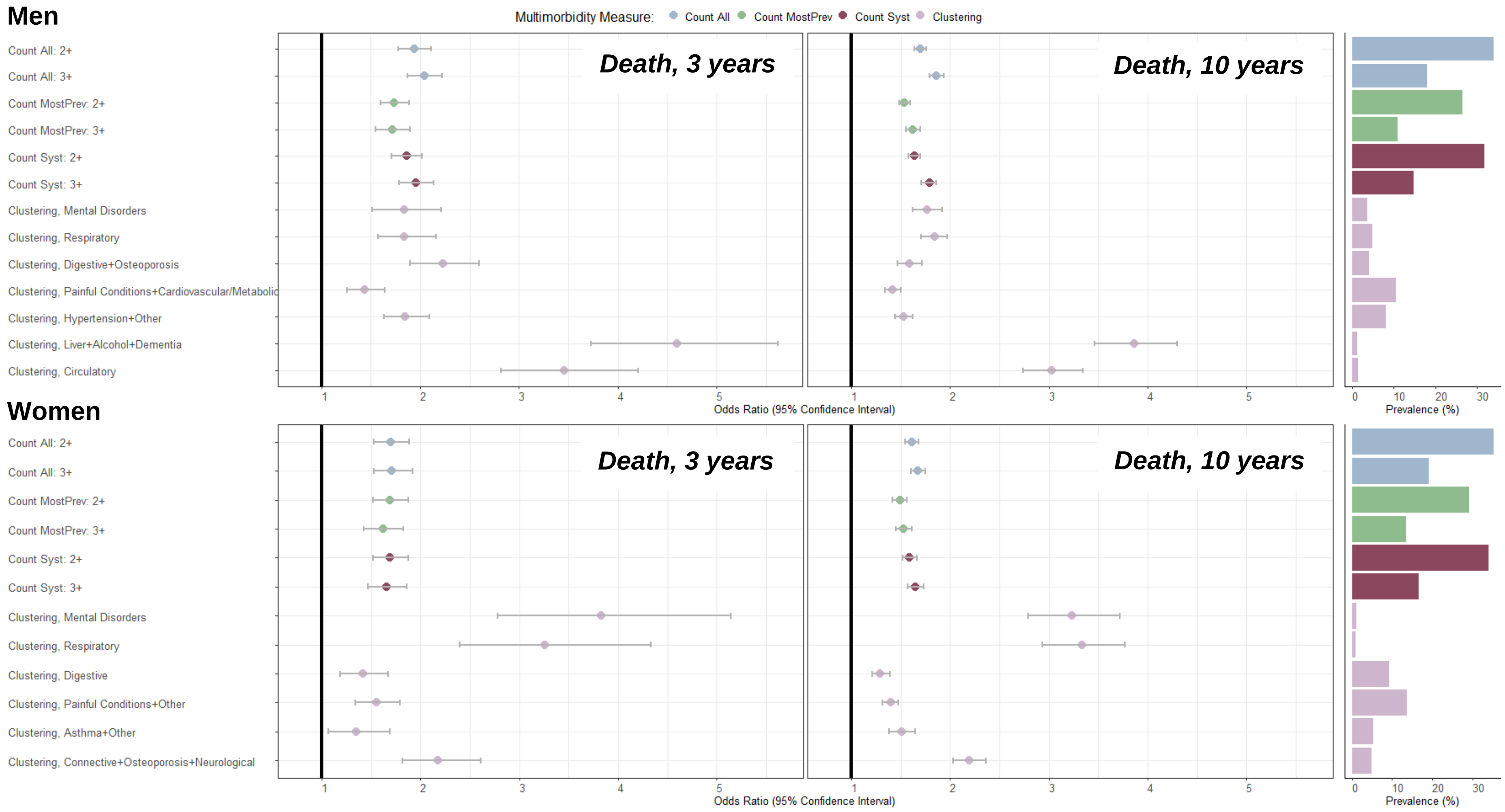

Abbreviations: Count MostPrev = 12 most prevalent conditions in the list; Count Syst = count of body-systems involved. 2+ = multimorbidity defined using 2 or more conditions; 3+ = multimorbidity defined using 3 or more conditions.

Multimorbidity definitions are based on counting conditions or clustering. Count All, Count MostPrev and Count Syst, count the number of all conditions, 12 most prevalent conditions, or number of body systems involved by conditions, respectively. For count-based approaches, 2+ or 3+ thresholds were used to categorize participants as multimorbid or not. Cluster groups were identified among those multimorbid based on the Count All: 2+ Measure.
Analyses undertaken using logistic regression models adjusted for age, Townsend deprivation index, ethnicity, and body mass index. Odds Ratios represent the odds of dying among those with the corresponding multimorbid status, as compared to the reference group of those identified as "not multimorbid".

Click here to access/download
Supporting Information
MMDefinition_UKBiobank_SupplementaryMaterial.docx

Click here to access/download
Supporting Information
MMDefinition_UKBiobank_SupplementaryMaterialClustering.docx
