# Supplementary material for: Comparison of count-based and clustering definitions of multimorbidity and their association with prevalence of multimorbidity, health profiles, and mortality: A cohort study of UK Biobank participants
Source: PLoS Med. 2026 Jun 12;23(6):e1004914. doi: 10.1371/journal.pmed.1004914 (PMC13309015; doi:10.1371/journal.pmed.1004914)
Supplement: Attachment — Submitted filename: Response_to_reviewers_final.pdf [file pmed.1004914.s005.pdf]

PLOS Medicine.
Editor

Dear Editor,

16th March, 2026

"Count-based and clustering definitions of multimorbidity: implications for prevalence, health profiles, and mortality in the UK Biobank" (PMEDICINE-D-26-00095R1)

Thank you for considering our manuscript for publication and for the positive feedback from reviewers. Please see below a point-by-point response to the reviewers' comments. The concerns raised by the reviewers are listed and followed by our responses. Changes made to the paper have also been included below each response, with an indication of their position in the manuscript.

We look forward to hearing from you.

Yours sincerely,

Benjamin Landré, on behalf of all authors
Université Paris Cité, INSERM U1153
Epidemiology of Ageing & Neurodegenerative diseases
10 Avenue de Verdun, 75010 Paris, France
Email: benjamin.landre@inserm.fr

Comments from the academic editor

I agree on the strength of the paper in terms of sample size, detailed analyses and quality of the write up. I also want to emphasize upfront that there shouldn't be expectation that this paper could be a game change. What it is showing is largely expected, and as acknowledged by the authors and previous investigators, the definition of multimorbidity is context specific and there is not that much one can do about it. For instance, HIV/AIDS not accounted for in this study would be a prominent condition if the same study was to be conducted in Africa.

OUR RESPONSE: We thank the Academic Editor for this comment but we note that much of the research on multimorbidity is not in Africa. The context-specific nature of multimorbidity definition is described in the Discussion.

The impact of multimorbidity on individuals and health systems might be relevant everywhere but particularly so in ageing populations as stated in the first line of the Abstract. The primary contribution of this manuscript is to illustrate how commonly used definitions of multimorbidity influence prevalence estimates and associations with health outcomes within the same population. The Introduction and Discussion sections have been revised extensively to better describe the contribution of our manuscript. Please see some changes to the manuscript in the box below.

Abstract, page 2, lines 32 to 36:

While most studies rely on simple counts of conditions, clustering approaches have also been used to describe patterns of co-occurring diseases. The extent to which these methodological choices affect prevalence estimates, and associations with health profiles and mortality remains unclear.

Introduction, page 3, lines 26 to 31:

To our knowledge no study has used multiple multimorbidity definitions, including count-based and cluster-based approaches, in the same population to examine how these methodological choices influence prevalence estimates and associations with health outcomes. The manner in which multimorbidity is defined has important clinical and public health implications, particularly for the identification of high risk patients, monitoring health and the implementation of healthcare strategies, and resource allocation within healthcare systems.

Discussion, page 10, lines 2 to 4:

This study examined how commonly used operational definitions of multimorbidity, including count-based definitions and a cluster-based approach, affect estimates of multimorbidity

prevalence estimates and their associations with health and risk of mortality within the same study population.

Reviewer #1

This manuscript compares count-based and clustering-based definitions of multimorbidity in a large population-based cohort (UK Biobank) and evaluates how these definitions influence estimates of prevalence and associations with health outcomes and mortality. The topic is important, as multimorbidity is increasingly used in epidemiological and clinical research, yet operational definitions remain heterogeneous and can materially affect inference. The use of a single large dataset to implement multiple commonly used definitions in parallel is a clear strength. Overall, the analytical approaches are conventional and competently executed. However, several issues related to clarity of framing, comparability of definitions, interpretation of effect sizes, and handling of missing data merit attention.

OUR RESPONSE: We thank the reviewer for their detailed and constructive feedback; please see our response to all comments below.

Comment 1: The authors state that few studies have simultaneously evaluated count-based and clustering approaches to defining multimorbidity in large-scale datasets. This claim would benefit from explicit citations to prior work that has addressed this question, to better contextualize the novelty.

OUR RESPONSE: We appreciate this suggestion and have added additional references to studies that have examined multimorbidity definitions and clustering approaches in large datasets; please see below changes to the manuscript and new references.

**Introduction, page 3, lines 24 to 26:**

While previous studies have discussed the strengths and limitations of count-based versus clustering approaches to defining multimorbidity,8 few have used both approaches simultaneously in large scale studies.22,23

Added references:

8. Skou ST, Mair FS, Fortin M, et al. Multimorbidity. *Nature reviews Disease primers* 2022; **8**(1): 48.

22. Littlejohns TJ, Liu W, Calvin CM, et al. Multimorbidity, disease clusters and risk of all-cause and cause-specific mortality: a population-based prospective cohort study. *Sci Rep* 2025; **15**(1): 41393.

23. Calvin CM, Conroy MC, Moore SF, Kuzma E, Littlejohns TJ. Association of Multimorbidity, Disease Clusters, and Modification by Genetic Factors With Risk of Dementia. *JAMA network open* 2022; **5**(9): e2232124.

Comment 2: The introduction focuses on heterogeneity in multimorbidity definitions and prevalence estimates, but could more explicitly state why this matter, e.g., different definitions may identify different populations at risk and therefore influence inference about health outcomes, resource allocation, and intervention design.

OUR RESPONSE: We agree and have revised the Introduction to better describe the implications of heterogeneity in multimorbidity definitions, please see below and our response to the Academic Editor.

Introduction, page 3, lines 28 to 31:

The manner in which multimorbidity is defined has important clinical and public health implications, particularly for the identification of high risk patients, monitoring health and the implementation of healthcare strategies, and resource allocation within healthcare systems.

Comment 3: The clustering approach is not directly comparable to the count-based definitions, as it is applied only within participants already classified as multimorbid and thus

describes heterogeneity within a selected subgroup rather than a population-wide definition. I would suggest reframing clustering as an analysis of multimorbidity subtypes, and avoiding direct comparisons of prevalence or effect sizes with count-based definitions.

OUR RESPONSE: We agree; the manuscript has been revised to remove any notion of comparison while also describing differences within count measures as conditions and affected body systems is not the same. Each of these definitions is better described in the methods section and discussed in paragraphs 2 and 3 of the Discussion section. We feel that reporting the characteristics of groups identified by each approach, including prevalence and associations with outcomes, provides valuable insight into practical consequences for clinical or intervention purposes given the limited discussion on these issues in the current literature.

Abstract, page 2, lines 9 to 12:

Using UK Biobank data (n=474,397), we constructed six count-based definitions of multimorbidity based on three list of conditions (long list of 38 conditions, 12 most prevalent, or number of affected body systems) and two thresholds (2+ and 3+ conditions). We also used clustering analysis to create subgroups with similar conditions within a cluster.

Methods, page 5, lines 1 to 2:

The first step consisted of elaboration of seven definitions of multimorbidity, six count-based definitions and one based on clustering analysis.

Methods, page 5, lines 18 to 21:

Subsequent analyses using the six multimorbidity definition and the clustering approach were composed of (1) estimation of multimorbidity prevalence for each definition, analyses on associations of each multimorbidity definition with (2) health profiles (concurrent measures of health and blood-based measures) and (3) mortality risk at the 3- and 10-year horizon.

Comment 4: Logistic regression is used for all binary outcomes, including mortality at fixed 3- and 10-year periods. While this is acceptable, it implicitly assumes comparable follow-up and does not account for censoring in a time-to-event framework. The authors should clarify the rationale for this choice (over survival analysis).

OUR RESPONSE: We have revised the Methods section to clarify our strategy. As our objective was to estimate the association between multimorbidity and survival probability at clinically relevant fixed time horizons (3 and 10 years), we used logistic regression at each time point rather than a Cox proportional hazards model that provides an average hazard ratio over the entire follow-up. Our approach directly addresses clinical impact over a given timeframe rather than proportional risk. In the revised manuscript we added a table showing hazard ratios (95% CI) across multimorbidity definitions (Table S11); the conclusions remain unchanged, although the clinical interpretation using a Cox model is less straightforward.

Methods, page 5, lines 33 to 37:

The association of multimorbidity (all definitions) with mortality was examined at two different clinically relevant fixed horizon, 3 and 10 years of follow-up, using multivariable logistic regression, adjusting for sociodemographic factors and BMI. We chose the 3- and 10-year time horizon in the main analyses to examine short- and long-term risk rather than assuming risk to be constant over time. In additional analysis, we also provide estimates of risk using a Cox proportional hazards model over the total follow-up period (end of follow-up November 2022).

Results, page 9, lines 13 to 15:

Additional analysis, using Cox regression over the full follow-up period (median=13.7 years, IQR= 12.9 to 14.4) yielded results (**Table S11**) that were broadly similar to that in the main analyses.

Comment 5: A relatively large number of participants are excluded due to missing covariates, outcomes, or biomarker data, yet there is no exploration of missingness patterns. Given that missingness in UK Biobank is often health-related, the authors should at minimum report whether excluded participants differed in age or multimorbidity burden, and acknowledge this as a limitation in the discussion.

OUR RESPONSE: Thank you for this comment. We have added a table comparing participants included and excluded from the analyses (Table S3) and described it in the results section and as a limitation in the discussion section.

Results, page 7, lines 4 to 6:

The participants excluded from the analyses (N=5815, 1.2%) had a poorer health profile and were more likely to die during the follow up (Table S3).

Discussion, page 12, lines 2 to 3:

Furthermore, participants who were excluded from the present analyses due to missing data were in poorer health, although the small size of this group (1.2%) is unlikely to affect results.

Comment 6: Minor point: The term "multivariate logistic regression" is used throughout. Strictly speaking, these models are "multivariable" (single outcome with multiple covariates), rather than "multivariate" (multiple outcomes).

OUR RESPONSE: We have corrected this error in the revised manuscript. Thank you.

Comment 7: The observed differences in prevalence across count-based definitions/thresholds are expected and internally consistent. However, language such as "varied significantly as a function of the multimorbidity definition" could be misinterpreted as inferential, despite no formal hypothesis testing being conducted.

OUR RESPONSE: We agree, we have revised the manuscript to avoid inappropriate causal interpretation. Thank you for your suggestion.

Results, page 7, lines 12 to 12:

The prevalence of multimorbidity varied substantially across the definitions of multimorbidity and sex (**Table 1**).

Results, page 8 lines 26 to 27:

The estimated coefficients for association of some clusters with blood-based measures were notably larger than those estimated using count-based definitions of multimorbidity.

Discussion, page 10, lines 8 to 10:

We found the threshold used to define multimorbidity had a substantial impact on estimated prevalence but its influence on associations with concurrent health measures, including blood-based measures and mortality risk was more limited.

Discussion, page 11, lines 26 to 27:
our findings suggest that use of a higher threshold on the number of chronic conditions is not necessarily linked to higher mortality risk

Comment 8: Larger effect sizes for both clinical outcomes and biomarkers are observed for smaller, more severe clusters and more restrictive thresholds, which is expected given overlap between exposure definitions and outcomes and largely reflects disease composition rather than superior performance of specific definitions. The Discussion should therefore avoid interpreting effect size differences as evidence that clustering or particular definitions better identify high-risk groups or better capture health complexity (e.g., statements that the clustering approach "better identify patients with high risk," "enable identification of specific subgroups with higher risk," or "better capture the complexity of health profiles"), and instead frame these findings descriptively.

OUR RESPONSE: We have revised the Abstract and Discussion to state that these differences reflect expected consequences of methodological choices in identifying multimorbid individuals.

Abstract, page 2, lines 28 to 32:
The prevalence of multimorbidity in a given populations is highly dependent on how it is defined. For count-based definitions we did not find evidence to suggest that a 3+ count was associated with higher mortality risk than a 2+ count. An alternative to these approaches is the clustering method that identifies groups with co-occurring conditions, with some groups at particularly high risk of adverse health outcomes. These findings highlight the importance of defining multimorbidity clearly, in accordance with the objectives of the study.

Discussion, page 10 to 11, lines 29 to 2:
An important aspect of our study was to extend the analyses beyond the commonly used count based measures of multimorbidity to also include a clustering approach. This approach aims to

characterize patterns of co-occurring conditions in the study population and identify subgroups of individuals with similar disease profiles.19,20 While the count-based approach is agnostic to correlation patterns in diseases in the population, the clustering approach seeks to exploit these correlations by creating groups of participants who are similar within each cluster and different across clusters. Our results show clusters to differ substantially in both prevalence and health profiles, reflecting the underlying combinations of diseases that characterize each cluster. In particular, clusters that had a low prevalence were often composed of more severe or complex combinations of conditions, and participants in these clusters had a more adverse health profile and higher risk of mortality.

Comment 9: An important finding is the relative robustness of mortality associations across count-based definitions, despite large differences in prevalence. This result could be emphasized more clearly. In contrast, the large odds ratios observed for low-prevalence clusters should be interpreted in light of severity, small group size, and differences in baseline risk, rather than as evidence of superior predictive performance.

OUR RESPONSE: We thank the reviewer for this suggestion. We have revised the Results and Discussion sections accordingly.

Discussion, page 10, lines 8 to 14:

We found the threshold used to define multimorbidity had a substantial impact on estimated prevalence but its influence on associations with concurrent health measures, including blood-based measures and mortality risk was more limited. This was also the case for the Count Syst approach that defined multimorbidity as at least 2 and at least 3 conditions across different body systems. Taken together, these findings suggest that while the number and type of conditions included in count-based multimorbidity definitions strongly affect estimates of disease burden, their influence on the association between multimorbidity and mortality may be more modest.

Comment 10: Given the strong age gradients observed across definitions and clusters, stratified summaries or age-standardized prevalence estimates could help disentangle differences due to definition choice from differences driven primarily by age.

OUR RESPONSE: We agree that age is a key determinant of multimorbidity prevalence. To make this clear to the reader we calculated the prevalence of multimorbidity in age subgroups defined using terciles, shown in new Table S4.

Methods, page 5, lines 23 to 25:

We also reported the prevalence of multimorbidity by age-groups defined using terciles (38-53, 53-61 and 61-73 years old).

Results, page 7 to 8, lines 34 to 2:

For all definitions, the prevalence of multimorbidity increased with age (**Table S4**). Between the ages of 38-53 and 61-73, it increased from 20.2% to 46.2% in men and from 25.9% to 44.4% in women for the *Count All: 2+* definition. The largest relative increase across the age-groups was observed in the circulatory cluster in men and in the connective + osteoporosis + neurological cluster in women. The only exception concerns the ‘Mental disorders’ cluster in both men and women, where prevalence was relatively stable across the age-groups.

Comment 11: The discussion accurately summarizes the main descriptive findings, but at times risks over-interpreting variability in effect sizes as evidence that clustering is inherently superior to count-based approaches. Given the conditional nature of clustering and differences in scale, such comparisons should be framed more cautiously.

The claim that clustering "better captures complexity" and "identifies vulnerable groups" is plausible, but should be noted that clustering identifies smaller, less heterogeneous groups by construction, and that generalizability remains challenging.

OUR RESPONSE: Thank you for these valuable comments; we agree, and have amended the text in the discussion to avoid misinterpretation of results suggesting that one definition is inherently “better” than another.

Abstract page 2 lines 28 to 32:

The prevalence of multimorbidity in a given populations is highly dependent on how it is defined. For count-based definitions we did not find evidence to suggest that a 3+ count was associated with higher mortality risk than a 2+ count. An alternative to these approaches is the clustering method that identifies groups with co-occurring conditions, with some groups at particularly high risk of adverse health outcomes. These findings highlight the importance of defining multimorbidity clearly, in accordance with the objectives of the study.

Introduction, page 3 lines 20 to 23:

An alternative to a count-based approach is clustering that aims to identify patterns of co-occurring conditions so that individuals within a cluster have a similar disease profile. This approach is thought to be informative on understanding shared risk factors and has potential implications for patient management.[19,20] [7,21,22]

Discussion, page 10 to 11 lines 29 to 2:

An important aspect of our study was to extend the analyses beyond the commonly used count based measures of multimorbidity to also include a clustering approach. This approach aims to characterize patterns of co-occurring conditions in the study population and identify subgroups of individuals with similar disease profiles.19,20 While the count-based approach is agnostic to correlation patterns in diseases in the population, the clustering approach seeks to exploit these correlations by creating groups of participants who are similar within each cluster and different across clusters. Our results show clusters to differ substantially in both prevalence and health profiles, reflecting the underlying combinations of diseases that characterize each cluster. In particular, clusters that had a low prevalence were often composed of more severe or complex combinations of conditions, and participants in these clusters had a more adverse health profile and higher risk of mortality.

Reviewer #2

Thank you for the opportunity to review this manuscript. Authors have put in a lot of effort to examine different definitions of multimorbidity across prevalence and selected health outcomes.

This manuscript is mostly well-written, with opportunities to improve clarity, which I have highlighted in the annotated document (please see attachment).

OUR RESPONSE: Thank you for the positive feedback and the annotated document. Please see our response to all comments below, the annotated comments (starting with comment 2 below) and our response to each comment are listed below.

Comment 1: In its current form, the methods section needs justification for the chosen clustering technique and a clearer description of the clustering analysis. More discussion on the unexpected findings and strength and limitations of the clustering technique.

OUR RESPONSE: The 'Methods' section refers to a supplement specific to cluster analysis. We have also added text to the methods section in the manuscript to allow the reader to follow the clustering approach. This approach is similar to that used by Violan et al., published in PLOS One and cited more than 1,418 times (as of March 16 2026). We refer to this paper in the manuscript (reference 34) and provide further detail in the methods supplement. In response to this comment, we have added several points on the methodological choices specific to this approach in the revised manuscript.

**Method, page 5, lines 11 to 17:**<br><br>For the *clustering approach*, we used the method proposed by Violan et al[34] separately in men and women with multimorbidity defined as having at least two of the all conditions (as in the ***Count All* approach**). The analyses consisted of a two-step procedure where a multiple correspondence analysis was followed by k-means clustering, a non-hierarchical cluster analysis that is less susceptible to outliers in the data. The aim of this method is to generate clusters where homogeneity is maximized within a cluster and heterogeneity maximized across clusters. Further details of the clustering method are provided in **Supplementary Methods: Cluster Analyses**.<br><br>**Discussion, page 10 to 11 lines 29 to 2:**<br>An important aspect of our study was to extend the analyses beyond the commonly used count based measures of multimorbidity to also include a clustering approach. This approach aims to

characterize patterns of co-occurring conditions in the study population and identify subgroups of individuals with similar disease profiles.19,20 While the count-based approach is agnostic to correlation patterns in diseases in the population, the clustering approach seeks to exploit these correlations by creating groups of participants who are similar within each cluster and different across clusters. Our results show clusters to differ substantially in both prevalence and health profiles, reflecting the underlying combinations of diseases that characterize each cluster. In particular, clusters that had a low prevalence were often composed of more severe or complex combinations of conditions, and participants in these clusters had a more adverse health profile and higher risk of mortality.

Discussion, page 11, lines 31 to 33:

A perfect method to define multimorbidity clusters does not exist but the method we followed,[34] based on k-means clustering, is widely used and a statistically robust method. We chose this method as it allows evaluation of cluster composition through multiple indicators.

Comment 2: “don't quite agree with this statement which is quite loaded. Multimorbidity simply means having multiple morbidities. Please qualify this statement more clearly.”; the highlighted text is : “the lack of standardization in defining multimorbidity contributes to substantial variability in prevalence estimates and associations with health outcomes”.

OUR RESPONSE: We have amended the text accordingly.

Introduction, page 3, lines 8 to 9:

Besides differences in study populations, lack of attention to the definition of multimorbidity used contributes to substantial variability in prevalence estimates and may influence their respective associations with health outcomes.

Comment 3: “please be clear what do you mean by the affected body systems in the counts of conditions.”; the highlighted text is: “the affected body systems”.

OUR RESPONSE: We have amended the sentence to avoid any misunderstanding.

Introduction, page 3, lines 17 to 20:

In addition to counting individual conditions, some studies use counts of affected body systems. Count-based approaches generally use a threshold of two or more conditions to define multimorbidity. A higher count threshold, such as three or four conditions, identify multimorbid populations with greater needs.[11,16,17]

Comment 4: "See a published article in this topic: Patterns of multi-morbidity and prediction of hospitalisation and all-cause mortality in advanced age. Age Ageing. 2018;47(2):261-8."; the highlighted text is : "To our knowledge, no study has directly compared the effect of count-based and clustering multimorbidity measurements on associations between multimorbidity and health profiles".

OUR RESPONSE: There appears to be some confusion as the paper suggested by the reviewer study examines clusters of multimorbidity among 421 Māori (aged 80–90) and 516 non-Māori (aged 85), and their association with mortality. This paper does not compare different definitions of multimorbidity.

Comment 5: "number of medications"; the comment is in the covariates paragraph.

OUR RESPONSE: Polypharmacy is described in the paragraph '**Concurrent health outcomes, blood-based measures and mortality**' alongside other concurrent health outcomes examined in the manuscript.

Comment 6: "state the clustering technique here", highlighted text is "we replicated the method proposed by Violan et al separately in men and women".

OUR RESPONSE: The clustering method comprises several steps, that are now described in the revised version of the manuscript. We also refer to a more detailed methodological supplement that contains all the necessary information.

Comment 7: "Please include justification how this method was chosen over other clustering techniques."; the comment is put at the end of the 1st paragraph of statistical analyses.

OUR RESPONSE: A discussion on our choice has been added to the discussion alongside considerations for choice of definitions in the revised manuscript. Please also see our response to comment 1 above.

**Discussion, page 11, lines 31 to 33:**
A perfect method to define multimorbidity clusters does not exist but the method we followed,[34] based on k-means clustering, is widely used and a statistically robust method. We chose this method as it allows evaluation of cluster composition through multiple indicators.

Comment 8: "k-means clustering is not suitable for binary outcomes, i.e. present/absent of a condition."; the comment is at the end of the 1st paragraph of statistical analyses.

OUR RESPONSE: We used a method that has been used before, that comprises two stages: a multiple component analysis and k-means clustering. Multiple component analysis is a data reduction method that is used for categorical data (similar to PCA for continuous data) and results in a continuous matrix that is then input into k-means clustering. So we are not applying k-means clustering to binary outcomes. Two recent reviews (references below) indicate that this approach (multiple component analysis and k-means clustering) is among the most commonly used in the literature on multimorbidity. Please also see our response to comment 1 above.

References:

Xi J, Miao M, Li PWC, Yu DSF. Prognostic effects of multimorbidity clusters on health outcomes in adults: A systematic review and meta-analysis. *Ageing Res Rev* 2025; **112**: 102897.

Skou ST, Mair FS, Fortin M, et al. Multimorbidity. *Nature reviews Disease primers* 2022; **8**(1): 48.

Violan C, Roso-Llorach A, Foguet-Boreu Q, et al. Multimorbidity patterns with K-means nonhierarchical cluster analysis. *BMC Fam Pract* 2018; **19**(1): 108.

Comment 9: "describe the process for ensuring the stability of the cluster."; the comment is at the end of the 1st paragraph of statistical analyses, as previous comment.

OUR RESPONSE: Please see the response to the previous comment and comment 1. The method used by Violan et al., which is used in our manuscript, does not include the step suggested by the reviewer.

Comment 10: "move this sentence up to before "details on the clustering methods..."; highlighted text is "Briefly, we performed a multiple correspondence analysis followed by k-means clustering on the resulting dimensions".

OUR RESPONSE: Please see our response to comment 6.

Comment 11: "is this is the most common combination for men?"; highlighted text is "liver + alcohol + dementia (1.2%), circulatory (1.4%), mental disorders (3.7%), digestive + osteoporosis (4.1%), respiratory (4.9%), hypertension + other (8.2%), and painful conditions + cardiovascular and metabolic disorders (10.5%)".

OUR RESPONSE: Yes, it was present in 10.5% of men - the largest cluster in terms of size.

Comment 12: "report the most common combinations of conditions here."; highlighted text is "Supplementary Methods: Cluster Analyses. There was greater variation in mean ages and condition counts using the clustering approach, compared to multimorbidity definitions based on counts".

OUR RESPONSE: The combinations are described in the sentence preceding the sentence underlined by the reviewer. Additional information "Supplementary Methods: Cluster Analyses" in the underlined section also includes complete detail on all these combinations.

Comment 13: "3 or more conditions? Please be clearer"; highlighted text is "For those considered multimorbid, the mean number of conditions varied between 2.5 (hypertension + other) and 4.9 (circulatory) in men and between 2.7 (painful conditions + other) and 4.2 (respiratory) in women."

OUR RESPONSE: We have reworded the sentence as shown below to avoid ambiguity.

**Results, page 7, line 20 to 22:**<br>The *clustering approach* (**Supplementary Methods: Cluster Analyses**), that grouped multimorbid participants into clusters with similar conditions with multimorbidity defined using the *Count All: 2+* approach (n=73,362 men and 91,261 women) identified seven clusters in men and six in women.

Comment 14: "Have to explain in the method section justification for these arbitrary cutoff?"; highlighted text is "Definitions using a threshold of three conditions to define multimorbidity yielded stronger associations compared to those using a threshold of two".

OUR RESPONSE: This point is described in the introduction. We have also added a sentence and a reference to clarify this in revised version of the methods section.

**Methods, page 5, line 10:**<br>These are commonly used thresholds in research on multimorbidity.[15]

Cited reference:

Ho IS, Azcoaga-Lorenzo A, Akbari A, et al. Examining variation in the measurement of multimorbidity in research: a systematic review of 566 studies. *Lancet Public Health* 2021; **6**(8): e587-e97.

Comment 15: "what is the pattern of condition in this cluster?"; highlighted text is "These ORs were significantly higher than those for men in the painful conditions + cardiovascular and metabolic disorders cluster, that had an OR (95% CI) of 1.42 (1.34, 1.50) and was the most prevalent cluster."

OUR RESPONSE: Please see our response to comments 11 and 12.

Comment 16: "Is this statement supported by the study results? earlier paragraph the authors emphasize the measurements of multimorbidity should be context specific., as in the subsequent statement."; highlighted text is "Given the expected increase in multimorbidity prevalence linked to global aging and its impact on health, our results emphasize the need for consensus on definition and measurement of multimorbidity."

OUR RESPONSE: Thank you for this comment, we have removed the text referring to need for consensus. Indeed, the definition of multimorbidity ought to depend on the purpose of the study. This has been included in the revised manuscript. We feel that the choice of a definition ought to be informed and deliberate, with the objective of the exercise, please see below the text in the revised manuscript.

Discussion, page 12, lines 12 to 17:

Given the expected increase in multimorbidity prevalence linked to global aging and its impact on health, our results emphasize the need for providing a clear definition of multimorbidity, in line with the aim of the study. The choice of a definition impacts key epidemiological indicators and depends on both the research or clinical context, as well as the availability of data. It might be useful for studies to carefully consider and justify their choice of definition when designing studies or interventions, as without such transparency, the potential for translational research on multimorbidity may remain limited.

Comment 17: “please check legend.”; figure 1 is highlighted.

OUR RESPONSE: We have checked the legend, thank you.

Reviewer #3

The paper is quite interesting and has the potential to fill some important gaps in the multimorbidity literature. However, there are some important points to consider before moving to a final version of the paper. The points are listed below. Depending on the answers and changes based on them, the paper can be evaluated again to assess its potential for publication.

OUR RESPONSE: Thank you for the positive feedback and we hope to have addressed your concerns in our response to comments below.

Comment 1: The rationale of the study is too broad. What is the main gap in the literature that authors intend to fill?

OUR RESPONSE: Thank you; we revised the introduction and discussion sections to set out our objectives and the gap in the literature.

Abstract, page 2, lines 33 to 36:

While most studies rely on simple counts of conditions, clustering approaches have also been used to describe patterns of co-occurring diseases. The extent to which these methodological choices affect prevalence estimates, and associations with health profiles and mortality remains unclear.

Introduction, page 3, lines 26 to 31:

To our knowledge no study has used multiple multimorbidity definitions, including count-based and cluster-based approaches, in the same population to examine how these methodological

choices influence prevalence estimates and associations with health outcomes. The manner in which multimorbidity is defined has important clinical and public health implications, particularly for the identification of high risk patients, monitoring health and the implementation of healthcare strategies, and resource allocation within healthcare systems.

Discussion, page 10, lines 2 to 4:
This study examined how commonly used operational definitions of multimorbidity, including count-based definitions and a cluster-based approach, affect estimates of multimorbidity prevalence estimates and their associations with health and risk of mortality within the same study population.

Comment 2: Why compare clustering-based approaches and count-based measures in terms of prevalence? These two measures are not comparable by their construction. Is it fair to do this comparison?

OUR RESPONSE: We agree with the reviewer and have described this in the revised manuscript. Despite conceptual differences, these approaches are both widely used in multimorbidity research without explicit consideration of the impact it has on results. We use one large cohort study to demonstrate that the choice of multimorbidity definition affects prevalence and estimates of impact on health outcomes. Please also see our response to Reviewer #1, comments 11 and 12.

Comment 3: Would a cox-regression analysis be a more appropriate method for assessing the association between multimorbidity and mortality? Hazard ratios are considered more robust for time-to-event data than odds ratios. Why the authors prefer to use logistic regression?

OUR RESPONSE: Please see the response to Reviewer #1 comment 4.

Comment 4: The choice of clustering methods is not discussed. Why is this approach more useful than others? Some systematic review about the topic found other techniques used for clustering of multimorbidity.

OUR RESPONSE: Please see the response to Reviewer #2 comment 1.

Comment 5: Cross-sectional analysis with blood markers is not explained. Is it possible to conduct this assessment using longitudinal data on the outcomes?

OUR RESPONSE: We used blood-based measures to provide an additional descriptive characterization of the health profiles associated with different multimorbidity definitions. These analyses were cross-sectional as the objective was descriptive to show that different operational definitions of multimorbidity are associated with distinct biological profiles. We have added the following text to make this clear.

**Methods, page 5, lines 30 to 32:**<br>We compared biological profiles using objective blood-based measures in participants with and without multimorbidity using multivariable linear regression models adjusted for the same set of covariates.

Comment 6: There is no clear explanation of how falls were measured or how the Townsend Deprivation Index was derived. Furthermore, authors did not state (or at least reference) how the blood biomarkers were collected in the UKB.

OUR RESPONSE: Apologies. The Townsend index is a common measure of socio-economic deprivation in the United-Kingdom; we have some description and a reference to the revised manuscript. Falls were described in the method section as being self-reported.

**Method, page 4, line 19:**<br>reported falls in the past year

**Method, page 4, lines 31 to 32:**<br>Townsend deprivation index (a relative indicator of material deprivation widely used to measure socio-economic circumstances),[33]

Added reference:

Lloyd CD, Norman PD, McLennan D. Deprivation in England, 1971-2020. *Applied spatial analysis and policy* 2023; **16**(1): 461-84.

Comment 7: Residual confounding might bias results because of important confounders that were omitted in the analysis, including lifestyle factors such as smoking, alcohol consumption, and physical activity, which are known predictors of MM and are strongly associated with most of the outcomes and biomarkers assessed in the study.

OUR RESPONSE: We agree that lifestyle factors are important determinants of multimorbidity and related outcomes. However, the aim of our study was not to estimate causal effects but to examine how different operational definitions of multimorbidity influence prevalence estimates, participant characteristics, and associations with health outcomes. Assessing the causal role of lifestyle factors would require a different study design and is beyond the scope of the present analyses. We have nevertheless acknowledged this point in the Discussion as a potential avenue for future research.

**Discussion, page 12, lines 8 to 11:**<br>A related limitation is that we did not set out to examine the explanations linking multimorbidity and health status or mortality. It is possible that a range of social, behavioural, and biological risk factors play an important role; further longitudinal studies are needed to clarify their role.

Comment 8: The authors made some contradictory statements. In the abstract, the authors stated that "Higher thresholds identified more severe health profiles on all measured health outcomes, blood-based measures but not higher mortality risks", but deviated a bit from this in their discussion: "We also found that while the choice of cut-off used for defining who was multimorbid

had a large effect on the estimated prevalence's of multimorbidity, it had a smaller impact on associations with concurrent health outcomes, blood-based measures and mortality risk".

OUR RESPONSE: Apologies, we revised the abstract to avoid possible misunderstanding. Thank you.

Comment 9: The majority of these points are not addressed in the limitations section of the paper.

OUR RESPONSE: We have revised the manuscript carefully, including the limitations section.
