# Supplementary material for: Comparison of count-based and clustering definitions of multimorbidity and their association with prevalence of multimorbidity, health profiles, and mortality: A cohort study of UK Biobank participants
Source: PLoS Med. 2026 Jun 12;23(6):e1004914. doi: 10.1371/journal.pmed.1004914 (PMC13309015; doi:10.1371/journal.pmed.1004914)
Supplement: Attachment — Submitted filename: Answer_22May.docx [file pmed.1004914.s006.docx]

|  |  |  |
| --- | --- | --- |

**PLOS Medicine.**

**Editor**

Dear Editor,

19^th^ May, 2026

**“Comparing count-based and clustering definitions of multimorbidity: implications for prevalence, health profiles, and mortality, a prospective cohort study in the UK Biobank" (PMEDICINE-D-26-00095R1)**

Thank you for considering our manuscript for publication and for the positive feedback. Please see below a point-by-point response to general comments. The concerns raised by the reviewers are listed and followed by our responses. Changes made to the paper have also been included below each response, with an indication of their position in the manuscript.

We look forward to hearing from you.

Yours sincerely,

Benjamin Landré, on behalf of all authors

Université Paris Cité, INSERM U1153

Epidemiology of Ageing & Neurodegenerative diseases

10 Avenue de Verdun, 75010 Paris, France

Email: [benjamin.landre@inserm.fr](mailto:benjamin.landre@inserm.fr)

**Comments from Reviewers:**

**Reviewer #1:** Thank you to the authors for their careful and thorough revision of the manuscript. The responses to the previous comments are appropriate and have substantially improved the clarity and interpretation of the paper.
I have no further comments and am satisfied with the revisions. The manuscript is methodologically sound, and I defer to the editor regarding its priority for the journal.

We thank you for your comments and helpful review of our manuscript.

**Reviewer #3:** The authors addressed all the comments and adjusted most of the paper accordingly. I am just still concerned about the conclusions in the abstract section. They seem so general and do not provide any specific conclusions for future studies. The last sentence, "These findings highlight the importance of defining multimorbidity clearly, in accordance with the objectives of the study," is too generic and unspecific.

Thank you for your comments and constructive feedback on this manuscript. We have revised the last sentence of the abstract to reflect your final recommendation.

**Abstract, page 2, lines 63-65:**

Future translational studies are warranted to determine how multimorbidity definitions can be optimized to ultimately improve clinical management and health outcomes in practice.

**General editorial requests:**
(Note: not all will apply to your paper, but please check each item carefully)

* We ask every co-author listed on the manuscript to fill in a contributing author statement, making sure to declare all competing interests. If any of the co-authors have not filled in the statement, we will remind them to do so when the paper is revised. If all statements are not completed in a timely fashion this could hold up the re-review process. If new competing interests are declared later in the revision process, this may also hold up the submission. Should there be a problem getting one of your co-authors to fill in a statement we will be in contact. Please do not add or remove authors without first discussing this with the handling editor. You can see our competing interests policy here: <https://spam.inserm.fr/fmlurlsvc/?fewReq=:B:JVA0Pzs7NCh4MzwgPihnajM+PzQ+Pyh9Z2lgb3p7fGszPTtqPjc8az5vPD0/Nj5qPT02bzc/bDg2PD89PzprOz9saD43OG1tOyh6Mz85OTY4ODg2Njoof2dqMzg6SjdvNmpUPj0/Njk7Izg6SjdvNmpsPj0/Njk7KHxtfnozbGtgZG9jZ2AgYm9ganxrTmdgfWt8YyBofChtMz0+KGZqYjM+&url=http%3a%2f%2fjournals.plos.org%2fplosmedicine%2fs%2fcompeting-interests> .

We added the section on contributions, provided more details on funding, and took the opportunity to include a thank-you note to the cohort participants.

**Page 13, lines 695-714:**

**Contribution statement**

**Gabriella C SILVA**: Conceptualization, methodology, software, formal analysis, data curation, writing original draft and editing, visualization; **Aurore FAYOSSE**: software, validation, data curation, writing – review & editing; **Louis JACOB**: validation, writing – review & editing; **Séverine SABIA**: validation, writing – review & editing, funding acquisition; **Archana SINGH-MANOUX**: validation, writing – review & editing, , funding acquisition; **Benjamin LANDRÉ**: Conceptualization, methodology, software, formal analysis, data curation, writing original draft and editing, funding acquisition, supervision.

**Competing interests**

The authors have declared that no competing interests exist.

**Acknowledgment**

This project is funded by a grant from “France 2030 ANR-23-PAVH-0006” (https://ppr-autonomie.com/le-ppr-autonomie/) , by IReSP/CNSA as part of the 2023 call for projects for the research support program “Older people, people with disabilities at all stages of life, relatives, and professionals,” supported by the National Solidarity Fund for Autonomy (CNSA) “AAP-2023-APAOB-318115” (https://iresp.net/appel_a_projets/appel-a-projets-2023-analyse-des-politiques-de-lautonomie-de-loffre-medico-sociale-et-des-besoins-des-publics-apaob-session-1/), Fondation vaincre Alzheimer “FR-23063CB” (https://www.vaincrealzheimer.org/) et fondation Alzheimer “1143650” (<https://www.fondation-alzheimer.org/en/>).

We would also like to thank the participants in the UK Biobank cohort, as well as everyone involved in the data collection.

* Please upload any figures associated with your paper as individual TIF or EPS files with 300dpi resolution at resubmission; please read our figure guidelines for more information on our requirements: <https://spam.inserm.fr/fmlurlsvc/?fewReq=:B:JVA0Pzs7NCh4MzwgPihnajM+PzQ+Pyh9Z2lgb3p7fGszOTo5bzZqPW05aj5oOTloN2s+O2g+PWw7P2g8Pzw+Nmo+Omw3bD0/bCh6Mz85OTY4ODg2Njoof2dqMzg6SjdvNmpUPj0/Njk7Izg6SjdvNmpsPj0/Njk7KHxtfnozbGtgZG9jZ2AgYm9ganxrTmdgfWt8YyBofChtMz0+KGZqYjM+&url=http%3a%2f%2fjournals.plos.org%2fplosmedicine%2fs%2ffigures> . While revising your submission, we strongly recommend that you use PLOS's NAAS tool (https://spam.inserm.fr/fmlurlsvc/?fewReq=:B:JVA0Pzs7NCh4MzwgPihnajM+PzQ+Pyh9Z2lgb3p7fGszPztrNjs6ajs+ODc7Nz8+azo5PTltPzttbzs+aGg4aDg3Ozc6NmttaCh6Mz85OTY4ODg2Njoof2dqMzg6SjdvNmpUPj0/Njk7Izg6SjdvNmpsPj0/Njk7KHxtfnozbGtgZG9jZ2AgYm9ganxrTmdgfWt8YyBofChtMzs8KGZqYjM+&url=https%3a%2f%2fngplosjournals.pagemajik.ai%2fartanalysis ) to test your figure files. NAAS can convert your figure files to the TIFF file type and meet basic requirements (such as print size, resolution), or provide you with a report on issues that do not meet our requirements and that NAAS cannot fix.

After uploading your figures to PLOS's NAAS tool - <https://spam.inserm.fr/fmlurlsvc/?fewReq=:B:JVA0Pzs7NCh4MzwgPihnajM+PzQ+Pyh9Z2lgb3p7fGszPztrNjs6ajs+ODc7Nz8+azo5PTltPzttbzs+aGg4aDg3Ozc6NmttaCh6Mz85OTY4ODg2Njoof2dqMzg6SjdvNmpUPj0/Njk7Izg6SjdvNmpsPj0/Njk7KHxtfnozbGtgZG9jZ2AgYm9ganxrTmdgfWt8YyBofChtMzs8KGZqYjM+&url=https%3a%2f%2fngplosjournals.pagemajik.ai%2fartanalysis> , NAAS will process the files provided and display the results in the "Uploaded Files" section of the page as the processing is complete.
If the uploaded figures meet our requirements (or NAAS is able to fix the files to meet our requirements), the figure will be marked as "fixed" above. If NAAS is unable to fix the files, a red "failed" label will appear above.
When NAAS has confirmed that the figure files meet our requirements, please download the file via the download option, and include these NAAS processed figure files when submitting your revised manuscript.

Not relevant: all figures were already uploaded as separated .tiff files.


* Please ensure that the paper adheres to the PLOS Data Availability Policy (see <https://spam.inserm.fr/fmlurlsvc/?fewReq=:B:JVA0Pzs7NCh4MzwgPihnajM+PzQ+Pyh9Z2lgb3p7fGszOWxqPmw5OzhoOz9vOW82bzs+PjxsP2ttPTxtOW1oamo+OD44Nzc6Pih6Mz85OTY4ODg2Njoof2dqMzg6SjdvNmpUPj0/Njk7Izg6SjdvNmpsPj0/Njk7KHxtfnozbGtgZG9jZ2AgYm9ganxrTmdgfWt8YyBofChtMz0+KGZqYjM+&url=http%3a%2f%2fjournals.plos.org%2fplosmedicine%2fs%2fdata-availability> ), which requires that all data underlying the study's findings be provided in a repository or as Supporting Information. For data residing with a third party, authors are required to provide instructions with contact information (web or email address) for obtaining the data. Please note that a study author cannot be the contact person for the data. PLOS journals do not allow statements supported by "data not shown" or "unpublished results." For such statements, authors must provide supporting data or cite public sources that include it.

Not relevant: a data availability statement was already provided with a link to apply for access to the UK Biobank data.

* We expect all researchers with submissions to PLOS in which author-generated code underpins the findings in the manuscript to make all author-generated code available without restrictions upon publication of the work. In cases where code is central to the manuscript, we may require the code to be made available as a condition of publication. Authors are responsible for ensuring that the code is reusable and well documented. Please make any custom code available, either as part of your data deposition or as a supplementary file. Please add a sentence to your data availability statement regarding any code used in the study, e.g. "The code used in the analysis is available from Github [URL] and archived in Zenodo [DOI link]" Please review our guidelines at <https://spam.inserm.fr/fmlurlsvc/?fewReq=:B:JVA0Pzs7NCh4MzwgPihnajM+PzQ+Pyh9Z2lgb3p7fGszb2w8OzhqPzc8Oj05NjlrNjk+aDs/Njc7PDw5Pzg/Nj87OmxtPTw8Oyh6Mz85OTY4ODg2Njoof2dqMzg6SjdvNmpUPj0/Njk7Izg6SjdvNmpsPj0/Njk7KHxtfnozbGtgZG9jZ2AgYm9ganxrTmdgfWt8YyBofChtMz0+KGZqYjM+&url=https%3a%2f%2fjournals.plos.org%2fplosmedicine%2fs%2fmaterials-software-and-code-sharing>  and ensure that your code is shared in a way that follows best practice and facilitates reproducibility and reuse. Because Github depositions can be readily changed or deleted, we encourage you to make a permanent DOI'd copy (e.g. in Zenodo) and provide the URL.

We have included four scripts outlining the code and functions relevant to our main analyses and have uploaded these to Github to complement the material provided in the supplements. A statement on code availability has been added to the data availability statement.

**Page 13, lines 719-723:**

**Data Availability**

UKB data is available, upon request, by following the application procedure detailed in https://www.ukbiobank.ac.uk/use-our-data/apply-for-access. This work uses data provided by patients and collected by the NHS as part of their care and support. The codes used in these analyses are available in GitHub [https://github.com/gsilva12/Multimorbidity-Definitions].

* Please ensure that the study is reported according to the appropriate guidelines and include the completed checklist as Supporting Information. When completing the checklist, please use section and paragraph numbers, rather than page numbers. Please add the following statement, or similar, to the Methods: "This study is reported as per [XXXX] guideline (S1 Checklist)."

Done.

FORMATTING - GENERAL
* Abstract: Please structure your abstract using the PLOS Medicine headings (Background, Methods and Findings, Conclusions). Please combine the Methods and Findings sections into one section.

We have merged the Methods and Results section of the abstract so they are now a combined “Methods and Findings” section.

* Please confirm that your abstract complies with our requirements, including format (three sections: Background, Methods and Findings, and Conclusions) and providing all the information relevant to this study type <https://spam.inserm.fr/fmlurlsvc/?fewReq=:B:JVA0Pzs7NCh4MzwgPihnajM+PzQ+Pyh9Z2lgb3p7fGszOWs2Ozw3PTs9OTdrNms5Omw/Pz9qNjY9Pz1oOzZrNzo2Nzk6PTxqNih6Mz85OTY4ODg2Njoof2dqMzg6SjdvNmpUPj0/Njk7Izg6SjdvNmpsPj0/Njk7KHxtfnozbGtgZG9jZ2AgYm9ganxrTmdgfWt8YyBofChtMz0+KGZqYjM+&url=https%3a%2f%2fjournals.plos.org%2fplosmedicine%2fs%2fsubmission-guidelines%23loc-abstract>

We confirm.

* Please ensure that the Introduction ends with a clear description of the study question or hypothesis.

We confirm that the Introductions ends with a clear description of the objective.

* Please ensure that all abbreviations are defined at first use throughout the text.

We confirm that all abbreviations are defined at first use.

* Please confirm that all numbers presented in the abstract are present and identical to numbers presented in the main manuscript text.

All numbers in the abstract are now all present in the manuscript text. They are identical.

* At this stage, we ask that you include a short, non-technical Author Summary of your research to make findings accessible to a wide audience that includes both scientists and non-scientists. The Author Summary should immediately follow the Abstract in your revised manuscript. This text is subject to editorial change and should be distinct from the scientific abstract. Ideally each sub-heading should contain 2-3 single sentence, concise bullet points containing the most salient points from your study. In the final bullet point of 'What Do These Findings Mean?', please include the main limitations of the study in non-technical language. Please see our author guidelines for more information: <https://spam.inserm.fr/fmlurlsvc/?fewReq=:B:JVA0Pzs7NCh4MzwgPihnajM+PzQ+Pyh9Z2lgb3p7fGszO2xrazY9OmtqO209OWs9Nzc9ODc8bTo6Nz86Njs9a21rP2w2ajo9PSh6Mz85OTY4ODg2Njoof2dqMzg6SjdvNmpUPj0/Njk7Izg6SjdvNmpsPj0/Njk7KHxtfnozbGtgZG9jZ2AgYm9ganxrTmdgfWt8YyBofChtMz0+KGZqYjM+&url=https%3a%2f%2fjournals.plos.org%2fplosmedicine%2fs%2frevising-your-manuscript%23loc-author-summary> .

In the author summary, please revise formatting and ensure you use bullet points.

In the author summary, in the final bullet point of 'What Do These Findings Mean?', please include the main limitations of the study in non-technical language.

Please find the following Author summary at the end of the abstract:

**Page 2-3, lines 66-99:**

**Author summary:**

- Using data from 474,397 participants in the UK Biobank, this study compared several count-based and cluster-based definitions of multimorbidity to evaluate how methodological choices influence prevalence estimates and associations with health outcomes and mortality.
- Multimorbidity prevalence varied substantially depending on the operational definition used, whereas associations with mortality remained relatively consistent across count-based approaches; cluster analyses additionally identified smaller, clinically distinct subgroups with particularly poor health profiles and elevated mortality risks.
- These findings highlight that the way multimorbidity is defined strongly shapes epidemiological estimates and population characterization, underscoring the need for future translational studies to determine which definitions are most useful for improving patient management and care in practice.
- The study should also be interpreted in light of certain limitations, including the selected nature of the UK Biobank cohort, the use of some self-reported measures, and the cross-sectional assessment of several health indicators.

* The funding statement should include: specific grant numbers, initials of authors who received each award, URLs to sponsors’ websites. Also, please state whether any sponsors or funders (other than the named authors) played any role in study design, data collection and analysis, the decision to publish, or preparation of the manuscript. If they had no role in the research, include this sentence: “The funders had no role in study design, data collection and analysis, decision to publish, or preparation of the manuscript.”
* All authors must declare their relevant competing interests per the PLOS policy, which can be seen here: <https://spam.inserm.fr/fmlurlsvc/?fewReq=:B:JVA0Pzs7NCh4MzwgPihnajM+PzQ+Pyh9Z2lgb3p7fGsza202aDk7a2w5PG09PTY5Nz43aGxtNztobT0/amttOzo7bGg2ODw/bSh6Mz85OTY4ODg2Njoof2dqMzg6SjdvNmpUPj0/Njk7Izg6SjdvNmpsPj0/Njk7KHxtfnozbGtgZG9jZ2AgYm9ganxrTmdgfWt8YyBofChtMz0+KGZqYjM+&url=https%3a%2f%2fjournals.plos.org%2fplosmedicine%2fs%2fcompeting-interests>  For authors with ties to industry, please indicate whether any of the interests has a financial stake in the results of the current study.
*Please amend your competing interests statement. Funding information should go to the relevant section and include URLs.

We updated statements as follow:

**Page 13, lines 702-718:**

**Competing interests**

The authors have declared that no competing interests exist.

**Acknowledgment**

This project is funded by a grant from “France 2030 ANR-23-PAVH-0006” (https://ppr-autonomie.com/le-ppr-autonomie/) , by IReSP/CNSA as part of the 2023 call for projects for the research support program “Older people, people with disabilities at all stages of life, relatives, and professionals,” supported by the National Solidarity Fund for Autonomy (CNSA) “AAP-2023-APAOB-318115” (https://iresp.net/appel_a_projets/appel-a-projets-2023-analyse-des-politiques-de-lautonomie-de-loffre-medico-sociale-et-des-besoins-des-publics-apaob-session-1/), Fondation vaincre Alzheimer “FR-23063CB” (https://www.vaincrealzheimer.org/) et fondation Alzheimer “1143650” (<https://www.fondation-alzheimer.org/en/>).

We would also like to thank the participants in the UK Biobank cohort, as well as everyone involved in the data collection.

**Role of the funding source**

The funder had no role in the study design, data collection, data analysis, data interpretation, or the writing of the report. The corresponding author had full access to all the data in the study and had final responsibility for the decision to submit for publication.

* Please remove the 'conclusions' subheading from the discussion. Please also remove any other subheadings from the discussion.

Not relevant: no subheadings in the initial discussion.

* Please express the main results with 95% CIs as well as p values. When reporting p values please report as p<0.001 and where higher as the exact p value p=0.002, for example. Throughout, suggest reporting statistical information as follows to improve clarity for the reader "22% (95% CI [13%,28%]; p</=)". Please be sure to define all numerical values at first use.

Not relevant: The main results do not include comparisons based on significance tests in the context of nested groups. We do, however, report 95% confidence intervals.

* Please include page numbers and line numbers in the manuscript file. Use continuous line numbers (do not restart the numbering on each page).

Done: continuous line numbers and page numbers are now in the manuscript file with exception to the Tables/Figures.

* Please cite the reference numbers in square brackets. Citations should precede punctuation.

Done: we have updated the references – they now have in-square brackets and citations precede punctuation.

* Please review your text for claims of novelty or primacy (e.g. 'for the first time') and remove this language. In addition, please check that any use of statistical terms (such as trend or significant) are supported by the data, and if not please remove them.

Done: we have reviewed the manuscript to remove any improper use of statistical terms.

* Please remove the 'conclusions' subheading from the discussion. Please also remove any other subheadings from the discussion.

Not relevant: no subheadings in the initial discussion.

* In the last sentence of the Abstract Methods and Findings section, please describe the main limitation(s) of the study's methodology.

Done: this last sentence has been added:

**Abstract, page 2, lines 58-59:**

Findings should be interpreted in light of the selected nature of the UK Biobank cohort and the cross-sectional assessment of several health indicators.

"* PLOS Medicine requires that the de-identified data underlying the specific results in a published article be made available, without restrictions on access, in a public repository or as Supporting Information at the time of article publication, provided it is legal and ethical to do so. Please see the policy at

<https://spam.inserm.fr/fmlurlsvc/?fewReq=:B:JVA0Pzs7NCh4MzwgPihnajM+PzQ+Pyh9Z2lgb3p7fGszOWxqPmw5OzhoOz9vOW82bzs+PjxsP2ttPTxtOW1oamo+OD44Nzc6Pih6Mz85OTY4ODg2Njoof2dqMzg6SjdvNmpUPj0/Njk7Izg6SjdvNmpsPj0/Njk7KHxtfnozbGtgZG9jZ2AgYm9ganxrTmdgfWt8YyBofChtMz0+KGZqYjM+&url=http%3a%2f%2fjournals.plos.org%2fplosmedicine%2fs%2fdata-availability>  

and FAQs at

<https://spam.inserm.fr/fmlurlsvc/?fewReq=:B:JVA0Pzs7NCh4MzwgPihnajM+PzQ+Pyh9Z2lgb3p7fGszbzlsNmo2bTY4a2s4Nj4+ODg6O2g+Oz4/PWo6bz8/OTk3aGg6bD48Oyh6Mz85OTY4ODg2Njoof2dqMzg6SjdvNmpUPj0/Njk7Izg6SjdvNmpsPj0/Njk7KHxtfnozbGtgZG9jZ2AgYm9ganxrTmdgfWt8YyBofChtMz0+KGZqYjM+&url=http%3a%2f%2fjournals.plos.org%2fplosmedicine%2fs%2fdata-availability%23loc-faqs-for-data-policy>   "

"* PLOS defines the “minimal data set” to consist of the data set used to reach the conclusions drawn in the manuscript with related metadata and methods, and any additional data required to replicate the reported study findings in their entirety. Authors do not need to submit their entire data set, or the raw data collected during an investigation. Please submit the following data:

The values behind the means, standard deviations and other measures reported;
The values used to build graphs;
The points extracted from images for analysis."

"* The Data Availability Statement (DAS) requires revision. For each data source used in your study:

a) If the data are freely or publicly available, note this and state the location of the data: within the paper, in Supporting Information files, or in a public repository (include the DOI or accession number).

b) If the data are owned by a third party but freely available upon request, please note this and state the owner of the data set and contact information for data requests (web or email address). Note that a study author cannot be the contact person for the data.

c) If the data are not freely available, please describe briefly the ethical, legal, or contractual restriction that prevents you from sharing it. Please also include an appropriate contact (web or email address) for inquiries (again, this cannot be a study author).

Data are not freely available. A data availability statement is provided with a link to submit for data access.

**Page 13, lines 719-723:**

**Data Availability**

UKB data is available, upon request, by following the application procedure detailed in https://www.ukbiobank.ac.uk/use-our-data/apply-for-access. This work uses data provided by patients and collected by the NHS as part of their care and support. The codes used in these analyses are available in GitHub [https://github.com/gsilva12/Multimorbidity-Definitions].

* For studies in which a novel model is central to the manuscript's findings, as is the case here, authors are responsible for providing the source code needed to replicate the study's findings in a repository (such as GitHub, SourceForge or Bitbucket) or a cloud computing service (such as Code Ocean). Protection of authors’ intellectual property will not be cause for exception. Please explain in the manuscript’s Data Availability Statement how readers can access the shared code.

We have included four scripts outlining the code and functions relevant to our main analyses and have uploaded these to Github to complement the material provided in the supplements. A statement on code availability has been added to the data availability statement.

**Page 13, lines 719-723:**

**Data Availability**

UKB data is available, upon request, by following the application procedure detailed in https://www.ukbiobank.ac.uk/use-our-data/apply-for-access. This work uses data provided by patients and collected by the NHS as part of their care and support. The codes used in these analyses are available in GitHub [https://github.com/gsilva12/Multimorbidity-Definitions].

* Thank you for agreeing to make your data available. At this time, please provide the link to the data repository and accession numbers required for access.

Please see answer to previous comment.

* Please include the statement on code availability in the data availability statement.

Please see answer to previous comment.

* Please provide the name(s) of the institutional review board(s) that provided ethical approval.

Not relevant: this statement was already provided. Please see statement below:

**Page 5, lines 281-282:**

Written informed consent was obtained from all participants and ethical approval for the UKB study was granted by the NHS North West Centre for Research Ethics Committee (reference 11/NW/0382). This research used UKB Resource under data sharing application 96856.

FIGURES AND TABLES
* Please provide titles and legends for all figures and tables (including those in Supporting Information files).

Done: List of titles and legends for all figures and tables including supporting information files has been added at the end of document.

* Please define all abbreviations used in each figure/table (including those in Supporting Information files).

All abbreviations used in figures and tables have been defined.

* Please consider avoiding the use of red and green in order to make your figure more accessible to those with color blindness.

Done: figures have been updated and saved as .tiff.

* Please define all elements of box plots in the figure caption - center line, box limits and whiskers.

Not relevant: no boxplots in manuscript.

* Figures cannot be reproduced from other sources that are not CC-BY. Please provide a different figure.

Not relevant: all numbers required to reproduce plot are available in supplementary file.

* Please provide titles and legends for all figures and tables (including those in Supporting Information files).

Please define all acronyms used in each figure or table in its corresponding legend.

Not relevant: all abbreviations used in figures and tables have been defined.

* For figures containing blots of gels please ensure that you have complied with our figures requirements <https://spam.inserm.fr/fmlurlsvc/?fewReq=:B:JVA0Pzs7NCh4MzwgPihnajM+PzQ+Pyh9Z2lgb3p7fGszOTo5bzZqPW05aj5oOTloN2s+O2g+PWw7P2g8Pzw+Nmo+Omw3bD0/bCh6Mz85OTY4ODg2Njoof2dqMzg6SjdvNmpUPj0/Njk7Izg6SjdvNmpsPj0/Njk7KHxtfnozbGtgZG9jZ2AgYm9ganxrTmdgfWt8YyBofChtMz0+KGZqYjM+&url=http%3a%2f%2fjournals.plos.org%2fplosmedicine%2fs%2ffigures> .

Not relevant: no blots of gels.

* Please convert any pie charts to another data representation for example a table, or other type of graph.

Not relevant: no pie charts in manuscript.

* Please ensure that where relevant figures include 95% CIs.

Figure 2 includes 95% confidence intervals. Figure 1 has an indicator for whether or not the regression coefficient was statistically significant – displaying the confidence intervals here would not add any additional information to this since the range (which was selected to ensure consistency of the scale across the five standardized blood-based measures) is too large to reflect important differences. Instead, the 95% CI for the values in Figure 1 are detailed in Table S9.

* Please show graph axes beginning at zero. If this is not possible, please show a break in the axis.

Graphs include the 0 (or 1 for ratio).

* When a p value is given, please specify the statistical test used to determine it in the legend.

Not relevant.

* Please convert any stacked bar charts to another data representation for example a table, or other type of graph.

Not relevant: no stacked bar chart in manuscript

* In the Kaplan-Meier curve(s) please provide the number at risk for each time interval.

Not relevant: no Kaplan-Meier curve in manuscript.

* Please consider avoiding the use of red and green in order to make your figure more accessible

Done: figures were updated and saved as .tiff.

* Where data points are discrete, please ensure that they are depicted in the figures as discrete data and not as a continuous line.

Not relevant: no figures displaying trends or continuous lines

* Please confirm that the appropriate usage rights apply to the use of this map. Please see our guidelines for map images: <https://spam.inserm.fr/fmlurlsvc/?fewReq=:B:JVA0Pzs7NCh4MzwgPihnajM+PzQ+Pyh9Z2lgb3p7fGszOjo9bD05bzY6aDlvOTs2OT48N29vbDprbzk+OGg5OTc4b29vP2prNyh6Mz85OTY4ODg2Njoof2dqMzg6SjdvNmpUPj0/Njk7Izg6SjdvNmpsPj0/Njk7KHxtfnozbGtgZG9jZ2AgYm9ganxrTmdgfWt8YyBofChtMz0+KGZqYjM+&url=https%3a%2f%2fjournals.plos.org%2fplosmedicine%2fs%2ffigures%23loc-maps>

Not relevant: no map in manuscript.

* Please provide the unadjusted comparisons as well as the adjusted comparisons in all relevant Tables

We have included the unadjusted results as a supplement (Tables S8 and S12).

* Please specify the variables controlled for in all relevant Tables

All tables and figures based on adjusted analyses list the variables controlled for.

* We note you have included images of branded products. Please confirm you have appropriate permission or you may wish to remove these images. For more information, please see our guidelines: <https://spam.inserm.fr/fmlurlsvc/?fewReq=:B:JVA0Pzs7NCh4MzwgPihnajM+PzQ+Pyh9Z2lgb3p7fGszNzc5bDo2Nz42OzttOG8+P288bTtqb206b2o6PT0+OWg5PTZrNjc4bSh6Mz85OTY4ODg2Njoof2dqMzg6SjdvNmpUPj0/Njk7Izg6SjdvNmpsPj0/Njk7KHxtfnozbGtgZG9jZ2AgYm9ganxrTmdgfWt8YyBofChtMz0+KGZqYjM+&url=https%3a%2f%2fjournals.plos.org%2fplosmedicine%2fs%2ffigures%23loc-licenses-and-copyright>  and <https://spam.inserm.fr/fmlurlsvc/?fewReq=:B:JVA0Pzs7NCh4MzwgPihnajM+PzQ+Pyh9Z2lgb3p7fGszbzpvOGpra283aGo4a2w6Omw4Ozc7OGo9Pmw4bW83Nm88Pjs5bWtvOCh6Mz85OTY4ODg2Njoof2dqMzg6SjdvNmpUPj0/Njk7Izg6SjdvNmpsPj0/Njk7KHxtfnozbGtgZG9jZ2AgYm9ganxrTmdgfWt8YyBofChtMz0+KGZqYjM+&url=https%3a%2f%2fjournals.plos.org%2fplosmedicine%2fs%2flicenses-and-copyright>

Not applicable: no branded products.

* Please consider if moving some of the figures shared in the supplementary information into the main text would aid the reader.

There is only one supplementary figure (the flow chart) which we believe is correctly placed in the supplementary material. We confirm the figures in the other supplementary methods document related to clustering also belong in this document, not the main text.

* Please include an acknowledgment of study participants.

Please see the following statement:

**Page 13, lines 713-714:**

We would also like to thank the participants in the UK Biobank cohort, as well as everyone involved in the data collection.

SUPPLEMENTARY MATERIAL
* Please note that supplementary material will be posted as supplied by the authors. Therefore, please amend it according to the relevant comments outlined here.

Done.

* Please cite your Supporting Information as outlined here: <https://spam.inserm.fr/fmlurlsvc/?fewReq=:B:JVA0Pzs7NCh4MzwgPihnajM+PzQ+Pyh9Z2lgb3p7fGszajk6bzc3Pjc3NzY2OT9tbDlsaj84OzY4PWo8N2psP2o6OGs/aD03Nih6Mz85OTY4ODg2Njoof2dqMzg6SjdvNmpUPj0/Njk7Izg6SjdvNmpsPj0/Njk7KHxtfnozbGtgZG9jZ2AgYm9ganxrTmdgfWt8YyBofChtMz0+KGZqYjM+&url=https%3a%2f%2fjournals.plos.org%2fplosmedicine%2fs%2fsupporting-information>

We have edited the references to the Supporting Information so they are now in the desired “S” format.

REFERENCES

* PLOS uses the numbered citation (citation-sequence) method and first six authors, et al.

We confirm having updated the refences to the style used by PLOS.

* Please ensure that journal name abbreviations match those found in the National Center for Biotechnology Information (NCBI) databases (https://spam.inserm.fr/fmlurlsvc/?fewReq=:B:JVA0Pzs7NCh4MzwgPihnajM+PzQ+Pyh9Z2lgb3p7fGszPDk7a2s8aDpvN2w+Pjxqa2xoPzZoazo4OWw6bGxraGhqPDo2Pjo8aih6Mz85OTY4ODg2Njoof2dqMzg6SjdvNmpUPj0/Njk7Izg6SjdvNmpsPj0/Njk7KHxtfnozbGtgZG9jZ2AgYm9ganxrTmdgfWt8YyBofChtMzs/KGZqYjM+&url=http%3a%2f%2fwww.ncbi.nlm.nih.gov%2fnlmcatalog%2fjournals ), and are appropriately formatted and capitalised.

We confirm that the journal name abbreviations are appropriately formatted and capitalised.

* Where website addresses are cited, please include the complete URL and specify the date of access (e.g. [accessed: 12/06/2023]).

Not applicable.

* Please also see <https://spam.inserm.fr/fmlurlsvc/?fewReq=:B:JVA0Pzs7NCh4MzwgPihnajM+PzQ+Pyh9Z2lgb3p7fGszNz4/Nmw5bz89aDZsPzlqPmo5aDw5Pz5qbWhtPTdsNjxrOT0+bDxsPyh6Mz85OTY4ODg2Njoof2dqMzg6SjdvNmpUPj0/Njk7Izg6SjdvNmpsPj0/Njk7KHxtfnozbGtgZG9jZ2AgYm9ganxrTmdgfWt8YyBofChtMz0+KGZqYjM+&url=https%3a%2f%2fjournals.plos.org%2fplosmedicine%2fs%2fsubmission-guidelines%23loc-references>  for further details on reference formatting.

STUDY TYPE-SPECIFIC REQUESTS:


OBSERVATIONAL STUDIES

* Abstract: Please include the study design, population and setting, number of participants, years during which the study took place (enrollment and follow up), length of follow up, and main outcome measures.

Abstract include the relevant information.

* For all observational studies, in the manuscript text, please indicate: (1) the specific hypotheses you intended to test, (2) the analytical methods by which you planned to test them, (3) the analyses you actually performed, and (4) when reported analyses differ from those that were planned, transparent explanations for differences that affect the reliability of the study's results. If a reported analysis was performed based on an interesting but unanticipated pattern in the data, please be clear that the analysis was data-driven.

Relevant information is included in the manuscript.

* Please ensure that the study is reported according to the STROBE (or appropriate STROBE extension) guideline (available from: <https://spam.inserm.fr/fmlurlsvc/?fewReq=:B:JVA0Pzs7NCh4MzwgPihnajM+PzQ+Pyh9Z2lgb3p7fGszbGw2O20+Ozk8bWtoa283PTprbzw2OGhqbW09NzprOjk5Pz5oaDptOCh6Mz85OTY4ODg2Njoof2dqMzg6SjdvNmpUPj0/Njk7Izg6SjdvNmpsPj0/Njk7KHxtfnozbGtgZG9jZ2AgYm9ganxrTmdgfWt8YyBofChtMz09KGZqYjM+&url=https%3a%2f%2fwww.equator-network.org%2freporting-guidelines%2fstrobe> ) and include the completed STROBE (or STROBE extension) checklist as Supporting Information. Please add the following statement, or similar, to the Methods: "This study is reported as per the Strengthening the Reporting of Observational Studies in Epidemiology (STROBE) guideline (S1 Checklist)." When completing the checklist, please use section and paragraph numbers, rather than page numbers.

Strobe checklist has been provided in supplementary.

* For all observational studies, in the manuscript text, please indicate: (1) the specific hypotheses you intended to test, (2) the analytical methods by which you planned to test them, (3) the analyses you actually performed, and (4) when reported analyses differ from those that were planned, transparent explanations for differences that affect the reliability of the study's results. If a reported analysis was performed based on an interesting but unanticipated pattern in the data, please be clear that the analysis was data driven.

Please see answer to previous point.

* Please state in the Methods section whether the study had a prospective protocol or analysis plan. If a prospective analysis plan (from your funding proposal, IRB or other ethics committee submission, study protocol, or other planning document written before analyzing the data) was used in designing the study, please include the relevant document(s) with your revised manuscript as a Supporting Information file to be published alongside your study and cite it in the Methods section. A legend for this file should be included at the end of your manuscript. If no such document exists, please make sure that the Methods section transparently describes when analyses were planned, and when/why any data-driven changes to analyses took place. Changes in the analysis, including those made in response to peer review comments, should be identified as such in the Methods section of the paper, with rationale.

Not relevant : no prospective registered protocol is available.

* Did your study have a prospective protocol or analysis plan? Please state this (either way) early in the Methods section.

a) If a prospective analysis plan (from your funding proposal, IRB or other ethics committee submission, study protocol, or other planning document written before analyzing the data) was used in designing the study, please include the relevant prospectively written document with your revised manuscript as a Supporting Information file to be published alongside your study, and cite it in the Methods section. A legend for this file should be included at the end of your manuscript.

b) If no such document exists, please make sure that the Methods section transparently describes when analyses were planned, and when/why any data-driven changes to analyses took place.

c) In either case, changes in the analysis-- including those made in response to peer review comments-- should be identified as such in the Methods section of the paper, with rationale.

No prospective registered protocol is available.

* Your study is observational and therefore causality cannot be inferred. Please remove language that implies causality and refer to associations instead.

We agree with this statement. We acknowledge that causality cannot be inferred in the discussion section.

* Please confirm that your title complies with PLOS Medicine's style. Your title must be nondeclarative and not a question. It should begin with main concept if possible. "Effect of" should be used only if causality can be inferred, i.e., for an RCT. Please place the study design ("A randomized controlled trial," "A retrospective study," "A modelling study," etc.) in the subtitle (ie, after a colon).

We modified the title as following:

Comparing count-based and clustering definitions of multimorbidity: implications for prevalence, health profiles, and mortality, a prospective cohort study in the UK Biobank.

* At this stage, we ask that you include a short, non-technical Author Summary of your research to

make findings accessible to a wide audience that includes both scientists and non-scientists. The

Author Summary should immediately follow the Abstract in your revised manuscript. This text is

subject to editorial change and should be distinct from the scientific abstract. Ideally each sub-heading

should contain 2-3 single sentence, concise bullet points containing the most salient points from your

study. In the final bullet point of 'What Do These Findings Mean?', please include the main limitations

of the study in non-technical language. Please see our author guidelines for more information:

We include an Author summary, after abstract, as follow:

**Author Summary**

**Why was the study done?**

- Multimorbidity is associated with poorer health outcomes, greater healthcare use, and higher mortality, but there is no consensus on how it should be defined and measured across studies.
- Although both count-based and cluster-based approaches are widely used, few studies have systematically compared these definitions within the same population to assess how methodological choices influence prevalence estimates but also associations with health outcomes and mortality.

**What did the researchers do and find?**

- Using data from 474,397 UK Biobank participants, we compared six count-based definitions of multimorbidity and a cluster-based approach, and examined prevalence of multimorbidity, their associations with health outcomes, blood-based measures, and 3- and 10-year mortality.
- Multimorbidity prevalence varied substantially depending on the definition used, whereas associations with mortality were relatively consistent across count-based definitions; clustering approaches additionally identified smaller subgroups with distinct disease profiles, poorer health status, and elevated mortality risks.

**What do these findings mean?**

- These findings highlight that the way multimorbidity is defined strongly shapes epidemiological estimates and population characterization, underscoring the need for future translational studies to determine which definitions are most useful for improving patient management and care in practice.
- The study should also be interpreted in light of certain limitations, including the selected nature of the UK Biobank cohort, the use of some self-reported measures, and the cross-sectional assessment of several health indicators.
